# Assessing the genetic variation of *Ty*-*1* and *Ty*-*3* alleles conferring resistance to tomato yellow leaf curl virus in a broad tomato germplasm

**DOI:** 10.1007/s11032-015-0329-y

**Published:** 2015-05-26

**Authors:** Myluska Caro, Maarten G. Verlaan, Olga Julián, Richard Finkers, Anne-Marie A. Wolters, Samuel F. Hutton, John W. Scott, Richard Kormelink, Richard G. F. Visser, Maria J. Díez, Ana Pérez-de-Castro, Yuling Bai

**Affiliations:** Wageningen UR Plant Breeding, Wageningen University and Research Centre, Droevendaalsesteeg 1, 6708 PB Wageningen, The Netherlands; Graduate School Experimental Plant Sciences, Wageningen University and Research Centre, Droevendaalsesteeg 1, 6708 PB Wageningen, The Netherlands; Instituto Universitario de Conservación y Mejora de la Agrodiversidad Valenciana (COMAV) (University Institute for Conservation and Improvement of Agrobiodiversity), Ciudad Politécnica de la Innovación (CPI), Universitat Politècnica de València, Camino de Vera, s/n Edificio 8E escalera J, 46022 Valencia, Spain; Gulf Coast Research and Education Center, University of Florida, 14625 CR 672, Wimauma, FL 33598 USA; Laboratory of Virology, Wageningen University and Research Centre, Droevendaalsesteeg 1, 6708 PB Wageningen, The Netherlands; Rijk Zwaan Breeding B.V., Burgemeester Crezéélaan 40, 2678 KX De Lier, The Netherlands

**Keywords:** Breeding, Resistance, RNA-dependent RNA polymerase (RDR), Tomato, Tomato yellow leaf curl virus (TYLCV), Virus-induced gene silencing (VIGS)

## Abstract

Tomato yellow leaf curl virus (TYLCV) hampers tomato production worldwide. Our previous studies have focussed on mapping and ultimately cloning of the TYLCV resistance genes *Ty*-*1* and *Ty*-*3*. Both genes are derived from *Solanum chilense* and were shown to be allelic. They code for an RNA-dependent RNA polymerase (RDR) belonging to the RDRγ type defined by a DFDGD catalytic domain. In this study, we first fine-mapped the TYLCV resistance in *S. chilense* LA1932, LA1960 and LA1971. Results showed that chromosomal intervals of the causal genes in these TYLCV-resistant accessions overlap and cover the region where *Ty*-*1/Ty*-*3* is located. Further, virus-induced gene silencing was used to silence *Ty*-*1*/*Ty*-*3* in tomato lines carrying TYLCV resistance introgressed from *S. chilense* LA1932, LA1938 and LA1971. Results showed that silencing *Ty*-*1/Ty*-*3* compromised the resistance in lines derived from *S. chilense* LA1932 and LA1938. The LA1971-derived material remained resistant upon silencing *Ty*-*1/Ty*-*3*. Further, we studied the allelic variation of the *Ty*-*1/Ty*-*3* gene by examining cDNA sequences from nine *S. chilense*-derived lines/accessions and more than 80 tomato cultivars, landraces and accessions of related wild species. The DFDGD catalytic domain of the *Ty*-*1/Ty*-*3* gene is conserved among all tomato lines and species analysed. In addition, the 12 base pair insertion at the 5-prime part of the *Ty*-*1/Ty*-*3* gene was found not to be specific for the TYLCV resistance allele. However, compared with the susceptible *ty*-*1* allele, the *Ty*-*1/Ty*-*3* allele is characterized by three specific amino acids shared by seven TYLCV-resistant *S. chilense* accessions or derived lines. Thus, *Ty*-*1/Ty*-*3*-specific markers can be developed based on these polymorphisms. Elevated transcript levels were observed for all tested *S. chilenseRDR* alleles (both *Ty*-*1* and *ty*-*1* alleles), demonstrating that elevated expression level is not a good selection criterion for a functional *Ty*-*1/Ty*-*3* allele.

**Electronic supplementary material:**

The online version of this article (doi:10.1007/s11032-015-0329-y) contains supplementary material, which is available to authorized users.

## Introduction

Tomato yellow leaf curl virus (TYLCV), a *begomovirus* of the *geminiviridae* family, is a phloem-limited single-stranded DNA virus that is vectored by the whitefly (*Bemisia tabaci*). TYLCV is one of the causal viruses of tomato yellow leaf curl disease (TYLCD). In the last two decades, TYLCD has been a major constraint on tomato (*Solanum lycopersicum*) production in many warm and (sub) tropical regions worldwide, and nowadays, it is still a huge problem for many farmers. Tomato plants affected by TYLCD show yellowing and curling of apical leaves, and when plants are severely affected, flowers are abscised and plants stop growing completely (Cohen and Lapidot 2007). Controlling vector whitefly populations is expensive, labour intensive, and often ineffective; thus, using resistant tomato cultivars is a good solution to control TYLCV. No resistance has yet been described in cultivated tomato, and breeders have screened wild tomato relatives to identify resistance sources from which resistance loci have been introgressed (Ji et al. 2007; Vidavski 2007). To date, six TYLCV resistance/tolerance genes have been described, *Ty*-*1* to *Ty*-*6* (Figure S1) (Zamir et al. 1994; Hanson et al. 2006; Ji et al. 2007; Anbinder et al. 2009; Ji et al. 2009; Hutton et al. 2012; Hutton and Scott 2013). Most of these loci originated from accessions of *Solanumchilense*. The *Ty*-*1* gene is derived from LA1969 and the *Ty*-*3* gene from LA2779. Both *Ty*-*1* and *Ty*-*3* are located on the long arm of tomato chromosome 6 and have been shown to be allelic (Verlaan et al. 2011, 2013). LA1932 is reported to carry an allele at this locus, *Ty*-*3a*, (Scott et al. 1996; Ji et al. 2007) and is also the donor of *Ty*-*4*, which maps to chromosome 3 (Ji et al. 2009). *Ty*-*6* is derived from LA2779 (also the donor of the *Ty*-*3* allele) and recently mapped to chromosome 10 (Hutton and Scott 2013). The other two known TYLCV resistance genes do not originate from *S. chilense*. *Ty*-*2* was introgressed from *S. habrochaitesf. glabratum* accession “B6013” and is located on chromosome 11 (Yang et al. 2014). *Ty*-*5* was first described in TY172, a breeding line said to be derived from crosses of four *S. peruvianum* accessions. However, whether the *Ty*-*5* originated from *S. peruvianum* is still in debate; there is recent evidence that this gene is recessively inherited and resulted from a loss-of-function mutation that likely occurred in cultivated tomato (Hutton et al. 2012; Levin et al. 2013). *Ty*-*5* maps on chromosome 4, and because of its recessive nature, the symbol *ty*-*5* was proposed to refer to this gene (Hutton et al. 2012).

Recently, we cloned the *Ty*-*1* and *Ty*-*3* genes (Verlaan et al. 2013), which code for RNA-dependent RNA polymerases (RDR) belonging to the RDRγ type. RDRs are defined by a conserved catalytic domain, DFDGD for the RDRγ, and DLDGD for the RDRα type. In *Arabidopsis thaliana*, the RDRα type has been well studied and shown to be involved in stress responses, pathogen resistance, female gamete formation, and transgene silencing among many other functions (excellently reviewed in Willmann et al. 2011). In contrast to the RDRα type, no functions for RDRγ have been described in literature. Because the RDRα type is known to be involved in the amplification of the siRNA signal, it is possible that the RDRγ type has a similar function in siRNA amplification. Our results suggested that *Ty*-*1*, representative for the RDRγ type and a novel class of *R*-genes, confers resistance through enhanced transcriptional gene silencing (Butterbach et al. 2014).

In *S. chilense*, multiple accessions have been described as symptomless after TYLCV inoculation. For some accessions, including LA1960, LA1971 and LA1938, the causal genes for resistance were mapped to chromosome 6 in the chromosome region where *Ty*-*1* is located, suggesting allelism to *Ty*-*1*/*Ty*-*3* (Pérez de Castro et al. 2013; Agrama and Scott 2006, Hutton and Scott, unpublished data). For other accessions, the causal genes have not been mapped (Pico et al. 1999). Thus, it is intriguing whether the TYLCV resistance in various *S. chilense* accessions is governed by allelic variants of the *Ty*-*1/Ty*-*3* gene.

The aim of this study was to explore the allelic variation of *Ty*-*1*/*Ty*-*3* in wild tomato relatives, with the focus on *S. chilense* accessions. In a previous study, we showed that the susceptible *ty*-*1* allele differs from the resistant *Ty*-*1*/*Ty*-*3* allele at multiple amino acid positions (Verlaan et al. 2013). The most striking difference was a 4 amino acid insertion near the start of the protein in the *Ty*-*1*/*Ty*-*3* allele, while in the catalytic domain, there were no differences. In this study, we compare the full-length cDNA sequence of seven different tomato introgression lines that have *S. chilense*-derived TYLCV resistance and two *S. chilense* accessions to identify *Ty*-*1/Ty*-*3*-specific polymorphisms. The insertion and catalytic domain of the protein are also explored in 87 lines/accessions of tomato and its wild relatives to see whether these *S. chilense* features are unique. Further, we silenced the alleles of the *Ty*-*1*/*Ty*-*3* gene in several TYLCV-resistant tomato lines carrying introgressions from different *S. chilense* accessions to check whether the silencing compromises the TYLCV resistance in these lines.

## Results

### Potential allelic variants in multiple *S. chilense* accessions

**Fig. 1:**
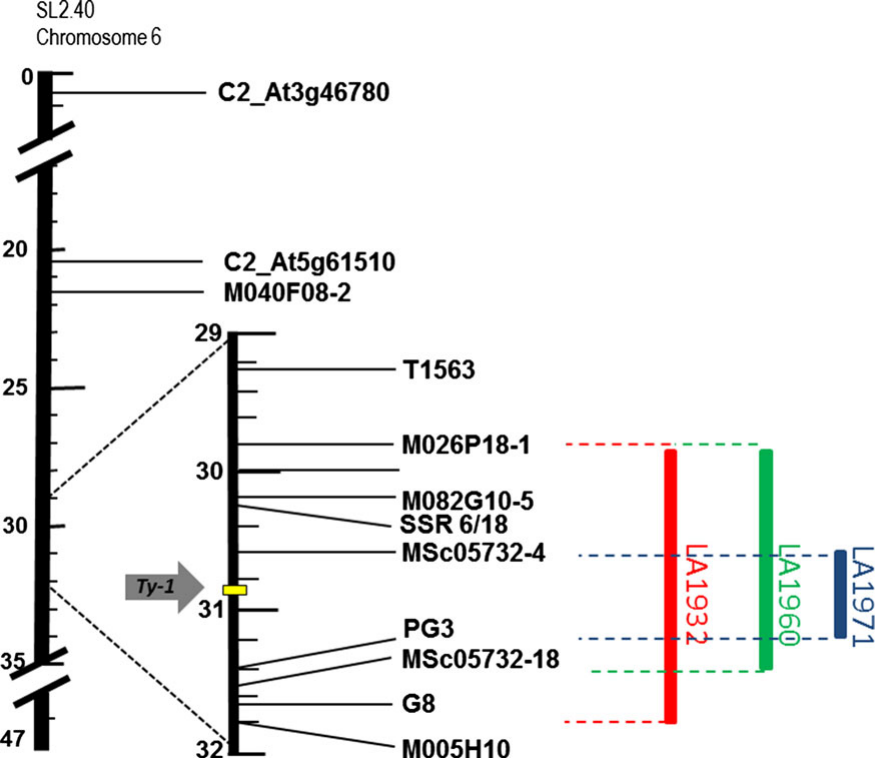
Schematic physical map of the *Ty*-*1* region showing locations of resistance loci in *Solanum chilense* LA1932, LA1960 and LA1971. Numbers represent millions of basepairs. Position of markers is based on BLAST results on the tomato WGS 2.40 chromosomes database

In a previous study, TYLCV resistance in *S. chilense* accessions LA1932, LA1960 and LA1971 was studied and shown to be controlled by a major dominant gene located on chromosome 6 (Pérez de Castro et al. 2013), indicating that the causal genes in these accessions are likely allelic to *Ty*-*1*. To further verify the localization of the causal genes in the *Ty*-*1* interval, fine-mapping was performed in this study using F_2_ and backcross mapping populations derived from these three accessions (Table 1, Figure S2). Markers covering the *Ty*-*1* region between markers C2_At3g46780 and M-M005H10 on chromosome 6 were applied to genotype recombinants identified between these two markers (Fig. 1, Table S1 and S2). Of 844 plants from these populations, 66 recombinants were obtained and tested for TYLCV resistance (Table S1). In Families 1 and 2 that are derived from LA1932, 22 recombinants were found. The most informative recombinants are no. 6, 17 and 18 (Table 2, Table S1). These three recombinants showed clear TYLCV symptoms (Figure S3), indicating that the TYLCV resistance locus from LA1932 is located between markers M-M026P18 and G8 since all three lacked any *S.chilense* introgression within this interval, but each had such an introgression on one or the other side of the interval. In a similar way, the resistance loci in LA1960 and LA1971 were defined. In the Family 3 derived from LA1960, two informative recombinants, i.e. 7 and 10, showed that the locus is bordered by markers M-M026P18 and MSc05732-18 (Table 2, Table S1). Families 4 and 5 (both derived from LA1971) produced three highly informative recombinants, 13, 21 and 22, which placed the LA1971 resistance locus between markers MSc05732-4 and PG3 (Table 2, Table S1). All three chromosomal intervals of the analysed families overlap and cover the *Ty*-*1/Ty*-*3* locus (Fig. 1).

**Table 1:** Information on progenies derived from *S. chilense* accessions LA1932, LA1960 and LA1971

Family^a^	Susceptible parent	Resistant parent^b^	*Solanum chilense* accession	Number of plants per genotype used in the disease test^c^					
				P_1_	P_2_	F_1_	F_2_	BC_1S_	BC_1R_
1	Fortuna C	(FC × BC_1_S_4_)S_2_	LA 1932	25	23		155		
2	Fortuna C	(FC × BC_1_S_4_)S_2_		25	18		113		
3	Fortuna C	BC_1_S_4_	LA 1960	25	9		155		
4	Fortuna C	BC_1_S_4_	LA 1971	25	0		116		
5	Fortuna C	BC_1_S_4_		25	8	9	143	89	73

^a^Families 1 and 2, as well as 4 and 5 were derived from different plants of the same population

^b^FC: susceptible parent; BC: backcross generation with selection; S: selfing generation with selection (see Fig. 1 for details). Subscript numbers after BC and S indicate generations

^c^P_1_: susceptible parent; P_2_: resistant parent; BC_1S_: backcross to the susceptible parent; BC_1R_: backcross to the resistant parent

**Table 2:** Informative recombinants identified on chromosome 6 between markers T1563 and M-M005H10, a region covering the *Ty*-*1* interval in *Solanum chilense*-derived populations

*Solanum chilense*accession	Recombinant^a^		Markers^b^									
	Family	Number	T1563	M-M026P18	M-M082G10	SSR6/18	MSc05732-4	PG3	MSc05732-18	G8	M-M005H10	Phenotype^c^
LA1932	1	**6**	a	a	a	a	a	a	a	h	h	S
	2	**17**	h	h	a	a	a	a	a	a	a	S
	2	**18**	h	h	a	a	a	a	a	a	a	S
LA1960	3	**7**	h	h	a	a	a	a	a	a	a	S
	3	**10**	h	h	h	h	h	nd	a	a	a	R
LA1971	4	**13**	h	h	h	h	h	a	a	a	a	S
	5a	**21**	a	a	a	a	a	h	h	h	h	S
	5a	**22**	a	a	a	a	a	h	h	h	h	R

Bold value indicates the number of recombinants found in each population

^a^See Table S1 for details. The recombinant number is the same as in Table S1

^b^a: Homozygous for *S. lycopersicum* allele; b: Homozygous for *S. chilense* allele; h: heterozygous

*nd* not determined

^c^R: resistant (disease score 0–1); S: susceptible (disease score 2–4)

For the resistance in LA1938, breeding practice showed that there is a linkage of the resistance from LA1938 with the self-pruning (*sp*) locus, which is located on the long arm of chromosome 6 (Agrama and Scott 2006). Suppression of recombination made breakage of this linkage difficult. Using an F_2_ of the cross between LA1938-derived line F11E976-BK (also known as Fla.976) and a susceptible tomato cultivar, the resistance was shown to be linked with the *Ty*-*3*-associated markers used in Ji et al. (2007).

### *Ty*-*1/Ty*-*3* alleles in multiple *S. chilense*-derived introgression lines

To assess whether TYLCV resistance derived from the above studied *S. chilense* accessions was based on *Ty*-*1*/*Ty*-*3* alleles, a VIGS approach was applied to silence the *Ty*-*1* gene with the TRV2-180 and/or TRV2-190 silencing constructs as described in Verlaan et al. (2013). Tomato introgression lines derived from these accessions were used for VIGS (Table 3). Tomato Moneymaker (MM) plants were used as a susceptible control. Two weeks after TYLCV inoculation, all MM plants showed typical TYLCV symptoms, while plants of tomato introgression lines infiltrated with the empty vector (EV) remained symptom free (Figure S3). In the lines derived from *S. chilense* LA1932 and LA1938, all but two out of 31 plants infiltrated with TRV2-180/190 silencing constructs showed typical symptoms (Table 3). The two symptom-free plants may have been escaped from the TYLCV infection or due to a low silencing level. Together with the mapping data (Fig. 1), the collapse of TYLCV resistance by VIGS clearly indicates that resistance in the tested lines derived from *S. chilense* LA1932 and LA1938 is based on *Ty*-*1/Ty*-*3*.

**Table 3:** Silencing *Ty*-*1* compromises TYLCV resistance in multiple *Solanum chilense*-derived lines

Resistance source	Tomato line	Reported gene	Silencing construct			Control		
			Plants tested	S^a^	R^a^	Plants tested	S	R
*S. chilense* LA1932	1538	*Ty*-*3*	16	**14**	2	4	**0**	4
	B26	*Ty*-*3*	4	**4**	0	2	**0**	2
*S. chilense* LA1938	Fla.976	*Ty*-*3*	11	**11** ^b^	0	5	**0**	5
*S. chilense* LA1971	1594	Unknown	15	**0**	15	5	**0**	5
*S. chilense* LA2779	Fla.8680	*Ty*-*3*	14	**13** ^b^	1	3	**0**	3
	Fla.8383	*Ty*-*6*	5	**0**	5	2	**0**	2

Bold value indicates the number of recombinants found in each population

VIGS constructs TRV2-180 and TRV2-190 targeting different parts of the gene were used to silence the *Ty*-*1* allele (Verlaan et al. 2013). Empty TRV vector were used as control

^a^Susceptible (S): showing TYLCV symptoms; disease score 2–4, see Figure S2. Resistant (R): symptom-free; disease score 0–1

^b^All susceptible plants had a disease score of 2

In contrast, plants of line 1594 with TYLCV resistance derived from LA1971 remained symptomless after infiltration with both TRV2-180 and TRV2-190 silencing constructs (Table 3). Since all PDS control plants of this line were showing photo bleaching, we assumed that VIGS was working in this line as well. Thus, we expected the majority of the TRV silencing construct-infiltrated plants of the line 1594 to become susceptible if the resistance in this line is conferred by a *Ty*-*1* allelic variant. Two lines derived from *S. chilense* LA2779 were included in the VIGS experiment. In the line Fla. 8680, which carries the *Ty*-*3* allele (Verlaan et al. 2013), all TRV silencing construct-infiltrated plants except for one showed TYLCV symptoms. But resistance was uncompromised in Fla. 8383, which carries the *Ty*-*6* allele (Hutton and Scott 2013), indicating that *Ty*-*6* is different from the *Ty*-*1/Ty*-*3* gene. For each line, at least two VIGS experiments were performed with comparable results.

### Genetic variation of the RDR in tomato and its wild relatives

There is a four amino acid insertion, from positions 12 to 16, present in the *Ty*-*1*/*Ty*-*3* alleles (Verlaan et al. 2013) compared with the MM allele. To determine whether this insertion may be present in a variety of *S. chilense*-derived TYLCV-resistant lines, cDNA was made from six *S. chilense*-derived lines containing *Ty*-*1/Ty*-*3* and two wild *S. chilense* accessions (Figure S4A). Primers were designed to amplify the region of interest, and sequence analysis showed that these four amino acids (Proline, Serine, Cysteine, Isoleucine) are present in all lines. However, there is one synonymous SNP (T-G) among the *S. chilense-*derived lines (Figure S4A).

To check for the presence/absence of the four amino acid insertion among cultivated tomato and wild tomato species, the re-sequenced genome reads of 84 accessions of different species were mapped to the reference genome of *S. lycopersicum* cv. Heinz 1706 and compared for the insertion. In addition, draft de novo assemblies of three tomato wild relatives (*S. arcanum* LA2157, *S. habrochaites* LYC4 and *S. pennellii* LA716) were included in the analysis (Figure S4B). All cultivated tomato lines in the test panel (including *S. lycopersicum* var*. lycopersicum* and *S. lycopersicum* var*. cerasiforme*) and the majority of the wild species do not have the insertion (Figure S4C). Several related wild species in the test panel do, however, have the insertion, i.e. *S. arcanum* LA2157, S*. corneliomulleri* LA118, *S. peruvianum* LA1954, two accessions of *S. huaylasense* (LA1983 & LA1365), *S. habrochaites* LYC4, and *S. pennellii* LA716 (Figure S4B & S4D). Within the 12-bp insertion, one non-synonymous SNP was detected in *S. habrochaites* LYC4, leading to an amino acid change (P → S). In many disease tests (data not shown), *S. arcanum* LA2157 and *S. habrochaites* LYC4 exhibited clear virus symptoms after TYLCV infection.

To further explore crucial *Ty*-*1/Ty*-*3* allele-specific polymorphisms, the sequences of the coding regions of the *RDR* from tomato, nine *S. chilense*-derived lines and five wild tomato accessions were obtained and analysed (Figure S6). Within *S. chilense,* all genotypes have different *RDR* alleles, as shown by the presence of accession-specific SNPs or combination of SNPs. Importantly, five SNPs (two in exon 12, one each in exon 13, 14 and 18, yellow marked in Figure S6) specific to the *Ty*-*1/Ty*-*3* alleles were identified. Additionally, four SNPs (two in exon 2, two in exon 6, green marked in Figure S6) were found to be unique for the *Ty*-*3* allele. The *RDR* cDNA sequence of *S. chilense* G1.1556 contained intron 17, which would result in a premature stop codon. No full-length cDNA sequence was obtained from *S. chilense* LA2779-derived line Fla.8383, but the sequence of exons 12–14 was identical to the MM sequence, indicating the presence of a susceptible *RDR* allele.

**Fig. 2:**
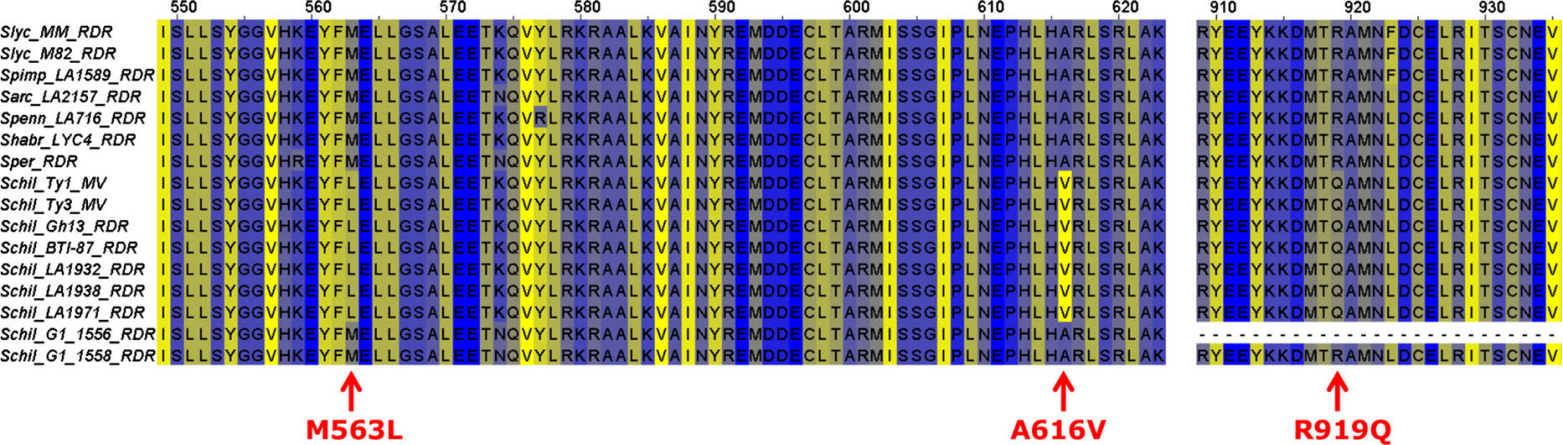
*Ty*-*1/Ty*-3 allele-specific polymorphisms. Partial alignment of protein sequences of the *Ty*-*1* *RDR* alleles; *red arrows* indicate three *Ty*-*1/Ty*-*3*-specific amino acids

**Fig. 3:**
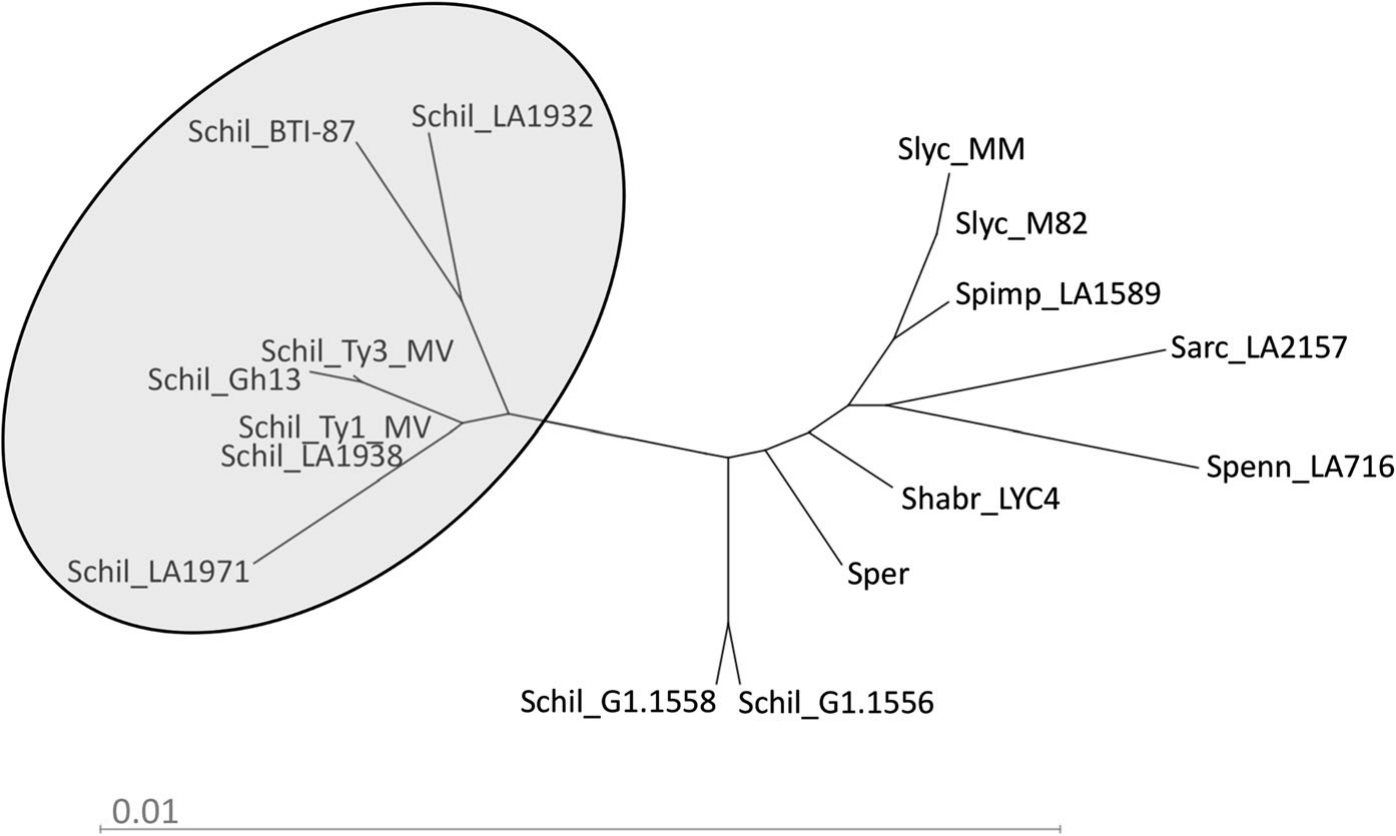
Unrooted phylogenetic tree of protein sequences of *Ty*-*1/Ty*-*3* RDR of accessions of *S. chilens*e and other (wild) tomato species. The RDR proteins of the TYLCV-resistant *S. chilense* accessions cluster in one clade, as indicated

RDR protein sequences were derived from the cDNA sequences and aligned (Figure S7). A small number of *Ty*-*1/Ty*-*3*-specific amino acids were observed, which were shared by TYLCV-resistant *S. chilense* accessions LA1969 (*Ty*-*1*), LA2779 (*Ty*-*3*), LA1932 (*Ty*-*3A*), LA1938, LA1971, and introgression lines BTI-87 and Gh13 reported to contain *Ty*-*3* alleles (Menda et al. 2014; Mejía et al. 2005). These amino acids are L563, V616, and Q919 (numbering based on the *Ty*-*1* allele, SEQ2 in Patent No. WO2012125025). They are absent in *S. chilense* accessions G1.1556 and G1.1558 that do not contain *Ty*-*1* or *Ty*-*3* (Fig. 2). A phylogenetic analysis using an unrooted tree grouped together the proteins of seven *S. chilenseTy*-*1/Ty*-*3* alleles responsible for TYLCV resistance (Fig. 3).

### The catalytic domain of the *Ty*-*1* gene is conserved in tomato and its wild relatives

The catalytic domain of the *Ty*-*1*/*Ty*-*3* allele is characterized by a five amino acid motif, DFDGD (position 723–727) (Verlaan et al. 2013). SNPs in this domain could potentially have an effect on the functioning of this protein. Sequence analysis of an amplified cDNA fragment among all tested *S. chilense-*derived lines showed that there were no SNPs present in the catalytic domain, and furthermore, no differences were found in four amino acids up- or downstream of the catalytic domain (Figure S5). The sequence coding for the catalytic DFDGD motif was also compared among all available sequences used for Figure S4. This region was found to be highly conserved, and no polymorphisms were detected among the susceptible and resistant lines analysed.

### Elevated expression level of the *RDR* alleles in *S. chilense* accessions

Analysis of expression of the *RDR* in *S. chilense*-derived resistant lines revealed significant differences compared with the tomato susceptible allele. The expression level of the alleles was measured at different time points in the presence or absence of the virus (Figure S8). Six resistant lines derived from *S. chilense* LA1969, LA2779, LA1932, LA1938 and LA1971 showed significantly higher relative expression of *RDR* compared with the susceptible allele, despite the presence of the virus. *RDR* transcript levels of line Fla.8383, derived from *S. chilense* LA2779, remained very low, resembling the expression levels of the *ty*-*1* allele from cultivated tomato. This is in agreement with the result that Fla.8383 carries a susceptible *RDR* allele and the TYLCV resistance in this line is conferred by another gene located on chromosome 10 (*Ty*-*6*). Similarly, transcript levels of the accessions *S. arcanum* LA2157, *S. habrochaites* LYC4 and *S. pennellii* LA716 were comparable with those of the susceptible allele.

However, two accessions of *S. chilense* (G1.1556 and G1.1558) with resistance governed by genes of recessive nature (data not shown) also showed a significantly higher level of expression of the *RDR* compared with MM. These results suggest that even though a high expression of the *RDR* is necessary for the *Ty*-*1/Ty*-*3*-mediated resistance, it is not exclusively responsible of the resistant response.

## Discussion

Recently, we cloned the *S. chilense*-derived TYLCV resistance genes *Ty*-*1* and *Ty*-*3* and found that they are allelic and code for an RDR of the DFDGD class (Verlaan et al. 2013). In this study, we show, based on fine-mapping and/or VIGS, that functional *Ty*-*1*/*Ty*-*3*-like alleles are present in *S. chilense* accessions LA1932, LA1938 and likely also in LA1960 and LA1971. We also show that the catalytic domain of the *Ty*-*1* gene is conserved among cultivated tomato and several wild species in the tomato clade. Three *Ty*-*1/Ty*-*3*-specific amino acids were identified among TYLCV-resistant *S. chilense* accessions, each genotype representing different *RDR* alleles. These specific amino acids in concomitance with high gene expression level are indicative of *Ty*-*1/Ty*-*3*-mediated resistance. An insertion of 12 base pairs at the 5-prime part of the coding sequence is, however, found in *S. chilense*-derived alleles and also in several other wild *Solanum* species of which some are known to be susceptible to TYLCV.

### One *S. chilense* accession can harbour more than one TYLCV resistance gene

Many *S. chilense* accessions, including LA1969, LA1932, LA1938, LA2779, LA1960 and LA1971, are resistant to TYLCV. The resistant *Ty*-*1* and *Ty*-*3* alleles were originally identified in LA1969 and LA2779, respectively. The combined data from genetic mapping and VIGS showed the existence of *Ty*-*1/Ty*-*3* allelic variants, which control TYLCV resistance in LA1932 and LA1938. Our mapping results showed that the gene controlling the TYLCV resistance in LA1971 is also located in the same region of *Ty*-*1/Ty*-*3* (Fig. 1); therefore, it was expected that LA1971 harbours a *Ty*-*1/Ty*-*3* allele. However, this hypothesis was not confirmed by the VIGS experiments, since silencing the *Ty*-*1/Ty*-*3* gene did not compromise the resistance in line 1594 derived from LA1971. Expression analysis of the *RDR* in this line showed the highest transcript levels among all the resistant lines tested, about 80 times higher compared with the susceptible *ty*-*1* allele levels (Figure S8). Complete suppression of VIGS-targeted genes in tomato is rarely observed (Sahu et al. 2012); thus, the inability of this silencing approach to repress such high expression levels might have caused the unexpected resistant phenotype. An alternative possibility is that line 1594 may carry, in addition to a *Ty*-*1* allele, another TYLCV resistance gene derived from LA1971. It is worthwhile to note that the LA1971-derived parental line of our mapping population and line 1594 used in the VIGS experiments in this study are different, but “sisters” of the lines described in the previous paper of Pérez de Castro et al. (2013). Checking the LA1971 introgressions in this line, it appeared that line 1594 has multiple introgressions located on chromosomes 6, 7, 10 and 11 (see Fig. 2 in Pérez de Castro et al. 2013). Interestingly, the introgressions on chromosome 10 and 11 overlap with the intervals where *Ty*-*6* derived from *S. chilense* LA2779 (Hutton and Scott 2013) and *Ty*-*2* from *S. habrochaites* B6013 (Yang et al. 2014) are mapped, respectively. Therefore, the presence of a second resistance gene could explain why line 1594, with *S. chilense* LA1971-derived resistance, remained symptomless after silencing of *Ty*-*1*/*Ty*-*3* followed by TYLCV inoculation. Similarly, a resistant response after silencing the *RDR* allele in the *S. chilense* LA2779-derived line Fla.8383 was observed. Sequence analysis revealed that this line does not contain the *Ty*-*1/Ty*-*3*-resistant allele-specific polymorphisms, and transcript levels of the *RDR* in this line resemble those of the susceptible *ty*-*1* allele (Figure S8). As Fla.8383 is devoid of a functional *Ty*-*1/Ty*-*3* allele, the TYLCV resistance in this line is probably conferred by *Ty*-*6*. We are further genotyping these lines to verify our hypothesis. Alternatively, a mapping approach on populations segregating for only one introgression would be helpful in solving the puzzle. Future cloning of *Ty*-*2* and *Ty*-*6* would allow silencing of these two genes in lines 1594 and Fla.8383 to confirm this hypothesis.

The wild tomato species *S. chilense* is self-incompatible and thus heterogeneous, leading to multiple alleles of the same gene present in one accession (Bai et al. 2004). As shown in LA1932, resistant alleles of both *Ty*-*1/Ty*-*3* and *Ty*-*4* are present (Ji et al. 2009). Also in LA2779, both *Ty*-*3* and *Ty*-*6* have been identified. Similarly, LA1971 may carry alleles of *Ty*-*1/Ty*-*3* and other *Ty*-genes, e.g. *Ty*-*6*. Thus, depending on selection procedures and heterogeneity present in *S. chilense*, it is possible that advanced *S. chilense*-derived lines carry different resistance genes for TYLCV resistance. Pyramiding of different *Ty*-genes could possibly provide higher resistance levels and/or broaden the resistance to a wider range of begomoviruses. Therefore, when a species is shown to be resistant to multiple viruses, it is possible—even probable—that more than one gene is contributing to the broad-spectrum resistance. For example, the accession *S. chilense* LA1932 was found to be resistant to *Tomato mottle virus* (ToMoV) and TYLCV (Ji et al. 2009; Scott et al. 1996). It is worthwhile to test whether *Ty*-*1* or *Ty*-*4* confers resistance to both viruses. These genes should be studied more deeply in order to understand their specificity and effectiveness.

It is unfortunate that we did not have enough seeds of an advanced introgression line derived from the accession LA1960 for VIGS. The mapping data showed that a *Ty*-*1* allele is likely present in this accession. However, we cannot rule out the possibility of the presence of other *Ty*-genes in accession LA1960. As shown in Pérez de Castro et al. (2013), the introgression line generated by selecting for TYLCV resistance carries multiple LA1960 fragments, including the *Ty*-*1* region on chromosome 6 and the *Ty*-*6* region on chromosome 10.

### *Ty*-*1/Ty*-*3*-mediated resistance is determined by allele-specific polymorphisms in concomitance with high expression levels of the *RDR*

In our previous study (Verlaan et al. 2013), we detected a 12 base pairs insertion in the 5 prime part of the coding sequence in the resistant *Ty*-*1*/*Ty*-*3* allele and proposed this polymorphism as the most striking difference between the *Ty*-*1*/*Ty*-*3* and *ty*-*1* alleles. Here we show that this 12 base pairs insertion is present in a set of 8 lines/accessions containing different *S. chilense* alleles as well as in the related wild species *S. arcanum*, *S. corneliomulleri*, *S. peruvianum,S. huaylasense*, *S. habrochaites* and *S. pennellii*, evidencing that this feature is not *S. chilense* specific. Since some of these species, e.g. *S. arcanum* LA2157 and *S. habrochaites* LYC4 have exhibited virus symptoms after TYLCV infection (data not shown), we conclude that this insertion cannot be used as a *Ty*-*1/Ty*-*3*-specific marker. By further analysing the *RDR* coding regions, we succeeded in finding five SNPs present in different exons that are specific to the *Ty*-*1/Ty*-*3* allele. These SNPs can be exploited to generate in-gene markers. Further, four SNPs were shown to be unique to *Ty*-*3*, useful for allele-specific marker development. In addition, the origin of the *Ty*-*1/Ty*-*3* alleles can be traced by accession-specific SNPs (Figure S6).

In a previous study, we found that the resistant *Ty*-*1* allele was more highly expressed than the susceptible *ty*-*1* allele (Verlaan et al. 2013). In this study, we observed comparable results where the expression of all *Ty*-*1/Ty*-3-resistant alleles is significantly higher than the susceptible allele. However, the expression level varied among different *RDR* alleles (Figure S8). Surprisingly, elevated expression levels of the *RDR* were also detected *in S. chilense* accessions G1.1556 and G1.1558, which carry a susceptible *ty*-*1* allele. Therefore, we conclude that the expression level of the *RDR* is not solely responsible for the resistance, but this feature together with the *Ty*-*1/Ty*-*3* allele-specific amino acid sequence determines the resistance response.

The same set of tomato accessions/lines was also used to compare the typical DFDGD catalytic domain of the RDRγ type to which the *Ty*-*1* gene belongs. No SNPs were found in the domain nor in 12 base pairs up- or downstream of this domain. Further, no differences were found among a *Ty*-*2* carrying line, a wild *S. pimpinellifolium* and the same nine *S. chilense*-derived lines described before. The region was also compared among the same set of cultivated lines and wild tomato accessions. It was found that the catalytic domain was conserved, and no SNPs were found in any of the accessions/lines tested. This could indicate that this gene is important for the plant and that SNPs in the catalytic domain have a negative effect on plant fitness.

## Conclusion

In conclusion, this study shows that probably many *S. chilense* accessions carry a TYLCV resistance locus on chromosome 6, allelic to *Ty*-*1*/*Ty*-*3*. Fine-mapping and/or more VIGS experiments could prove whether this is really true. The catalytic domain of the *Ty*-*1*/*Ty*-*3* gene is conserved among *Solanum* species. The 12 base pair insertion in *Ty*-*1*/*Ty*-*3* is present in *S. chilense* and in six other wild *Solanum* species, and not exclusively linked to TYLCV resistance. To develop allele-specific markers, SNPs unique to the resistant *Ty*-*1/Ty*-*3* alleles can be used. Moreover, our study shows that (1) VIGS can be applied as a tool for testing allelism, and (2) more than one TYLCV resistance gene can be present in one *S. chilense* accession.

## Materials and methods

### Plant materials

In Spain, four breeding lines (two derived from *S. chilense* LA1932, one from *S. chilense* LA1960, and one from *S. chilense* LA1971) were selected based on the absence of TYLCD symptoms, fertility and phenotypical similarity with the cultivated tomato (Figure S2). These lines were used to generate different F_2_ populations (Table 1) (Pérez de Castro et al. 2013). In addition, for the line derived from accession LA1971, respective backcrosses to both parents were generated (Table 1). These F_2_ populations and backcross populations were used in this study for genetic mapping. Two of the breeding lines were used for the VIGS experiment: 1538, derived from *S. chilense* LA1932 and corresponding to line 2 in Pérez de Castro et al. (2013) and 1594, derived from *S. chilense* LA1971 and corresponding to line 5 in Pérez de Castro et al. (2013). Moreover, the line B26, progeny of one homozygous resistant F_2_ plant (recombinant no. 19 in Table S1) derived from LA1932, was also included in the VIGS experiment (Table 3).

In Florida, four breeding lines with begomovirus resistance derived from different *S. chilense* sources were developed through the University of Florida tomato breeding programme. Resistance to either TYLCV and/or tomato mottle virus was selected phenotypically over multiple seasons. Fla. 8680 (Verlaan et al. 2013) and Fla. 8383 both have resistance derived from accession LA2779. Fla. 8783 is a small-fruited line with resistance from accession LA1932. And Fla. 976 has resistance derived from LA1938. Resistance in each line, with exception of Fla. 8383, was determined previously to co-segregate with a *S. chilense* introgression on chromosome 6 and spanning the *Ty*-*1/Ty*-*3* locus.

### TYLCV inoculation

For TYLCV tests in Wageningen, The Netherlands, an infectious TYLCV-IL clone (pTYCz40a) originating from Israel was used for agro-inoculation using the method as described in Verlaan et al. (2011). Briefly, *A. tumefaciens* LBA4404 was transformed, cultured in LB, pelleted, and resuspended in infiltration medium at an OD_600_ of 0.5. Seeds were sown, and plants were kept under greenhouse conditions at a temperature of 23 °C and relative humidity of 60 % during a 16-h day/8-h night regime. Three-week-old seedlings were infiltrated by pressure inoculation in the leaves with a needleless syringe. For the VIGS experiments, the agro-infiltration was done 2 weeks after TRV inoculation.

For TYLCV test in Valencia, Spain, whitefly-mediated inoculation as previously described by Pérez de Castro et al. (2013) was used. Whiteflies were biotype Q (supplied by F. Beitia, Instituto Valenciano de Investigaciones Agrarias, IVIA, Valencia, Spain) and viruliferous for the Spanish TYLCV isolate TYLCV-Mld[ES:72:97] (accession no. AF071228). This “Mild” isolate also originates from Israel (Fauquet et al. 2008). Both the TYLCV-IL and the TYLCV-Mld[ES:72:97] isolates belong to the same TYLCV viral species. Briefly, plants were inoculated at 3–4 true-leaf stage during 7 days in a climatic chamber inside muslin-covered cages. After this period, plants were transplanted in a greenhouse with controlled temperature until the end of the assay. Symptom severity was scored at 15, 25, 35, 45 and 55 days post inoculation using a scale (Friedmann et al. 1998) from 0 (no visible symptoms) to 4 (very severe symptoms; plants cease to grow) (Figure S3). The limit to classify individual plants as resistant or susceptible was established at symptom score 2, based on previous studies (Pérez de Castro et al. 2007). Plants scored under 2 were considered resistant, given that no significant yield losses were expected as a consequence of infection, while plants scored 2 or higher were considered susceptible.

### TRV-based VIGS

For the silencing experiments, TRV constructs and procedures as described in (Verlaan et al. 2013) were used. Briefly, *A. tumefaciens* strain GV3101 containing the TRV replicons were cultured, pelleted, and resuspended in infiltration medium. Agro-infiltration was performed on cotyledons of 10-day-old seedlings using pressure inoculation.

### RNA isolation and cDNA synthesis

For sequence analysis, 3-week-old seedlings were agro-inoculated as described above. Three weeks after agro-inoculation, top leaves of plants were harvested and grinded in liquid nitrogen using mortar and pestle. Total RNA was extracted by using the RNeasy Plant Mini Kit (Qiagen) as described by the manufacturer. One-µg RNA was digested using DNase I (Amp. Grade) following the manufacturers protocol (Invitrogen), and cDNA was synthesized using the iScript cDNA Synthesis Kit following the protocol (Bio-Rad).

### Sequence analysis of the *S. chilense*-derived lines and accessions

For amplifying the region containing the 5 prime deletion, primers Del-F1 (5′-TTCAAGTATATACAGGAAAAATGGGTGATCCG-3′) and Del-R1 (5′-CTGAGGGCTTGCACAGGCCAAT-3′) were used. For amplifying the region containing the catalytic domain, primers DFDGD-F4 (5′-GGGCGTGTTTTGGTCTACAG-3′) and DFDG-R4 (5′-GCTATCAGCTGCCAGAGACAT-3′) were used. PCR amplification was performed according to standard protocols in an Eppendorf Mastercycler Pro. Amplified fragments were sequenced and analysed using SeqMan Pro 9 (DNA Star). Alignments were made with MEGA version 5 (Tamura et al. 2011).

The *RDR* cDNA sequence from *S. lycopersicum* “Moneymaker” (MM), the *Ty*-*1* allele from *S. chilense* LA1969 and the *Ty*-*3* allele from *S. chilense* LA2779 were described by Verlaan et al. (2013) and published in Patent No. WO2012125025 & US2014208459 (SEQ1 = *Ty*-*1*; SEQ3 = MM). The *RDR* cDNA sequence from *S. peruvianum* was obtained from the SGN *S. peruvianum* de novo transcriptome (a19742). Genomic sequences/contigs were available for *Solanum lycopersicum* M82, *S. pimpinellifolium* LA1589, *S. arcanum* LA2157, *S. pennellii* LA716, *S. habrochaites* LYC4 (NCBI WGS whole genome shotgun contigs data), *S. chilense* introgression line Gh13 and *S. chilense* introgression line BTI-87 (SGN database tomato inbred lines). *RDR* exons were extracted from the genomic sequence based on homology with the *S. lycopersicum* “Moneymaker ” *RDR* allele. In the *S. habrochaites* LYC4 genomic sequence, part of the *RDR* gene was missing, i.e. a large part of intron 8 and exon 9. This was confirmed by PCR analysis and sequencing. RNA was isolated from TYLCV-infected *S. chilense* LA1932, LA1938, LA1971, G1.1556 and G1.1558, 19 days post-infection. cDNA was prepared, and full-length *RDR* cDNA sequences were obtained by PCR with primers Ty-F7 and Ty-R5 (Verlaan et al. 2013). Sequences were determined from nested PCR products with primers RDR-F3 + R10, RDR-F7 + R7, RDR-F6 + R4, RDR-F4 + R5 (Figure S4 in Verlaan et al. 2013).

The RDR cDNA sequence from *S. chilense* G1.1556 was smaller than the expected size of approximately 3 kb. Nested PCR with primers RDR-F7 and RDR-R7 was not successful. Therefore, exons 9–14 were amplified from genomic DNA, although we could not verify whether they are included in the transcript. The PCR product with primers RDR-F3 and RDR-R10 was larger than the expected 1068 bp and proved to contain intron 17.

### De novo assembled wild species genomes and re-sequencing collection and analysis of the deletion and the catalytic domain

Data of the 84 accessions of the 100 tomato genome re-sequencing consortium (The 100 Tomato Genome Sequencing Consortium 2014) were obtained from the European Nucleotide Archive (http://www.ebi.ac.uk/ena/; project PRJEB5235). The de novo assemblies of *S. arcanum* LA2157, *S. habrochaites* LYC4, and *S. pennellii* LA716 were obtained from the same resource and are available under the project numbers PRJEB5226, PRJEB52267 and PRJEB52268, respectively. In short, 84 tomato and related wild species were re-sequenced with a read depth of approximately 42× (The 100 Tomato Genome Sequencing Consortium 2014). For a list of sequenced species and their variants, we refer to http://www.tomatogenome.net. Sequence reads were mapped to the reference genome of *S. lycopersicon* cv. Heinz version SL2.40 (The Tomato Genome Consortium 2012) using BWA (Li and Durbin 2009), SNP and INDELS were called using samtools (Li et al. 2009) and saved in the variant call format (VCF) (Danecek et al. 2011). Variants were visually inspected using the Integrative Genomics Viewer (IGV) (Robinson et al. 2011).

### Phylogenetic analysis

An unrooted neighbour joining tree was constructed from multiple sequence alignment using MAFFT version 7 (http://mafft.cbrc.jp/alignment/server/).

### Quantitative RT-PCR

For the gene expression experiment, leaf samples of the top part of each plant were taken 0 and 19 days after TYLCV inoculation; the mock treatment consisted of infiltration media without bacteria. Total RNA extraction, cDNA synthesis and quantitative real-time PCR were performed as described in Verlaan et al. (2013). For RT-PCR of *Ty*-*1/ty*-*1*, primers 180-F1 and 180-R2 were used. The actin (ACT) gene was used as reference, using primers ACT-F and ACT-R; gene expression levels were calculated using the **ΔΔ**Ct method (Verlaan et al. 2013).

## Electronic supplementary material

Figure S1 Physical chromosome locations of mapped tomato genes conferring resistance to TYLCV. Schematic representation of chromosome location of *Ty-1, Ty-3, Ty3-A* (incompletely dominant, Verlaan et al. 2013, Ji et al. 2007, Scott et al. 1996), *Ty-2* (dominant, Yang et al. 2014), *Ty-4* (incompletely dominant, Ji *et al.* 2009), *ty-5* (recessive, Anbinder et al. 2009) and *Ty-6* (Hutton and Scott 2013). Source of *ty-5* is tomato breeding line TY172, derived from 4 different accessions of *Solanum peruvianum*. Grey-shaded regions represent pericentromeric heterochromatin; approximate physical positions are shown on the left side of chromosomes and represent millions of basepairs (TIFF 93 kb)

Figure S2 Development of resistant parental lines derived from a cross between the tomato breeding line Fortuna C (Fc, susceptible) and *Solanum chilense* accessions LA1932 (a), LA1960 (b) and LA1971 (c) (Picó et al. 1999). BC: backcross generation with selection for resistance; S: selfing generation with selection for resistance (TIFF 60 kb)

Figure S3 Representative pictures of disease scores. Symptom severity scale (Friedmann et al. 1998) from 0 (no symptoms) to 4 (severe symptoms) (TIFF 889 kb)

Figure S4 Alignment of sequences of the region containing the 5-prime insertion in the *Ty-1* allele. All *Solanum chilense*-derived lines have the 12 base pair insertion (A). There is one non-synonymous SNP in *S. arcanum* LA2157 and *S. habrochaites* LYC4 (B). Of the multiple species tested, six had the insertion, e.g. *S. arcanum,* *S. corneliomulleri*, *S. peruvianum*, *S. huaylasense*, *S. habrochaites* and *S. pennellii* (B, C and D). Sequences from (A) have been obtained from cDNA, sequences from (B) have been obtained from a *de novo* assembly of these three accessions (C) and (D) have been obtained from whole genome re-sequencing. Note: Read-mapping information of *S. habrochaites* and *S. pennellii* against Heinz was ambiguous, and thus, cautions need to be taken for using data of these two species (TIFF 176 kb)

Figure S5 Alignment of cDNA sequences of the region containing the catalytic domain of the *RDR*. All *S. chilense*-derived lines have an identical sequence in this region. Accessions from 14 *Solanum* species also have the same sequence. All species in the full genome data set were also analysed, but no SNPs were observed (TIFF 296 kb)

Figure S6 Alignment of full-length cDNA sequences of the *Ty-1/Ty-3* *RDR*. Sequences of two *S. lycopersicum* lines, nine *S. chilense*-derived lines/accessions and five related *Solanum* accessions were obtained and compared to explore for allele-specific polymorphisms. Start positions of the exons are indicated. The 5’ indel and the catalytic domain are indicated and highlighted in red. The premature stop codon in *S. pennellii* LA716 is highlighted in red. Five *Ty-1/Ty-3*-specific SNPs are highlighted in yellow; four *Ty-3*-specific SNPs are highlighted in green (PDF 255 kb)

Figure S7 Alignment of protein sequences of the *Ty-1/Ty-3* *RDR*. Protein sequences are derived from cDNA sequences of accessions and derived lines as in Figure S6. The 5’ indel and the catalytic domain are highlighted in red. A premature stop codon in *S. pennellii* LA716 is highlighted in red. Three *Ty-1/Ty-3*-specific amino acids are highlighted in yellow; two *Ty-3*-specific amino acids are highlighted in green (PDF 111 kb)

Figure S8 Relative expression of the *Ty-1/Ty-3* *RDR* in different accessions of *S. chilense*. Normalized fold gene expression of the target gene in derived introgression lines or *Solanum* accessions as determined by qRT-PCR; *S. chilense* LA1969, LA1932, LA1938, LA1971, LA2779, G1.1556, G1.1558 and related species *S. arcanum* LA2157, *S. habrochaites* LYC4 and *S. pennellii* LA716 are also included in the analysis. Time points T0 and T1 (0 and 19 days after *TYLCV* or *mock* inoculation, respectively) and genotypes (Moneymaker (MM) vs. each *RDR* allele source) are shown on the *x*-axis. Values are normalized against the Moneymaker T0 sample; *bars* represent means and standard error of four biological replicas. *Asterisks* above the *bars* represent significant differences between genotypes per time point and *mock* or *TYLCV* treatment (*P < 0.05, **P < 0.01, *** *P* < 0.001) (TIFF 304 kb)

Table S1 Recombinants identified in the region between markers C2_At3g46780 and M-M005H10 on chromosome 6 (TIFF 632 kb)

Table S2 Markers used for mapping studies (TIFF 154 kb)
