# Supplementary figures and images for: Assessing the genetic variation of Ty-1 and Ty-3 alleles conferring resistance to tomato yellow leaf curl virus in a broad tomato germplasm
Source: Mol Breed. 2015 May 26;35(6):132. doi: 10.1007/s11032-015-0329-y (PMC4442973; doi:10.1007/s11032-015-0329-y)

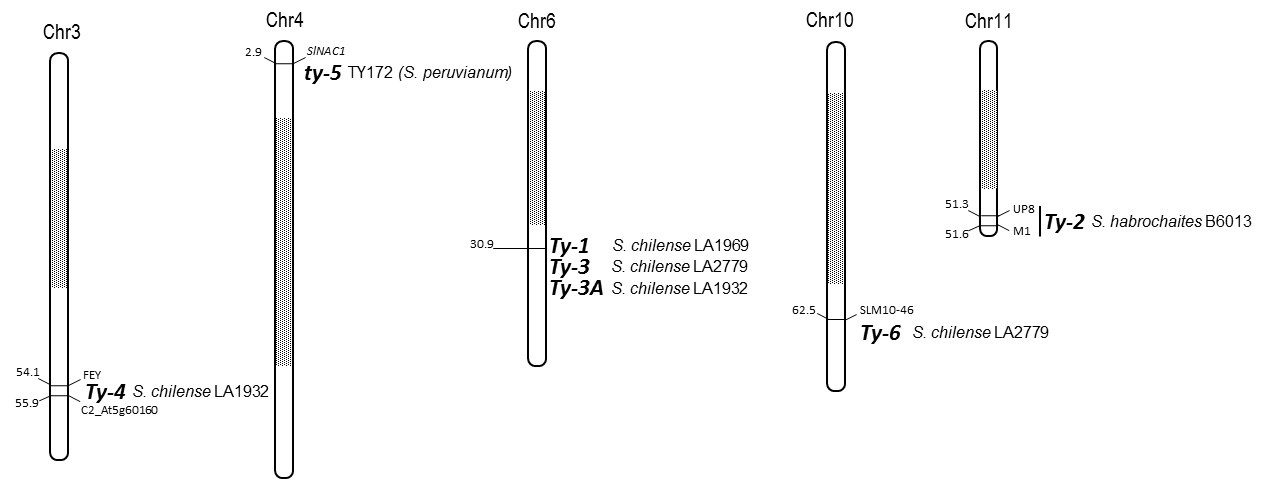

Supplement: Supplementary file 1 — Physical chromosome locations of mapped tomato genes conferring resistance to TYLCV. Schematic representation of chromosome location of Ty-1, Ty-3, Ty3-A (incompletely dominant, Verlaan et al. 2013, Ji et al. 2007, Scott et al. 1996), Ty-2 (dominant, Yang et al. 2014), Ty-4 (incompletely dominant, Ji et al. 2009), ty-5 (recessive, Anbinder et al. 2009) and Ty-6 (Hutton and Scott 2013). Source of ty-5 is tomato breeding line TY172, derived from 4 different accessions of Solanum peruvianum. Grey-shaded regions represent pericentromeric heterochromatin; approximate physical positions are shown on the left side of chromosomes and represent millions of basepairs (TIFF 93 kb) [file 11032_2015_329_MOESM1_ESM.tif]

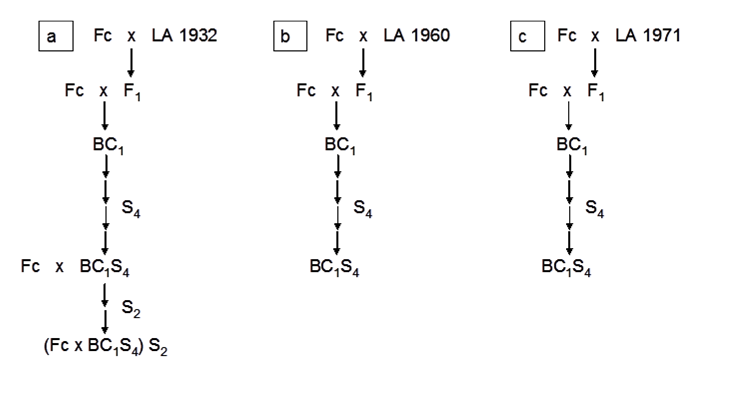

Supplement: Supplementary file 2 — Development of resistant parental lines derived from a cross between the tomato breeding line Fortuna C (Fc, susceptible) and Solanum chilense accessions LA1932 (a), LA1960 (b) and LA1971 (c) (Picó et al. 1999). BC: backcross generation with selection for resistance; S: selfing generation with selection for resistance (TIFF 60 kb) [file 11032_2015_329_MOESM2_ESM.tif]

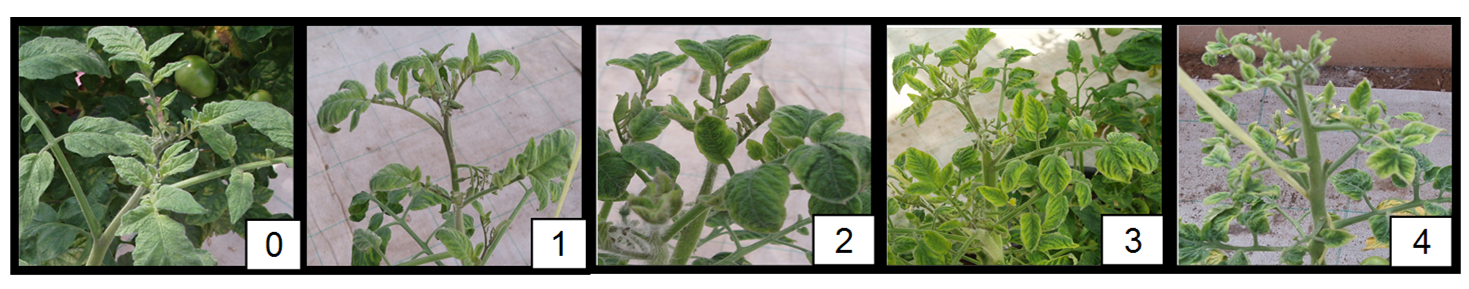

Supplement: Supplementary file 3 — Representative pictures of disease scores. Symptom severity scale (Friedmann et al. 1998) from 0 (no symptoms) to 4 (severe symptoms) (TIFF 889 kb) [file 11032_2015_329_MOESM3_ESM.tif]

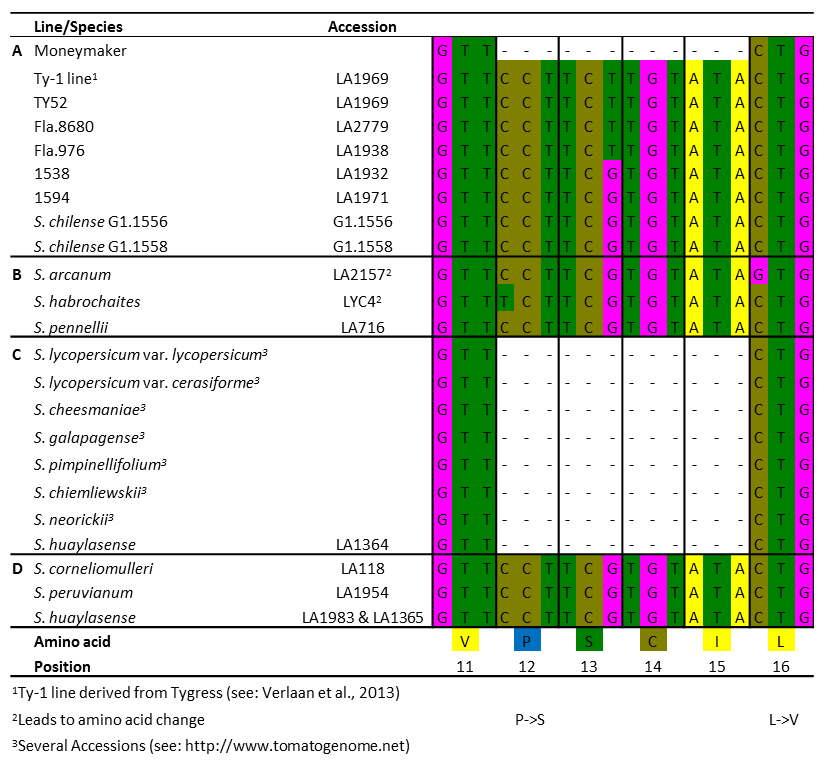

Supplement: Supplementary file 4 — Alignment of sequences of the region containing the 5-prime insertion in the Ty-1 allele. All Solanum chilense-derived lines have the 12 base pair insertion (A). There is one non-synonymous SNP in S. arcanum LA2157 and S. habrochaites LYC4 (B). Of the multiple species tested, six had the insertion, e.g. S. arcanum, S. corneliomulleri, S. peruvianum, S. huaylasense, S. habrochaites and S. pennellii (B, C and D). Sequences from (A) have been obtained from cDNA, sequences from (B) have been obtained from a de novo assembly of these three accessions (C) and (D) have been obtained from whole genome re-sequencing. Note: Read-mapping information of S. habrochaites and S. pennellii against Heinz was ambiguous, and thus, cautions need to be taken for using data of these two species (TIFF 176 kb) [file 11032_2015_329_MOESM4_ESM.tif]

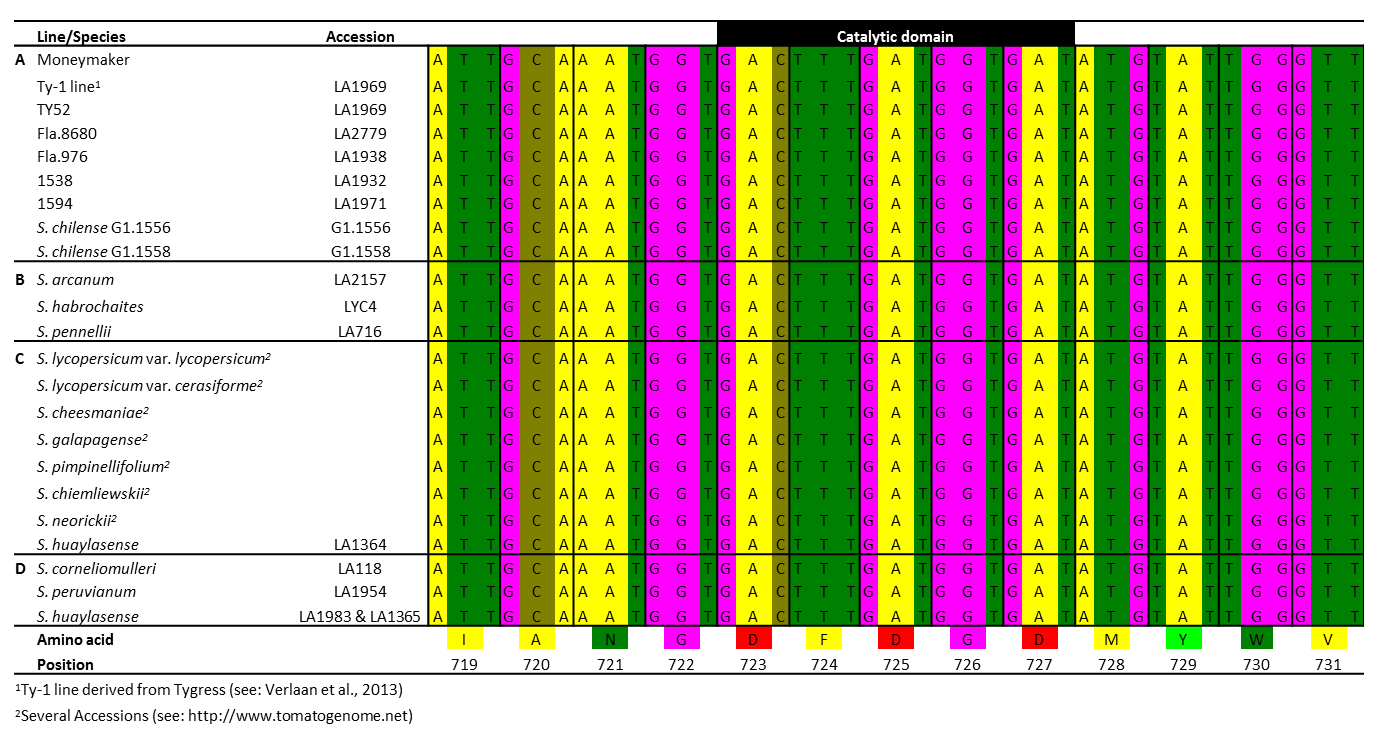

Supplement: Supplementary file 5 — Alignment of cDNA sequences of the region containing the catalytic domain of the RDR. All S. chilense-derived lines have an identical sequence in this region. Accessions from 14 Solanum species also have the same sequence. All species in the full genome data set were also analysed, but no SNPs were observed (TIFF 296 kb) [file 11032_2015_329_MOESM5_ESM.tif]

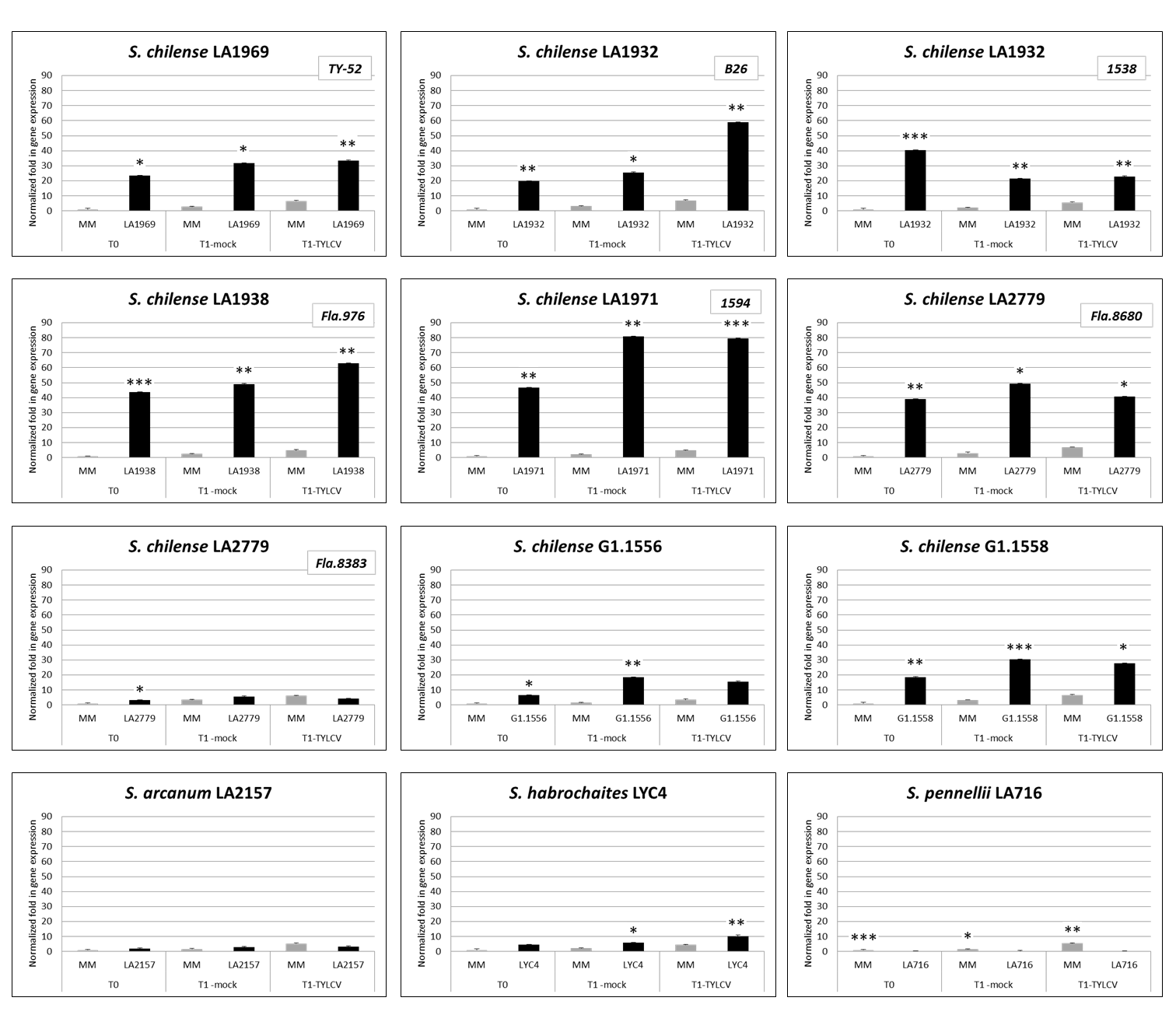

Supplement: Supplementary file 8 — Relative expression of the Ty-1/Ty-3 RDR in different accessions of S. chilense. Normalized fold gene expression of the target gene in derived introgression lines or Solanum accessions as determined by qRT-PCR; S. chilense LA1969, LA1932, LA1938, LA1971, LA2779, G1.1556, G1.1558 and related species S. arcanum LA2157, S. habrochaites LYC4 and S. pennellii LA716 are also included in the analysis. Time points T0 and T1 (0 and 19 days after TYLCV or mock inoculation, respectively) and genotypes (Moneymaker (MM) vs. each RDR allele source) are shown on the x-axis. Values are normalized against the Moneymaker T0 sample; bars represent means and standard error of four biological replicas. Asterisks above the bars represent significant differences between genotypes per time point and mock or TYLCV treatment (*P < 0.05, **P < 0.01, *** P < 0.001) (TIFF 304 kb) [file 11032_2015_329_MOESM8_ESM.tif]

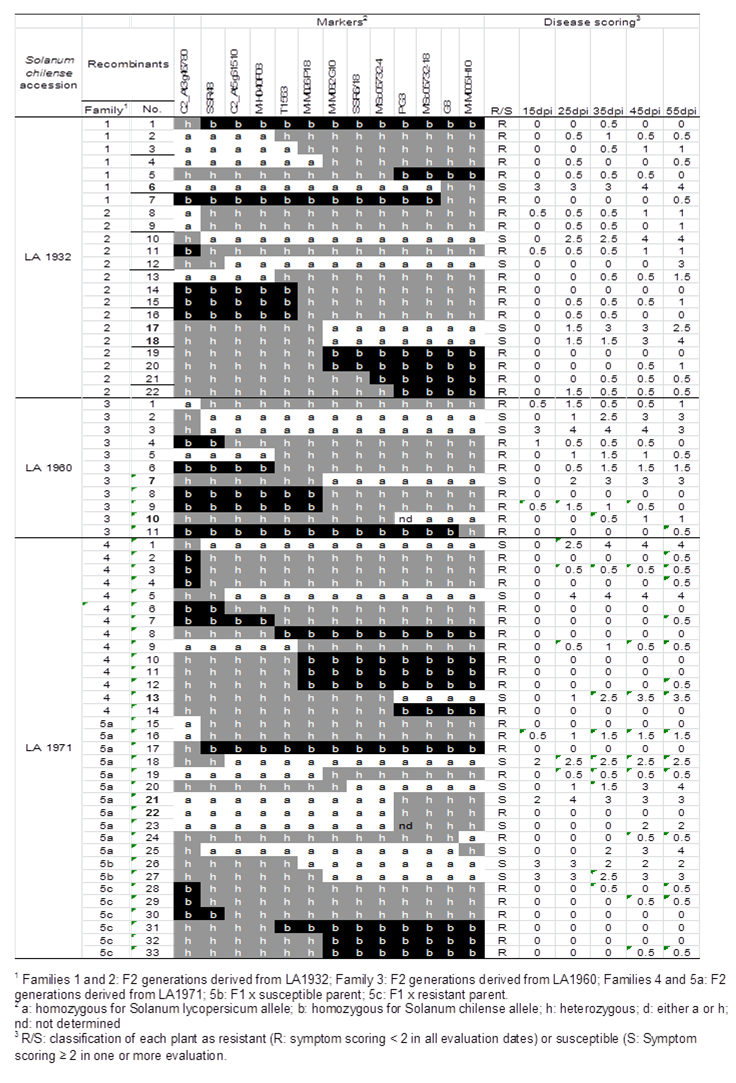

Supplement: Supplementary file 9 — Recombinants identified in the region between markers C2_At3g46780 and M-M005H10 on chromosome 6 (TIFF 632 kb) [file 11032_2015_329_MOESM9_ESM.tif]

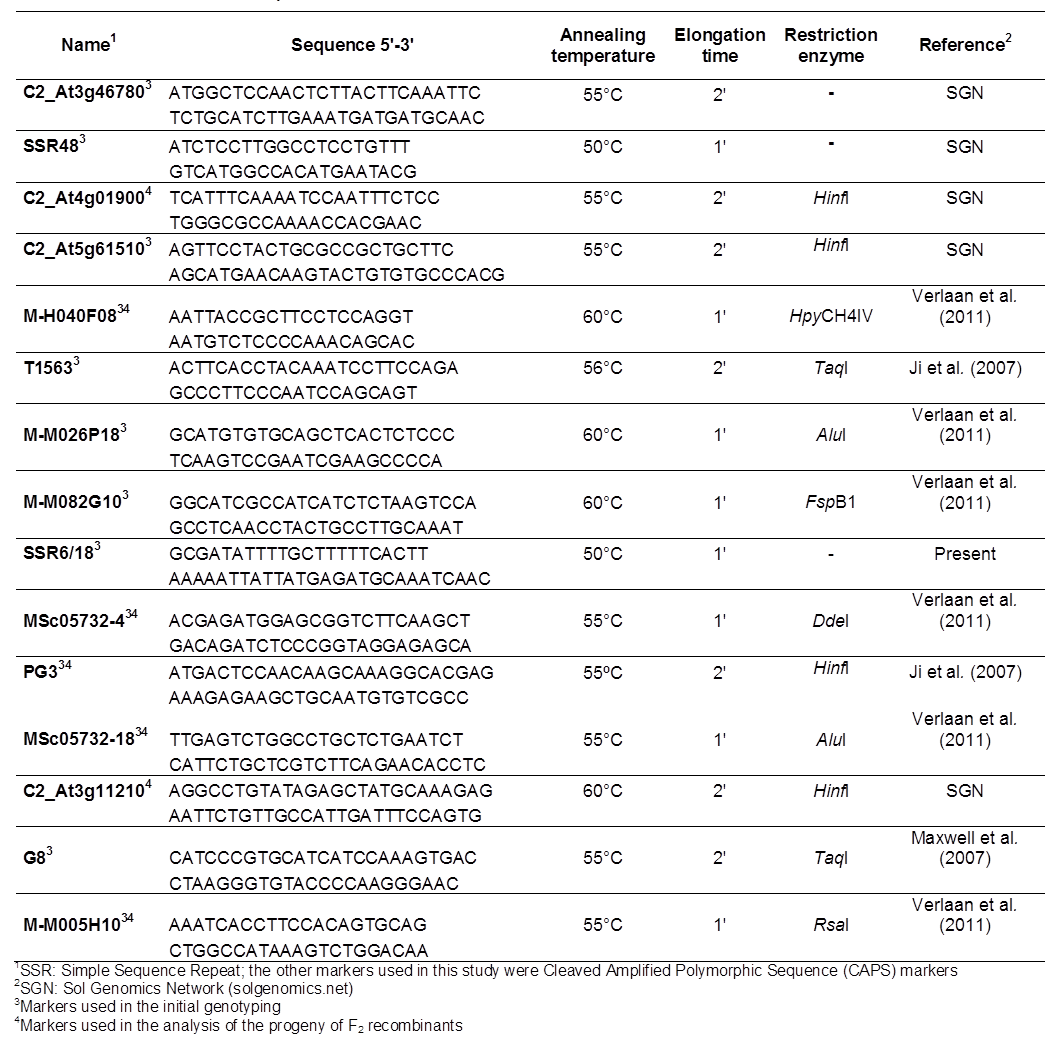

Supplement: Supplementary file 10 — Markers used for mapping studies (TIFF 154 kb) [file 11032_2015_329_MOESM10_ESM.tif]
