# Supplementary material for: Assessing the genetic variation of Ty-1 and Ty-3 alleles conferring resistance to tomato yellow leaf curl virus in a broad tomato germplasm
Source: Mol Breed. 2015 May 26;35(6):132. doi: 10.1007/s11032-015-0329-y (PMC4442973; doi:10.1007/s11032-015-0329-y)
Supplement: Supplementary file 6 — Alignment of full-length cDNA sequences of the Ty-1/Ty-3 RDR. Sequences of two S. lycopersicum lines, nine S. chilense-derived lines/accessions and five related Solanum accessions were obtained and compared to explore for allele-specific polymorphisms. Start positions of the exons are indicated. The 5’ indel and the catalytic domain are indicated and highlighted in red. The premature stop codon in S. pennellii LA716 is highlighted in red. Five Ty-1/Ty-3-specific SNPs are highlighted in yellow; four Ty-3-specific SNPs are highlighted in green (PDF 255 kb) [file 11032_2015_329_MOESM6_ESM.pdf]

Figure S6 RDR cDNA alignment

exon 1

```
XM_010323869        ATGGGTGATCCGTTGATTGAAGAAATTGATGTT------------CTGGATGCACCTTTACCATATTCTGTAGAGACGATGCTTGATAGAATCTGCAAGG   88
Slyc_MM_RDR         ATGGGTGATCCGTTGATTGAAGAAATTGATGTT------------CTGGATGCACCTTTACCATATTCTGTAGAGACGATGCTTGATAGAATCTGCAAGG   88
Slyc_M82_RDR        ATGGGTGATCCGTTGATTGAAGAAATTGATGTT------------CTGGATGCACCTTTACCATATTCTGTAGAGACGATGCTTGATAGAATCTGCAAGG   88
Spimp_LA1589_RDR    ATGGGTGATCCGTTGATTGAAGAAATTGATGTT------------CTGGATGCACCTTTACCATATTCTGTAGAGACGATGCTTGATAGAATCTGCAAGG   88
Sarc_LA2157_RDR     ATGGGTGATCCGTTGATTGAAGAAATTGATGTTCCTTCGTGTATAGTGGATGCACCTTTACCATATTCTGTAGAGACGATGCTTGATAGAATCTGCAAGG  100
Spenn_LA716_RDR     ATGGGTGACCCATTTATTGAAGAAATTGATGTTCCTTCGTGTATACTGGATGCACCTTTACCATATTCTGTAGAGACGATGCTTGAGAGAATCTGCAAGG  100
Shabr_LYC4_RDR      ATGGGTGACCCATTTGTTGAAGAAATTGATGTTTCTTCGTGTATACTGGATGCACCTTTACCATATTCTGTAGAGACGATGCTTGAGAGAATCTGCAAGG  100
Sper_RDR            ATGGGTGATCCGTTGATTGAAGAAATGGATGTTCCTTCGTGTATACTGGATGCACCTTTACCATATTCTGTAGAGACGATGCTTGATAGAATCTGCAAGG  100
Schil_LA1969_Ty1    ATGGGTGATCCGTTGATTGAAGAAATTGATGTTCCTTCTTGTATACTGGATGCACCTTTACCATATTCTGTAGAGACGATGCTTGATAGAATCTGCAAGG  100
Schil_LA2779_Ty3    ATGGGTGATCCGTTGATTGAAGAAATTGATGTTCCTTCTTGTATACTGGATGCACCTTTACCATATTCTGTAGAGACGATGCTTGATAGAATCTGCAAGG  100
Schil_Gh13_RDR      ----------------------------------------------------------------------------------------------------
Schil_BTI-87_RDR    ATGGGTGATCCGTTGATTGAAGAAATTGATGTT------------CTGGATGCACCTTTACCATATTCTGTAGAGACGATGCTTGATAGAATCTGCAAGG   88
Schil_LA1932_RDR    ATGGGTGATCCGTTGATTGAAGAAATTGATGTTCCTTCGTGTATACTGGATGCACCTTTACCATATTCTGTAGAGACGATGCTTGATAGAATCTGCAAGG  100
Schil_LA1938_RDR    ATGGGTGATCCGTTGATTGAAGAAATTGATGTTCCTTCTTGTATACTGGATGCACCTTTACCATATTCTGTAGAGACGATGCTTGATAGAATCTGCAAGG  100
Schil_LA1971_RDR    ATGGGTGATCCGTTGATTGAAGAAATTGATGTTCCTTCGTGTATACTGGATGCACCTTTACCATATTCTGTAGAGACGATGCTTGATAGAATCTGCAAGG  100
Schil_G1_1556_RDR   ATGGGTGATCCGTTGATTGAAGAAATKGATGTTCCTTCGTGTATACTGGATGCRCCTTTACCATATTCTGTAGAGACGATGCTTGATAGAATCTGCAAGG  100
Schil_G1_1558_RDR   ATGGGTGATCCGTTGATTGAAGAAATTGATGTTCCTTCGTGTATACTGGATGCACCTTTACCATATTCTGTAGAGACGATGCTTGATAGAATCTGCAAGG  100
```

indel

```
XM_010323869        AGCAGGGGCAAAAACCACCGTGTACTGGCATTAGAAGGAGGCTGAGCTCTATTGGTGAAAAAGGGTCATTAGAAATGCTCAAAATAATATCACGTCGTCC  188
Slyc_MM_RDR         AGCAGGGGCAAAAACCACCGTGTACTGGCATTAGAAGGAGGCTGAGCTCTATTGGTGAAAAAGGGTCATTAGAAATGCTCAAAATAATATCACGTCGTCC  188
Slyc_M82_RDR        AGCAGGGGCAAAAACCACCGTGTACTGGCATTAGAAGGAGGCTGAGCTCTATTGGTGAAAAAGGGTCATTAGAAATGCTCAAAATAATATCACGTCGTCC  188
Spimp_LA1589_RDR    AGCAGGGGCAAAAACCACCGTGTACTGGCATTAGAAGGAGGCTGAGCTCTATTGGTGAAAAAGGGTCATTAGAAATGCTCAAAATAATATCACGTCGTCC  188
Sarc_LA2157_RDR     AGCAGGGGCAAAAACCACCGTGTACTGGCATTAGAAGGAGGCTGAGCTCTATTGGTGAAAAAGGGTCATTAGAAATGCTCAAAATAATATCACGTCGTCC  200
Spenn_LA716_RDR     AGCAGGGGCAGAAACCACCGTGTACTGGCATTAGAAGGAGGCTGAGCTCTATTGGTGAAAAAGGGTCATTAGAAATGCTCAAAATAATATCACGTCGTCC  200
Shabr_LYC4_RDR      AGCAGGGGCAAAAACCACCGTGTACTGGCATTAGAAGGAGGCTGAGCTCTATTGGTGAAAAAGGGTCATTAGAAATGCTCAAAATAATATCACGTCGCCC  200
Sper_RDR            AGCAGGGGCAAAAACCACCGTGTACTGGCATTAGGAGGAGGCTGAGCTCTATTGGTGAAAAAGGGTCATTAGAAATGCTCAAAATAATATCACGTCGTCC  200
Schil_LA1969_Ty1    AGCAGGGGCAAAAACCACCGTGTACTGGCATTAGAAGGAGGCTGAGCTCTATTGGTGAAAAAGGGTCATTAGAAATGCTCAAAATAATATCACGTCGTCC  200
Schil_LA2779_Ty3    AGCAGGGGCAAAAACCACCGTGTACTGGCATTAGAAGGAGGCTGAGCTCTATTGGTGAAAAAGGGTCATTAGAAATGCTCAAAATAATATCACGTCGTCC  200
Schil_Gh13_RDR      -------------ACCACCGTGTACTGGCATTAGAAGGAGGCTGAGCTCTATTGGTGAAAAAGGGTCATTAGAAATGCTCAAAATAATATCACGTCGTCC   87
Schil_BTI-87_RDR    AGCAGGGGCAAAAACCACCGTGTACTCGCATTAGAAGGAGGCTGAGCTCTATTGGTGAAAAAGGGTCATTAGAAATGCTCAAAATAATATCACGTCGTCC  188
Schil_LA1932_RDR    AGCAGGGGCAAAAACCACCGTGTACTGGCATTAGAAGGAGGCTGAGCTCTATTGGTGAAAAAGGGTCATTAGAAATGCTCAAAATAATATCACGTCGTCC  200
Schil_LA1938_RDR    AGCAGGGGCAAAAACCACCGTGTACTGGCATTAGAAGGAGGCTGAGCTCTATTGGTGAAAAAGGGTCATTAGAAATGCTCAAAATAATATCACGTCGTCC  200
Schil_LA1971_RDR    AGCAGGGGCAAAAACCACCGTGTACTGGCATTAGAAGGAGGCTGAGCTCTATTGGTGAAAAAGGGTCATTAGAAATGCTCAAAATAATATCACGTCGTCC  200
Schil_G1_1556_RDR   AGCAGGGGCAAAAACCACCGTGTACTGGCATTAGAAGGAGGCTGAGCTCTATTGGTGAAAAAGGGTCATTAGAAATGCTCAAAATAATATCACGTCGTCC  200
Schil_G1_1558_RDR   AGCAGGGGCAAAAACCACCGTGTACTGGCATTAGAAGGAGGCTGAGCTCTATTGGTGAAAAAGGGTCATTAGAAATGCTCAAAATAATATCACGTCGTCC  200

XM_010323869        TATCAAGAAGAGTCTCTCTGCTTTTCTTGTTTACATGATTGATCGCTACCCGGATTGTCTCTCCTCTTCCTCTAGCCCCTTCAATTGTCTACTCAAACGC  288
Slyc_MM_RDR         TATCAAGAAGAGTCTCTCTGCTTTTCTTGTTTACATGATTGATCGCTACCCGGATTGTCTCTCCTCTTCCTCTAGCCCCTTCAATTGTCTACTCAAACGC  288
Slyc_M82_RDR        TATCAAGAAGAGTCTCTCTGCTTTTCTTGTTTACATGATTGATCGCTACCCGGATTGTCTCTCCTCTTCCTCTAGCCCCTTCAATTGTCTACTCAAACGC  288
Spimp_LA1589_RDR    TATCAAGAAGAGTCTCTCTGCTTTTCTTGTTTACATGATTGATCGCTACCCGGATTGTCTCTCCTCTTCCTCTAGCCCCTTCAATAGTCTACTCAAACGC  288
Sarc_LA2157_RDR     TATCAAGAAGAGTCTCTCTGCTTTTCTTGTTTACATGATTGATCGCTACCCGGATTGTCTCTCCTCTTCCTCTAGCCCCTTCAATAGTCTACTCAAACGC  300
Spenn_LA716_RDR     TATCAAGAAGAGTCTCTCTGCTTTTCTTGTTTACATGATTGATCGCTACCCGGATTGTCTCTCCTCTTCCTCTAGCCCCTTCAATAGTCTACTCAAACGC  300
Shabr_LYC4_RDR      TATCAAGAAGAGTCTCTCTGCTTTTCTTGTTTATATGATTGATCGCTACCCGGATTGTCTCTCCTCTTCCTCTAGCCCCTTCAATAGTCTACTCAAACGC  300
Sper_RDR            TATCAAGAAGACTCTCTCTGCTTTTCTTGTTTACATGATTGATCGCTACCCGGATTGTCTCTCCTCTTCCTCTAGCCCATCCAATACTCTACTCAAACGC  300
Schil_LA1969_Ty1    TATCAAGAAGAGTCTCTCTGCTTTTCTTGTTTACATGATTGATCGCTACCCGGATTGTCTCTCCTCTTCCTCTAGCCCATTCAATAGTCTACTCAAACGC  300
Schil_LA2779_Ty3    TATCAAGAAGAGTCTCTCTGCTTTTCTTGTTTACATGATTGATCGCTACCCGGATTGTCTCTCCTCTTCCTCTAGCCCCTTCAATAGTCTACTCAAACGC  300
Schil_Gh13_RDR      TATCAAGAAGAGTCNNNNNNNNN-----------------------------------------------------------------------------  110
Schil_BTI-87_RDR    TATCAAGAAGAGTCTCTCTGCTTTTCTTGTTTACATGATCGATCGCTACCCGGATTGTCTCTCCTCTTCCTCTAGCCCCTTCAATAGTCTACTCAAACGC  288
Schil_LA1932_RDR    TATCAAGAAGAGTCTCTCTGCTTTTCTTGTTTACATGATCGATCGCTACCCGGATTGTCTCTCCTCTTCCTCTAGCCCATCCAATAGTCTACTCAAACGC  300
Schil_LA1938_RDR    TATCAAGAAGAGTCTCTCTGCTTTTCTTGTTTACATGATTGATCGCTACCCGGATTGTCTCTCCTCTTCCTCTAGCCCCTTCAATAGTCTACTCAAACGC  300
Schil_LA1971_RDR    TATCAAGAAGAGTCTCTCTGCTTTTCTTGTTTACATGATCGATCGCTACCCGGATTGTCTCTCCTCTTCCTCTAGCCCATCCAATAGTCTACTCAAACGC  300
Schil_G1_1556_RDR   TATCAAGAAGASTCTCTCTGCTTTTCTTGTTTACATGATTGATCGCTACCCGGATTGTCTCTCCTCTTCCTCTAGCCCMTCCAATASTCTACTCAAACGC  300
Schil_G1_1558_RDR   TATCAAGAAGAGTCTCTCTGCTTTTCTTGTTTACATGATCGATCGCTACCCGGATTGTCTCTCCTCTTCCTCTAGCCCATCCAATAGTCTACTCAAACGC  300
```

```
                                                  exon 2
XM_010323869       TCTTCTTCCCCTCGTCTCTTTCCATCTCCAGAGGGTAAACGTTTACAAGGTGAAAGTTCTTCTAAATCAAAGCTTGAGATGGGCTTATTGGCCTGTGCAA   388
Slyc_MM_RDR        TCTTCTTCCCCTCGTCTCTTTCCATCTCCAGAGGGTAAACGTTTACAAGGTGAAAGTTCTTCTAAATCAAAGCTTGAGATGGGCTTATTGGCCTGTGCAA   388
Slyc_M82_RDR       TCTTCTTCCCCTCGTCTCTTTCCATCTCCAGAGGGTAAACGTTTACAAGGTGAAAGTTCTTCTAAATCAAAGCTTGAGATGGGCTTATTGGCCTGTGCAA   388
Spimp_LA1589_RDR   TCTTCTTCCCCTCGTCTCTTTCCATCTCCAGAGGGTAAACGTTTACAAGGTGAAAGTTCTTCTAAATCAAAGCTTGAGATGGGCTTATTGGCCTGTGCAA   388
Sarc_LA2157_RDR    TCTTCTTCCCCTCGTCTATTTCCATCTCCAGAGGGTAAACGTTTACAAGGTGAAAGTTCTTCTAAATCAAAGCTTGAGATGGGCTTATTGGCCTGTGCAA   400
Spenn_LA716_RDR    TCTTCTTCCCCTCTTCTCTTTCCATCTCCAGAGGGTAAACGTGTACAAGGTGAAAGTTCTTCTAAATCAAAGCTTGAGATGGGCTTCTTGGCCTGTGCAA   400
Shabr_LYC4_RDR     TCTTCTTCCCCTCTTCTGTTTCCATCTCCAGAGGGTAAACGTGTACAAGGTGAAAGTTCTTCTAAATCAAAGCATGAGATGGGCTTCTTGGCCTGTGCAA   400
Sper_RDR           TCTTCTTCCCCTCTTCTCTTTCCATCTCCAGAGGGTAAACGTTTACAAGGTGAAAGTTCTTCTAAATCAAAGCTTGAGATGGGCTTATTGGCCTGTGCAA   400
Schil_LA1969_Ty1   TCTTCTTCCCCTGTTCTATTTCCATCTCCAGAGGGTAAACGTTTACAAGGTGAAAGTTCTTCTAAATCAAAGCTTGAGATGGGCTTATTGGCCTGTGCAA   400
Schil_LA2779_Ty3   TCTTCTTCCCCTCTTCTATTTCCATCTCCAGAGGGTAAACGTTTACTTGGTGAAAGTTCTTCTAAATCAAAGCTTGAGATGGGCTTATTGGCCTGTGCAA   400
Schil_Gh13_RDR     -------------------------------AGGGTAAACGTTTACTTGGTGAAAGTTCTTCTAAATCAAAGCTTGAGATGGGCTTATTGGCCTGTGCAA   179
Schil_BTI-87_RDR   TCTTCTTCCCCTCGTCTCTTTCCATCTCCAGAGGGTAAACGTTTACAAGGTGAAAGTTCTTCTAAATCAAAGCTTGAGATGGGCTTATTGGCCTGTGCAA   388
Schil_LA1932_RDR   TCTTCTTCCCCTCTTCTCTTTCCATCTCCAGAGGGTAAACGTTTACAAGGTGAAAGTTCTTCTAAATCAAAGCTTGAGATGGGCTTATTGGCCTGTGCAA   400
Schil_LA1938_RDR   TCTTCTTCCCCTGTTCTATTTCCATCTCCAGAGGGTAAACGTTTACAAGGTGAAAGTTCTTCTAAATCAAAGCTTGAGATGGGCTTATTGGCCTGTGCAA   400
Schil_LA1971_RDR   TCTTCTTCCCCTGTTCTCTTTCCATCTCCAGAGGGTAAACGTTTACAAGGTGAAAGTTCTTCTAAATCAAAGCTTGAGATGGGCTTATTGGCCTGTGCAA   400
Schil_G1_1556_RDR  TCTTCTTCCCCTCTTCTMTTTCCATCTCCAGAGGGTAAACGTTTACAAGGTGAAAGTTCTTCTAAATCAAAGCWTGAGATGGGCTTMTTGGCCTGTGCAA   400
Schil_G1_1558_RDR  TCTTCTTCCCCTGTTCTCTTTCCATCTCCAGAGGGTAAACGTTTACAAGGTGAAAGTTCTTCTAAATCAAAGCTTGAGATGGGCTTATTGGCCTGTGCAA   400

XM_010323869       GCCCTCAGAAAGTTGCTCGCCAGTTATCATTTTGCGAGGAGCCTGAATCTAACTGTAGAAGAACCTCCCCTTATGTCAGCCAACAGTTGATGATCCTCAA   488
Slyc_MM_RDR        GCCCTCAGAAAGTTGCTCGCCAGTTATCATTTTGCGAGGAGCCTGAATCTAACTGTAGAAGAACCTCCCCTTATGTCAGCCAACAGTTGATGATCCTCAA   488
Slyc_M82_RDR       GCCCTCAGAAAGTTGCTCGCCAGTTATCATTTTGCGAGGAGCCTGAATCTAACTGTAGAAGAACCTCCCCTTATGTCAGCCAACAGTTGATGATCCTCAA   488
Spimp_LA1589_RDR   GCCCTCAGAAAGTTGCTCGCCAGTTATCATTTTGCGAGGAGCCTGAATCTAACTGTAGAAGAACCTCCCCTTATGTCAGCCAACAGTTGATGATCCTCAA   488
Sarc_LA2157_RDR    GCCCTCAGAAAGTTGCTTGCCAGTTATCATTTTGCGAGGAGCCTGAATCTAACTGTAGAAGAACCTCCCCTTATGTCAGCCAACAGTTGATGATCCTCAA   500
Spenn_LA716_RDR    GCCCTCAGAAAGTTGCTCGCCAGTTATCATTTTGCGAGGAGCCTGAATCTAACAGTAGAAGAACCTCACCTTATGTCAGCCAACAGTTGATGATCCTCAA   500
Shabr_LYC4_RDR     GCCCTCAGAAAGTTGCTCGCCAGTTATCATTTTGCGAGGAGCCTGAATCCAACTGTAGAAGAACCTCACCTTATGTCAGCCAACAGTTGATGATCCTCAA   500
Sper_RDR           GCCCTCAGAAAGTTGCTCGCCAGTTATCATTTTGCGAGGAGCCTGAATCTAACTGTAGAAGAACCTCCCCTTATGTCAGCCAACAGTTGATGATCCTCAA   500
Schil_LA1969_Ty1   GCCCTCAGAAAGTTGCTCGCCAGTTATCATTTTGCGAGGAGCCTGAATCTAACTGTAGAAGAACCTCCCCTTATGTCAGCCAACAGTTGATGATCCTCAA   500
Schil_LA2779_Ty3   GCCCTCAGAAAGTTGCTCGCCAGTTATCATTTTGCGAGGAGCCTGAATCTAACTGTAGAAGAACCTCCCCTTATGTCAGCCAACAGTTGATGATCCTCAA   500
Schil_Gh13_RDR     GCCCTCAGAAAGTTGCTCGCCAGTTATCATTTTGCGAGGAGCCTGAATCTAACTGTAGAAGAACCTCCCCTTATGTCAGCCAACAGTTGATGATCCTCAA   279
Schil_BTI-87_RDR   GCCCTCAGAAAGTTGCTCGCCAGTTATCATTTTGCGAGGAGCCTGAATCTAACTGTAGAAGAACCTCCCCTTATGTCAGCCAACAGTTGATGATCCTCAA   488
Schil_LA1932_RDR   GCCCTCAGAAAGTTGCTCGCCAGTTATCATTTTGCGAGGAGCCTGAATCTAACTGTAGAAGAACCTCCCCTTATGTCAGCCAACAGTTGATGATCCTCAA   500
Schil_LA1938_RDR   GCCCTCAGAAAGTTGCTCGCCAGTTATCATTTTGCGAGGAGCCTGAATCTAACTGTAGAAGAACCTCCCCTTATGTCAGCCAACAGTTGATGATCCTCAA   500
Schil_LA1971_RDR   GCCCTCAGAAAGTTGCTCGCCAGTTATCATTTTGCGAGGACCCTGAAGCTAACTGTAGAAGAACCTCCCCTTATGTCAGCCAACAGTTGATGATCCTCAA   500
Schil_G1_1556_RDR  GCCCTCAGAAAGTTGCTCGCCAGTTATCATTTTGCGAGGAGCCTGAATCTAACTGTAGAAGAACCTCMCCTTATGTCAGCCAACAGTTGATGATCCTCAA   500
Schil_G1_1558_RDR  GCCCTCAGAAAGTTGCTCGCCAGTTATCATTTTGCGAGGAGCCTGAATCTAACTGTAGAAGAACCTCACCTTATGTCAGCCAACAGTTGATGATCCTCAA   500

                                                                       exon 3
XM_010323869       TGAACTTGAATTTAGAAAATTGTTTCTGGTACTGAGCTACATTGGATGCAACAAGTTGGAAGATGTTATATCCCCTCAAATTGCTGATGATATTGTAAGA   588
Slyc_MM_RDR        TGAACTTGAATTTAGAAAATTGTTTCTGGTACTGAGCTACATTGGATGCAACAAGTTGGAAGATGTTATATCCCCTCAAATTGCTGATGATATTGTAAGA   588
Slyc_M82_RDR       TGAACTTGAATTTAGAAAATTGTTTCTGGTACTGAGCTACATTGGATGCAACAAGTTGGAAGATGTTATATCCCCTCAAATTGCTGATGATATTGTAAGA   588
Spimp_LA1589_RDR   TGAACTTGAATTTAGAAAATTGTTTTTGGTACTGAGCTACATTGGATGCAACAAGTTGGAAGATGTTATATCCCCTCAAATTGCTGATGATATTGTAAGA   588
Sarc_LA2157_RDR    TGAACTTGAATTTAGAAAATTGTTTTTGGTACTGAGCTACATTGGATGCAACAAGTTGGAAGATGTTATATCCCCTCAAATTGCTGATGATATTGTAAGA   600
Spenn_LA716_RDR    TGAACTCGAATTTAGAAAATTGTTTTTGGTACTGAGCTACATTGGATGCAACAAGTTGGAAGATGTTATATCCCCTCAAATTGCTGATGATATTGTAAGA   600
Shabr_LYC4_RDR     TGAACTTGAATTTAGAAAATTGTTTTTGGTACTGAGCTACATTGGATGCAACAAGTTGGAAGATGTTATATCCCCTCAAATTGCTGATGATATTGTAAGA   600
Sper_RDR           TGAACTTGAATTTAGAAAATTGTTTTTGGTACTGAGCTACATTGGATGCAACAAGTTGGAAGATGTTATATCCCCTCAAATTGCTGATGATATTGTAAGA   600
Schil_LA1969_Ty1   TGAACTTGAATTTAGAAAATTGTTTTTGGTACTGAGCTACATTGGATGCAACAAGTTGGAAGATGTTATATCCCCTCAAATTGCTGATGATATTGTAAGA   600
Schil_LA2779_Ty3   TGAACTTGAATTTAGAAAATTGTTTTTGGTACTGAGCTACATTGGATGCAACAAGTTGGAAGATGTTATATCCCCTCAAATTGCTGATGATATTGTAAGA   600
Schil_Gh13_RDR     TGAACTTGAATTTAGAAAATTGTTTTTGGTACTGAGCTACATTGGATGCAACAAGTTGGAAGATGTTATATCCCCTCAAATTGCTGATGATATTGTAAGA   379
Schil_BTI-87_RDR   TGAACTTGAATTTAGAAAATTGTTTTTGGTACTGAGCTACATTGGATGCAACAAGTTGGAAGATGTTATATCCCCTCAAATTGCTGATGATATTGTAAGA   588
Schil_LA1932_RDR   TGAACTTGAATTTAGAAAATTGTTTTTGGTACTGAGCTACATTGGATGCAACAAGTTGGAAGAAGTTATATCCCCTCAAATTGCTGATGATATTGTAAGA   600
Schil_LA1938_RDR   TGAACTTGAATTTAGAAAATTGTTTTTGGTACTGAGCTACATTGGATGCAACAAGTTGGAAGATGTTATATCCCCTCAAATTGCTGATGATATTGTAAGA   600
Schil_LA1971_RDR   TGAACTTGAATTTAGAAAATTGTTTTTGGTACTGAGCTACATTGGATGCAACAAGTTGGAAGATGTTATATCCCCTCAAATTGCTGATGATATTGTAAGA   600
Schil_G1_1556_RDR  TGAACTTGAATTTAGAAAATTGWTTTTGGTACTGAGCTACATKGGATGCAACAAGTTGGAAGATGTTATATCMCCTCAAATTGCTGATGAWATTGTAAGA   600
Schil_G1_1558_RDR  TGAACTTGAATTTAGAAAATTGTTTTTGGTGCTGAGCTACATTGGATGCAACAAGTTGGAAGATGTTATATCACCTCAAATTGCTGATGAAATTGTAAGA   600
```

```
                                                                                                  exon 4
XM_010323869      AAGAAAAATCTTTCCATGACTGATTTTGAATCAGAAATTTGGAATGCTTTTGGAAAAGCATGTTATGCTGTGTCAGATAGATCAAAGTACTTAGACTGGA   688
Slyc_MM_RDR       AAGAAAAATCTTTCCATGACTGATTTTGAATCAGAAATTTGGAATGCTTTTGGAAAAGCATGTTATGCTGTGTCAGATAGATCAAAGTACTTAGACTGGA   688
Slyc_M82_RDR      AAGAAAAATCTTTCCATGACTGATTTTGAATCAGAAATTTGGAATGCTTTTGGAAAAGCATGTTATGCTGTGTCAGATAGATCAAAGTACTTAGACTGGA   688
Spimp_LA1589_RDR  AAGAAAAATCTTTCAATGACTGATTTTGAATCAGAAATTTGGAATGCTTTTGGAAAAGCATGTTATGCTGTGTCAGATAGATCAAAGTACTTAGACTGGA   688
Sarc_LA2157_RDR   AAGAAAAATCTTTCCATGACTGATTTTGAATCAGAAATTTGGAATGCTTTTGGAAAAGCATGTTATGCTGTGTCAGATAGATCAAAGTACTTAGACTGGA   700
Spenn_LA716_RDR   AAGAATAATCTTTCCATGACTGATTTTGAATCAGAAATTTGGAATGCTTTTGGAAAAGCATGTTATGCTGTGTCAGATAGATCAAAGTACTTAGACTGGA   700
Shabr_LYC4_RDR    AAGAAAAATCTTTCCATGACTGATTTTGAATCAGAAATTTGGAATGCTTTTGGAAAAGCATGTTATGCTGTGTCAGATAGATCAAAGTACTTAGACTGGA   700
Sper_RDR          AAGAAAAATCTTTCCATGACTGATTTTGAATCAGAAATTTGGAATGCTTTTGGAAAAGCATGTTATGCTGTGTCAGATAGATCAAAGTACTTAGACTGGA   700
Schil_LA1969_Ty1  AAGAAAGATCTTTCCATGACTGATTTTGAATCAGAAATTTGGAATGCTTTTGGAAAAGCATGTTATGCTGTGTCAGATAGATCAAAGTACTTAGACTGGA   700
Schil_LA2779_Ty3  AAGAAAGATCTTTCCATGACTGATTTTGAATCAGAAATTTGGAATGCTTTTGGAAAAGCATGTTATGCTGTGTCAGATAGATCAAAGTACTTAGACTGGA   700
Schil_Gh13_RDR    AAGAAAGATCTTTCCATGACTGATTTTGAATCAGAAATTTGGAATGCTTTTGGAAAAGCATGTTATGCTGTGTCAGATAGATCAAAGTACTTAGACTGGA   479
Schil_BTI-87_RDR  AAGAAAGATCTTTCCATGACTGATTTTGAATCAGAAATTTGGAATGCTTTTGGAAAAGCATGTTATGCTGTGTCAGATAGATCAAAGTACTTAGACTGGA   688
Schil_LA1932_RDR  AAGAAAAATCTTTCCATGACTGATTTTGAATCAGAAATTTGGAATGCTTTTGGAAAAGCATGTTATGCTGTGTCAGATAGATCAAAGTACTTAGACTGGA   700
Schil_LA1938_RDR  AAGAAAGATCTTTCCATGACTGATTTTGAATCAGAAATTTGGAATGCTTTTGGAAAAGCATGTTATGCTGTGTCAGATAGATCAAAGTACTTAGACTGGA   700
Schil_LA1971_RDR  AAGAAAGATCTTTCCATGACTGATTTTGAATCAGAAATTTGGAATGCTTTTGGAAAAGCATGTTATGCTGTGTCAGATAGATCAAAGTACTTAGACTGGA   700
Schil_G1_1556_RDR AAGAAAAATCTTTCCATGACTGATTTTGAATCAGAAATTTGGAATGCTTTTGGAAAAGCATGTTATGCTGTGTCAGATAGATCAAAGTACTTAGACTGGA   700
Schil_G1_1558_RDR AAGAAAAATCTTTCCATGACTGATTTTGAATCAGAAATTTGGAATGCTTTTGGAAAAGCATGTTATGCTGTGTCAGATAGATCAAAGTACTTAGACTGGA   700
```

```
                                                                                exon 5
XM_010323869      ATTGCAGAAAGACACATATCTACTATTGCCACATTAAGCAGAACGGATACTGTTCCTTCAAGGGTCCATACTTGAACACATTAAGGACTCACTTACAGAG   788
Slyc_MM_RDR       ATTGCAGAAAGACACATATCTACTATTGCCACATTAAGCAGAACGGATACTGTTCCTTCAAGGGTCCATACTTGAACACATTAAGGACTCACTTACAGAG   788
Slyc_M82_RDR      ATTGCAGAAAGACACATATCTACTATTGCCACATTAAGCAGAACGGATACTGTTCCTTCAAGGGTCCATACTTGAACACATTAAGGACTCACTTACAGAG   788
Spimp_LA1589_RDR  ATTGCAGAAAGACACATATCTACTATTGCCACATTAAGCAGAACGGATACTGTACCTTCAAGGGTCCATACTTGAACACATTAAGGACTCACTTACAGAG   788
Sarc_LA2157_RDR   ATTGCAGAAAGACACACATCTACTATTGCCACATTAAGCAGAACGGATACTGTACCTTCAAGGGTCCATACTTGAACACAGTAAGGACTCACTTACAGAG   800
Spenn_LA716_RDR   ATTGCAGAAAGACACATATCTACTATTGCCACATTAAGCAGAACGGATACTGTACCTTCAAGGGTCCATACTTGAACACAGTAAGGACTCACTTACAGAG   800
Shabr_LYC4_RDR    ATTGCAGAAAGACACATATCTACTATTGCCACATTAAGCAGAACGGATACTGTACCTTCAAGGGTCCATACTTGAATACAGCAAGGACTCACTTACAGAG   800
Sper_RDR          ATTGCAGAAAGACACATATCTACTATTGCCACATTAAGCAGAACGGATACTGTACCTTCAAGGGTCCATACTTGAACACAGCAAGGACTCACTTACAGAG   800
Schil_LA1969_Ty1  ATTGCAGAAAGACACATATCTACTATTGCCACATTAAGCAGAACGGATGCTGTACCTTCAAGGGTCCATACTTGAACACAGCAAGGACTCACTTACAGAG   800
Schil_LA2779_Ty3  ATTGCAGAAAGACACATATCTACTATTGCCACATTAAGCAGAACGGATGCTGTACCTTCAAGGGTCCATACTTGAACACAGCAAGGACTCACTTACAGAG   800
Schil_Gh13_RDR    ATTGCAGAAAGACACATATCTACTATTGCCACATTAAGCAGAACGGATGCTGTACCTTCAAGGGTCCATACTTGAACACAGCAAGGACTCACTTACAGAG   579
Schil_BTI-87_RDR  ATTGCAGAAAGACACATATCTACTATTGCCACATTAAGCAGAACGGATGCTGTACCTTCAAGGGTCCATACTTGAACACAGCAAGGACTCACTTACAGAG   788
Schil_LA1932_RDR  ATTGCAGAAAGACACATATCTACTATTGCCACATTAAGCAGAACGGATGCTGTACCTTCAAGGGTCCATACTTGAACACAGCAAGGACTCACTTACAGAG   800
Schil_LA1938_RDR  ATTGCAGAAAGACACATATCTACTATTGCCACATTAAGCAGAACGGATGCTGTACCTTCAAGGGTCCATACTTGAACACAGCAAGGACTCACTTACAGAG   800
Schil_LA1971_RDR  ATTGCAGAAAGACACATATCTACTATTGCCACATTAAGCAGAACGGATGCTGTACCTTCAAGGGTCCATACTTGAACACAGCAAGGACTCACTTACAGAG   800
Schil_G1_1556_RDR ATTGCAGAAAGACACATATCTACTATTGCCACATTAAGCAGAACGGATGCTGTACCTTCAAGGGTCCATACTTGAACACAGCAAGGACTCACTTACAGAG   800
Schil_G1_1558_RDR ATTGCAGAAAGACACATATCTACTATTGCCACATTAAGCAGAACGGATGCTGTACCTTCAAGGGTCCATACTTGAACACAGCAAGGACTCACTTACAGAG   800
```

```
XM_010323869      AGCCCTGGGAGATGACAATGTACTGATTGTAAAATTTGTTGAAGATACAAGTTGTGCCAATATAATTCTCGAGGAAGGCATTCTTGTTGGCTTGAGACGT   888
Slyc_MM_RDR       AGCCCTGGGAGATGACAATGTACTGATTGTAAAATTTGTTGAAGATACAAGTTGTGCCAATATAATTCTCGAGGAAGGCATTCTTGTTGGCTTGAGACGT   888
Slyc_M82_RDR      AGCCCTGGGAGATGACAATGTACTGATTGTAAAATTTGTTGAAGATACAAGTTGTGCCAATATAATTCTCGAGGAAGGCATTCTTGTTGGCTTGAGACGT   888
Spimp_LA1589_RDR  AGCCCTGGGAGATGACAATGTACTGATTGTAAAATTTGTTGAAGATACAAGTTGTGCCAATATAATTCTCGAGGAAGGCATTCTTGTTGGCTTGAGACGT   888
Sarc_LA2157_RDR   AGCCCTGGGAGATGACAATGTACTGATTGTAAAATTTGTTGAAGATACAAGTTGTGCCAATGTAATTCTCGAGGAAGGCATTCTTGTTGGCTTGAGACGT   900
Spenn_LA716_RDR   AGCCCTGGGAGATGACAATGTACTGATTGTAAAATTTGTTGAAGATACAAGTTGTGCCAATATAATTCTCGAGGAAGGCATTCTTGTTGGCTTGAGACGT   900
Shabr_LYC4_RDR    AGCCCTGGGAGATGACAATGTACTGATTGTAAAATTTGTTGAAGATACAAGTTGTGCCAATATAATTCTCGAGGAAGGCATTCTTGTTGGCTTGAGACGT   900
Sper_RDR          AGCCCTGGGAGATGACAATGTACTGATTGTCAAATTTGTTGAAGATACAAGTTGTGCCAATATAATTCTCGAGGAAGGCATTCTTGTTGGCTTGAGACGT   900
Schil_LA1969_Ty1  AGCCCTGGGAGATGACAATGTACTGATTGTCAAATTTGTTGAAGATACAAGTTGTGCCAATATAATTCTCGAGGAAGGCATTCTTGTTGGCTTGAGACGT   900
Schil_LA2779_Ty3  AGCCCTGGGAGATGACAATGTACTGATTGTCAAATTTGTTGAAGATACAAGTTGTGCCAATATAATTCTCGAGGAAGGCATTCTTGTTGGCTTGAGACGT   900
Schil_Gh13_RDR    AGCCCTGGGAGATGACAATGTACTGATTGTCAAATTTGTTGAAGATACAAGTTGTGCCAATATAATTCTCGAGGAAGGCATTCTTGTTGGCTTGAGACGT   679
Schil_BTI-87_RDR  AGCCCTGGGAGATGACAATGTACTGATTGTAAAATTTGTTGAAGATACAAGTTGTGCCAATATAATTCTTGAGGAAGGCATTCTTGTTGGCTTGAGACGT   888
Schil_LA1932_RDR  AGCCCTGGGAGATGACAATGTACTGATTGTAAAATTTGTTGAAGATACAAGTTGTGCCAATATAATTCTTGAGGAAGGCATTCTTGTTGGCTTGAGACGT   900
Schil_LA1938_RDR  AGCCCTGGGAGATGACAATGTACTGATTGTCAAATTTGTTGAAGATACAAGTTGTGCCAATATAATTCTCGAGGAAGGCATTCTTGTTGGCTTGAGACGT   900
Schil_LA1971_RDR  AGCCCTGGGAGATGACAATGTACTGATTGTCAAATTTGTTGAAGATACAAGTTGTGCCAATATAATTCTCGAGGAAGGCATTCTTGTTGGCTTGAGACGT   900
Schil_G1_1556_RDR AGCCCTGGGAGATGACAATGTACTGATTGTCAAATTTGTTGAAGATACAAGTTGTGCCAATATAATTCTCGAGGAAGGCATTCTTGTTGGCTTGAGACGT   900
Schil_G1_1558_RDR AGCCCTGGGAGATGACAATGTACTGATTGTCAAATTTGTTGAAGATACAAGTTGTGCCAATATAATTCTCGAGGAAGGCATTCTTGTTGGCTTGAGACGT   900
```

```
                                     exon 6
XM_010323869         TACCGTTTCTTTGTGTATAAAGATGATAAAGAGAGGAAGAAAAGTCCAGCTATGATGAAGACAAAAACTGCTTCTTTGAAGTGCTACTTTGTTAGGTTTG   988
Slyc_MM_RDR          TACCGTTTCTTTGTGTATAAAGATGATAAAGAGAGGAAGAAAAGTCCAGCTATGATGAAGACAAAAACTGCTTCTTTGAAGTGCTACTTTGTTAGGTTTG   988
Slyc_M82_RDR         TACCGTTTCTTTGTGTATAAAGATGATAAAGAGAGGAAGAAAAGTCCAGCTATGATGAAGACAAAAACTGCTTCTTTGAAGTGCTACTTTGTTAGGTTTG   988
Spimp_LA1589_RDR     TACCGTTTCTTTGTGTATAAAGATGATAAAGAGAGGAAGAAAAGTCCAGCTATGATGAAGACAAAAACTGCTTCTTTGAAGTGCTACTTTGTTAGGTTTG   988
Sarc_LA2157_RDR      TACCGTTTCTTTGTGTATAAAGATGATAAAGAGAGGAAGAAAAGTCCAGCTATGATGAAGACAAAAACTGCTTCTTTGAAGTGCTACTTTGTTAGGTTTG  1000
Spenn_LA716_RDR      TACCGTTTCTTTGTGTATAAAGATGATAAAGAGAGGAAGAAAAGTCCAGCTATGATGAAGACAAAAACTGCTTCTTTGAAGTGCTACTTTGTTAGGTTTG  1000
Shabr_LYC4_RDR       TACCGTTTCTTTGTGTATAAAGATGATAAAGAGAGGAAGAAAAGTCCAGCTATGATGAAGACAAAAACTGCTTCTTTGAAGTGCTACTTTGTTAGGTTTG  1000
Sper_RDR             TACCGTTTCTTTGTGTATAAAGATGATAAAGAGAGGAAGAAAAGTCCAGCTATGATGAAGACAAAAACTGCTTCTTTGAAGTGCTACTTTGTTAGGTTTG  1000
Schil_LA1969_Ty1     TACCGTTTCTTTGTGTATAAAGATGATAAAGAGAGGAAGAAAAGTCCAGCTATGATGAAGACAAAAACTGCTTCTTTGAAGTGCTACTTTGTTAGGTTTG  1000
Schil_LA2779_Ty3     TACCGTTTCTTTGTGTATAAAGATGATAAAGAGAGGAAGAAAAGTCCAGCTATGATGAAGACAAAAACTGCTTCTTTGAAGTGCTACTTTGTTAGGTTTG  1000
Schil_Gh13_RDR       TACCGTTTCTTTGTGTATAAAGATGATAAAGAGAGGAAGAAAAGTCCAGCTATGATGAAGACAAAAACTGCTTCTTTGAAGTGCTACTTTGTTAGGTTTG   779
Schil_BTI-87_RDR     TACCGTTTCTTTGTGTATAAAGATGATAAAGAGAGGAAGAAAAGTCCAGCTATGATGAAGACAAAAACTGCTTCTTTGAAGTGCTACTTTGTTAGGTTTG   988
Schil_LA1932_RDR     TACCGTTTCTTTGTGTATAAAGATGATAAAGAGAGGAAGAAAAGTCCAGCTATGATGAAGACAAAAACTGCTTCTTTGAAGTGCTACTTTGTTAGGTTTG  1000
Schil_LA1938_RDR     TACCGTTTCTTTGTGTATAAAGATGATAAAGAGAGGAAGAAAAGTCCAGCTATGATGAAGACAAAAACTGCTTCTTTGAAGTGCTACTTTGTTAGGTTTG  1000
Schil_LA1971_RDR     TACCGTTTCTTTGTGTATAAAGATGATAAAGAGAGGAAGAAAAGTCCAGCTATGATGAAGACAAAAACTGCTTCTTTGAAGTGCTACTTTGTTAGGTTTG  1000
Schil_G1_1556_RDR    TACCGTTTCTTTGTGTATAAAGATGATAAAGAGAGGAAGAAAAGTCCAGCTATGATGAAGACAAAAACTGCTTCTTTGAAGTGCTACTTTGTTAGGTTTG  1000
Schil_G1_1558_RDR    TACCGTTTCTTTGTGTATAAAGATGATAAAGAGAGGAAGAAAAGTCCAGCTATGATGAAGACAAAAACTGCTTCTTTGAAGTGCTACTTTGTTAGGTTTG  1000

XM_010323869         AGTCCATTGGAACCTGCAATGATGGAGAATCCTATGTATTTTCTACCAAAACAATCAGTCAAGCAAGGTGTAAATTCATGCATGTGCATATGGTTTCTAA  1088
Slyc_MM_RDR          AGTCCATTGGAACCTGCAATGATGGAGAATCCTATGTATTTTCTACCAAAACAATCAGTCAAGCAAGGTGTAAATTCATGCATGTGCATATGGTTTCTAA  1088
Slyc_M82_RDR         AGTCCATTGGAACCTGCAATGATGGAGAATCCTATGTATTTTCTACCAAAACAATCAGTCAAGCAAGGTGTAAATTCATGCATGTGCATATGGTTTCTAA  1088
Spimp_LA1589_RDR     AGTCCATTGGAACCTGCAATGATGGAGAATCCTATGTATTTTCTACCAAAACAATCAGTCAAGCAAGGTGTAAATTCATGCATGTGCATATGGTTTCTAA  1088
Sarc_LA2157_RDR      AGTCCATTGGAACCTGCAATGATGGAGAATCCTATGTATTTTCTACCAAAACAATCAGTCAAGCAAGGTGTAAATTCATGCATGTGCATATGGTTTCTAA  1100
Spenn_LA716_RDR      AGTCCATTGGAACCTGCAATGATGGAGAATCCTATGTATTTTCTACCAAAACAATCAGTCAAGCAAGGTGTAAATTCATGCATGTGCATATGGTTTCTAA  1100
Shabr_LYC4_RDR       AGTCCATTGGAACCTGCAATGATGGAGAATCCTATGTATTTTCTACCAAAACAATCAGTCAAGCAAGGTGTAAATTCATGCATGTGCATATGGTTTCTAA  1100
Sper_RDR             AGTCCATTGGAACCTGCGATGATGGAGAATCCTATGTATTTTCTACCAAAACAATCAGTCAAGCAAGGTGTAAATTCATGCATGTGCATATGGTTTCTAA  1100
Schil_LA1969_Ty1     AGTCCATTGGAACCTGCGATGATGGAGAATCCTATGTATTTTCTACCAAAACAATCAGTCAAGCAAGGTGTAAATTCATGCATGTGCATATGGTTTCTAA  1100
Schil_LA2779_Ty3     AGTCTATTGGAACCTGCGATGATGGAGAATCCTATGTATTTTCTACCATAACAATCAGTCAAGCAAGGTGTAAATTCATGCATGTGCATATGGTTTCTAA  1100
Schil_Gh13_RDR       AGTCTATTGGAACCTGCGATGATGGAGAATCCTATGTATTTTCTACCATAACAATCAGTCAAGCAAGGTGTAAATTCATGCATGTGCATATGGTTTCTAA   879
Schil_BTI-87_RDR     AGTCCATTGGAACCTGCGATGATGGAGAATCCTATGTATTTTCTACCAAAACAATCAGTCAAGCAAGGTGTAAATTCATGCATGTGCATATGGTTTCTAA  1088
Schil_LA1932_RDR     AGTCCATTGGAACCTGCGATGATGGAGAATCCTATGTATTTTCTACCAAAACAATCAGTCAAGCAAGGTGTAAATTCATGCATGTGCATATGGTTTCTAA  1100
Schil_LA1938_RDR     AGTCCATTGGAACCTGCGATGATGGAGAATCCTATGTATTTTCTACCAAAACAATCAGTCAAGCAAGGTGTAAATTCATGCATGTGCATATGGTTTCTAA  1100
Schil_LA1971_RDR     AGTCCATTGGAACCTGCGATGATGGAGAATCCTATGTATTTTCTACCAAAACAGTCAGTCAAGCAAGGTGTAAATTCATGCATGTGCATATGGTTTCTAA  1100
Schil_G1_1556_RDR    AGTCCATTGGAACCTGCGATGATGGAGAATCCTATGTATTTTCTACCAAAACAATCAGTCAAGCAAGGTGTAAATTCATGCATGTGCATATGGTTTCTAA  1100
Schil_G1_1558_RDR    AGTCCATTGGAACCTGCGATGATGGAGAATCCTATGTATTTTCTACCAAAACAATCAGTCAAGCAAGGTGTAAATTCATGCATGTGCATATGGTTTCTAA  1100

                                          exon 7
XM_010323869         TATGGCAAAATATGCAGCCAGGCTTTCCTTAATTCTATCAAAGACGATTAAGCTTCAAACAGATCTTGATTCTGTCACCATTGAAAGAATTGAAGATATA  1188
Slyc_MM_RDR          TATGGCAAAATATGCAGCCAGGCTTTCCTTAATTCTATCAAAGACGATTAAGCTTCAAACAGATCTTGATTCTGTCACCATTGAAAGAATTGAAGATATA  1188
Slyc_M82_RDR         TATGGCAAAATATGCAGCCAGGCTTTCCTTAATTCTATCAAAGACGATTAAGCTTCAAACAGATCTTGATTCTGTCACCATTGAAAGAATTGAAGATATA  1188
Spimp_LA1589_RDR     TATGGCAAAATATGCAGCCAGGCTTTCCTTAATTCTATCAAAGACGATTAAGCTTCAAACAGATCTTGATTCTGTCACCATTGAAAGAATTGAAGATATA  1188
Sarc_LA2157_RDR      TATGGCAAAATATGCAGCCAGGCTTTCCTTAATTCTATCAAAGACGATTAAGCTTCAAACAGATCTTGATTCTGTCACCATTGAAAGAATTGAAGATATA  1200
Spenn_LA716_RDR      TATGGCAAAATATGCAGCCAGGCTTTCCTTAATTCTATCAAAGACGATTAAGCTTCAAACGGATCTTGATTCTGTCACCATTGAAAGAATTGAAGATATA  1200
Shabr_LYC4_RDR       TATGGCAAAATATGCAGCCAGGCTTTCCTTAATTCTATCAAAGACGATTAAGCTTCAAGCGGATCTTGATTCTGTCACCATTGAAAGAATTGAAGATATA  1200
Sper_RDR             TATGGCAAAATATGCAGCCAGGCTTTCCTTAATTCTATCAAAGACTATTAAGCTTCAAGCGGATCTTGATTCTGTCACCATTGAAAGAATCGAAGATATA  1200
Schil_LA1969_Ty1     TATGGCAAAATATGCAGCCAGGCTTTCCTTAATTCTATCAAAGACTATTAAGCTTCAAGTGGATCTTGATTCTGTCACCATTGAAAGAATCGAAGATATA  1200
Schil_LA2779_Ty3     TATGGCAAAATATGCAGCCAGGCTTTCCTTAATTCTATCAAAGACTATTAAGCTTCAAGTGGATCTTGATTCTGTCACCATTGAAAGAATTGAAGATATA  1200
Schil_Gh13_RDR       TATGGCAAAATATGCAGCCAGGCTTTCCTTAATTCTATCAAAGACTATTAAGCTTCAAACAGATCTTGATTCTGTCACCATTGAAAGAATTGAAGATATA   979
Schil_BTI-87_RDR     TATGGCAAAATACGCAGCCAGGCTTTCCTTAATTCTATCAAAGACTATTAAGCTTCAAGCGGATCTTGATTCTGTCACCATTGAAAGAATCGAAGATATA  1188
Schil_LA1932_RDR     TATGGCAAAATACGCAGCCAGGCTTTCCTTAATTCTATCAAAGACTATTAAGCTTCAAGCGGATCTTGATTCTGTCACCATTGAAAGAATCGAAGATATA  1200
Schil_LA1938_RDR     TATGGCAAAATATGCAGCCAGGCTTTCCTTAATTCTATCAAAGACTATTAAGCTTCAAGTGGATCTTGATTCTGTCACCATTGAAAGAATCGAAGATATA  1200
Schil_LA1971_RDR     TATGGCAAAATATGCAGCCAGGCTTTCCTTAATTCTATCAAAGACTATTAAGCTTCAAGTGGATCTTGATTCTGTCACCATTGAAAGAATCGAAGATATA  1200
Schil_G1_1556_RDR    TATGGCAAAATATGCAGCCAGGCTTTCCTTAATTCTATCAAAGACTATTAAGCTTCAAGTGGATCTTGATTCTGTCACCATTGAAAGAATCGAAGATATA  1200
Schil_G1_1558_RDR    TATGGCAAAATATGCAGCCAGGCTTTCCTTAATTCTATCAAAGACTATTAAGCTTCAAGTGGATCTTGATTCTGTCACCATTGAAAGAATCGAAGATATA  1200
```

```
                                  exon 8
XM_010323869          CTTTGTCGGGATGAAAATGGTTGTATTATTCAAGATGAAGACGGCGAACCTCGTATACATACTGATGGTACTGGTTTCATATCAGAAGATTTAGCTATGC  1288
Slyc_MM_RDR           CTTTGTCGGGATGAAAATGGTTGTATTATTCAAGATGAAGACGGCGAACCTCGTATACATACTGATGGTACTGGTTTCATATCAGAAGATTTAGCTATGC  1288
Slyc_M82_RDR          CTTTGTCGGGATGAAAATGGTTGTATTATTCAAGATGAAGACGGCGAACCTCGTATACATACTGATGGTACTGGTTTCATATCAGAAGATTTAGCTATGC  1288
Spimp_LA1589_RDR      CTTTGTCGGGATGAAAATGGTTGTATTATTCAAGATGAAGACGGCGAACCTCGTATACATACTGATGGTACTGGTTTCATATCAGAAGATTTAGCTATGC  1288
Sarc_LA2157_RDR       CTTTGTCGGGATGAAAATGGTTGTATTATTCAAGATGAAGACGGCGAACCTCGTATACATAGTGATGGTACTGGTTTCATATCAGAAGATTTAGCTATGC  1300
Spenn_LA716_RDR       CTTTGTCGGGATGAAAATGGTTGTATTATTCAAGATGAAGACGGCGAACCTCGTATACATACTGATGGGACTGGTTTCATATCAGAAGATTTAGCTATGC  1300
Shabr_LYC4_RDR        CTTTGTCGGGATGAAAATGGTTGTATTATTCAAGATGAAGACGGCGAACCTCGTATACATACTGATGGTACTGGTTTCATATCAGAAGATTTAGCTATGC  1300
Sper_RDR              CTTTGTCGGGATGAAAATGGTTGTATTATTCAAGATGAAGACGGCGAACCTCGTATACATACTGATGGTACTGGTTTCATATCAGAAGATTTAGCTATGC  1300
Schil_LA1969_Ty1      CTTTGTCGGGATGAAAATGGTTGTATTATTCAAGATGAAGACGGCGAACCTCGTATACATACTGATGGTACTGGTTTCATATCAGAAGATTTAGCTATGC  1300
Schil_LA2779_Ty3      CTTTGTCGGGATGAAAATGGTTGTATTATTCAAGATGAAGACGGCGAACCTCGTATACATACTGATGGTACTGGTTTCATATCAGAAGATTTAGCTATGC  1300
Schil_Gh13_RDR        CTTTGTCGGGATGAAAATGGTTGTATTATTCAAGATGAAGACGGCGAACCTCGTATACATACTGATGGTACTGGTTTCATATCAGAAGATTTAGCTATGC  1079
Schil_BTI-87_RDR      CTTTGTCGGGATGAAAATGGTTGTATTATTCAAGATGAAGACGGCGAACCTCGTATACATACTGATGGTACTGGTTTCATATCAGAAGATTTAGCTATGC  1288
Schil_LA1932_RDR      CTTTGTCGGGATGAAAATGGTTGTATTATTCAAGATGAAGACGGCGAACCTCGTATACATACTGATGGTACTGGTTTCATATCAGAAGATTTAGCTATGC  1300
Schil_LA1938_RDR      CTTTGTCGGGATGAAAATGGTTGTATTATTCAAGATGAAGACGGCGAACCTCGTATACATACTGATGGTACTGGTTTCATATCAGAAGATTTAGCTATGC  1300
Schil_LA1971_RDR      CTTTGTCAGGATGAAAATGGTTGTATTATTCAAGATGAAGACGGCGAACCTCGTATACATACTGATGGTACTGGTTTCATATCAGAAGATTTAGCTATGC  1300
Schil_G1_1556_RDR     CTTTGTCGGGATGAAAATGGTTGTATTATTCAAGATGAAGACGGCGAACCTCGTATACATACTGATGGTACTGGTTTCATATCAGAAGATTTAGCTATGC  1300
Schil_G1_1558_RDR     CTTTGTCGGGATAAAAATGGTTGTATTATTCAAGATGAAGACGGCGAACCTCGTATACATACTGATGGTACTGGTTTCATATCAGAAGATTTAGCTATGC  1300

                                                                         exon 9
XM_010323869          ATTGTCCCAAAGATTTTTCAAAAGCAGAATATATAAAAGATGAAAATTATGAGAATTTTGTTGATATCGTGGACCTTGATGACGTGAATGTAGAAAGAAG  1388
Slyc_MM_RDR           ATTGTCCCAAAGATTTTTCAAAAGCAGAATATATAAAAGATGAAAATTATGAGAATTTTGTTGATATCGTGGACCTTGATGACGTGAATGTAGAAAGAAG  1388
Slyc_M82_RDR          ATTGTCCCAAAGATTTTTCAAAAGCAGAATATATAAAAGATGAAAATTATGAGAATTTTGTTGATATCGTGGACCTTGATGACGTGAATGTAGAAAGAAG  1388
Spimp_LA1589_RDR      ATTGTCCCAAAGATTTTTCAAAAGCAGAATATATAAAAGATGAAAATTATGAGAATTTTGTTGATATCGTGGACCTTGATGACGTGAATGTAGAAAGAAG  1388
Sarc_LA2157_RDR       ATTGTCCCAAAGATTTTTCAAAAGCAGAATATATAAAAGATGAAAATTATGAGAATTTTGTTGATATCGTGGACCTTGATGACGTGAATGTAGAAAGAAG  1400
Spenn_LA716_RDR       ATTGTCCCAAAGATTTTTCAAAAGCAGAATATATAAAAGATGAAAATTATGAGAATTTTGTTGATATCGTGGACCTTGATGACGTGAATGTAGAAAGAAG  1400
Shabr_LYC4_RDR        ATTGTCCCAAAGATTTTTCAAAAGCAGAATATATAAAAGATGAAAATTATGAGNNNNNNNNNN-------------------------------------  1363
Sper_RDR              ATTGTCCCAAAGATTTTTCAAAAGCAGAATATATAAAAGATGAAAATTATGAGAATTTTGTTGATATCGTGGACCTTGATGACGTGAATGTAGAAAGAAG  1400
Schil_LA1969_Ty1      ATTGTCCCAAAGATTTTTCAAAAGCAGAATATATAAAAGATGAAAATTATGAGAATTTTGTTGATATCGTGGACCTTGATGACGTGAATGTAGAAAGAAG  1400
Schil_LA2779_Ty3      ATTGTCCCAAAGATTTTTCAAAAGCAGAATATATAAAAGATGAAAATTATGAGAATTTTGTTGATATCGTGGACCTTGATGACGTGAATGTAGAAAGAAG  1400
Schil_Gh13_RDR        ATTGTCCCAAAGATTTTTCAAAAGCAGAATATATAAAAGATGAAAATTATGAGAATTTTGTTGATATCGTGGACCTTGATGACGTGAATGTAGAAAGAAG  1179
Schil_BTI-87_RDR      ATTGTCCCAAAGATTTTTCAAAAGCAGAATATATAAAAGATGAAAATTATGAGAATTTTGTTGATATCGTGGACCTTGATGACGTGAATGTAGAAAGAAG  1388
Schil_LA1932_RDR      ATTGTCCCAAAGATTTTTCAAAAGCAGAATATATAAAAGATGAAAATTATGAGAATTTTGTTGATATCGTGGACCTTGATGACGTGAATGTAGAAAGAAG  1400
Schil_LA1938_RDR      ATTGTCCCAAAGATTTTTCAAAAGCAGAATATATAAAAGATGAAAATTATGAGAATTTTGTTGATATCGTGGACCTTGATGACGTGAATGTAGAAAGAAG  1400
Schil_LA1971_RDR      ATTGTCCCAAAGATTTTTCAAAAGCAGAATATATAAAAGATGAAAATTATGAGAATTTTGTTGATATCGTGGACCTTGATGACGTGAATGTAGAAAGAAG  1400
Schil_G1_1556_RDR     ATTGTCCCAAAGATTTTTCAAAAGCAGAATATATAAAAGATGAAAATTATGAGAATTTTGTTGATATCGTGGACCTTGATGACGTGAATGTAGAAAGAAG  1400
Schil_G1_1558_RDR     ATTGTCCCAAAGATTTTTCAAAAGCAGAATATATAAAAGATGAAAATTATGAGAATTTTGTTGATATCGTGGACCTTGATGACGTGAATGTAGAAAGAAG  1400

                                               exon 10
XM_010323869          AGTGAGTGTATCTCGCAATAGGAAACCGCCTTTGTTGATGCAGTGCCGTTTGTTCTTCAATGGTTGTGCTGTGAAGGGGACTTTTCTTGTCAATAGAAAG  1488
Slyc_MM_RDR           AGTGAGTGTATCTCGCAATAGGAAACCGCCTTTGTTGATGCAGTGCCGTTTGTTCTTCAATGGTTGTGCTGTGAAGGGGACTTTTCTTGTCAATAGAAAG  1488
Slyc_M82_RDR          AGTGAGTGTATCTCGCAATAGGAAACCGCCTTTGTTGATGCAGTGCCGTTTGTTCTTCAATGGTTGTGCTGTGAAGGGGACTTTTCTTGTCAATAGAAAG  1488
Spimp_LA1589_RDR      AGTGAGTGTATCTCGCAATAGGGAACCGCCTTTGTTGATGCAGTGCCGTTTGTTCTTCAATGGTTGTGCTGTGAAGGGGACTTTTCTTGTCAATAGAAAG  1488
Sarc_LA2157_RDR       AGTGAGTGTATCTCGCAATAGGGAACCGCCTTTGTTGATGCAGTGCCGTTTGTTCTTCAATGGTTGTGCTGTGAAGGGGACTTTTCTTGTCAATAGAAAG  1500
Spenn_LA716_RDR       AGCGAGTGTATCTCGCAATAGGGAACCGCCTTTGTTGATGCAGTGCCGTTTGTTCTTCAACGGTTGTGCTGTGAAGGGGACTTTTCTTGTCAATAGAAAG  1500
Shabr_LYC4_RDR        --------------------GGGAACCGCCTTTGTTGATGCAGTGCCGTTTGTTCTTCAACGGTTGTGCTGTGAAGGGGACTTTTCTTGTCAATAGAAAG  1443
Sper_RDR              AGCCAGTGTATCTGGGAATAGGGAACCGCCTTTGTTGATGCAGTGCCGTTTGTTCTTCAACGGTTGTGCTGTGAAGGGGACTTTTCTTGTCAATAGAAAG  1500
Schil_LA1969_Ty1      AGCGAGTGTATCTGGGAATAGGGAACCGCCTTTGTTGATGCAGTGCCGTTTGTTCTTCAACGGTTGTGCTGTGAAGGGGACTTTTCTTGTCAATAGAAAG  1500
Schil_LA2779_Ty3      AGCGAGTGTATCTGGGAATAGGGAACCGCCTTTGTTGATGCAGTGCCGTTTGTTCTTCAACGGTTGTGCTGTGAAGGGGACTTTTCTTGTCAATAGAAAG  1500
Schil_Gh13_RDR        AGCGAGTGTATCTGGGAATAGGGAACCGCCTTTGTTGATGCAGTGCCGTTTGTTCTTCAACGGTTGTGCTGTGAAGGGGACTTTTCTTGTCAATAGAAAG  1279
Schil_BTI-87_RDR      AGYGAGTGTATCTCGCAATAGGAAACCGCCTTTGTTGATGCAGTGCCGTTTGTTCTTCAAAGGTTGTGCTGTGAAGGGGACTTTTCTTCTCAATAGAAAG  1488
Schil_LA1932_RDR      AGCGAGTGTATCTGGGAAGAGGGAACCGCCTTTGTTGATGCAGTGCCGTTTGTTCTTCAAAGGTTGTGCTGTGAAGGGGACTTTTCTTCTCAATAGAAAG  1500
Schil_LA1938_RDR      AGCGAGTGTATCTGGGAATAGGGAACCGCCTTTGTTGATGCAGTGCCGTTTGTTCTTCAACGGTTGTGCTGTGAAGGGGACTTTTCTTGTCAATAGAAAG  1500
Schil_LA1971_RDR      AGCGAGTGTATCTGGGAATAGGGAACCGCCTTTGTTGATGCAGTGCCGTTTGTTCTTCAACGGTTGTGCTGTGAAGGGGACTTTTCTTGTCAATAGAAAG  1500
Schil_G1_1556_RDR     AGTGAGTGTATCTCGCAATAGGGAACCGCCTTTGTTGATGCAGTGCCGTTTGTTCTTCAATGGTTGTGCTGTGAAGGGGACTTTTCTTGTCAATAGAAAG  1500
Schil_G1_1558_RDR     AGCGAGTGTATCTGGGAATAGGGAACCGCCTTTGTTGATGCAGTGCCGTTTGTTCTTCAATGGTTGTGCTGTGAAGGGGACTTTTCTTGTCAATAGAAAG  1500
```

```
                  → exon 11
XM_010323869        ATTGGATCACGAAAAATTCATATTAGACCCTCAATGGTGAAGGTTGAGATAGACCCAACAATTTCAAGTATACCAACTTTTGACTCATTGGAGATAGTTG  1588
Slyc_MM_RDR         ATTGGATCACGAAAAATTCATATTAGACCCTCAATGGTGAAGGTTGAGATAGACCCAACAATTTCAAGTATACCAACTTTTGACTCATTGGAGATAGTTG  1588
Slyc_M82_RDR        ATTGGATCACGAAAAATTCATATTAGACCCTCAATGGTGAAGGTTGAGATAGACCCAACAATTTCAAGTATACCAACTTTTGACTCATTGGAGATAGTTG  1588
Spimp_LA1589_RDR    ATTGGATCACGAAAAATTCATATTAGACCCTCAATGGTGAAGGTTGAGATAGACCCAACAATTTCAAGTATACCAACTTTTGACTCATTGGAGATAGTTG  1588
Sarc_LA2157_RDR     ATTGGATCACGAAAAATTCATATTAGACCCTCAATGGTGAAGGTTGAGATAGACCCAACAATTTCAAGTATACCAACTTTTGACTCATTGGAGATAGTTG  1600
Spenn_LA716_RDR     ATTGGATCACGAAAAATTCATATTAGACCCTCAATGGTGAAGGTTGAGATAGATCCAACAATTTCAAGTATACCAACTTTTGACTCATTGGAGATAGTTG  1600
Shabr_LYC4_RDR      ATTGGATCACGAAAAATTCATATTAGACCCTCAATGGTGAAGGTTGAGATAGACCCAACAATTTCAAGTATACCAACTTTTGACTCATTGGAGATAGTTG  1543
Sper_RDR            ATTGGATCACGAAAAATTCATATTAGACCCTCAATGGTGAAGGTTGAGATAGACCCAACAATTTCAAGTATACCAACTTTTGACTCATTGGAGATAGTTG  1600
Schil_LA1969_Ty1    ATTGGATCACGAAAAATTCATATTAGACCCTCAATGGTGAAGGTTGAGATAGACCCAACAATTTCAAGTATACCAACTTTTGACTCATTGGAGATAGTTG  1600
Schil_LA2779_Ty3    ATTGGATCACGAAAAATTCATATTAGACCCTCAATGGTGAAGGTTGAGATAGACCCAACAATTTCAAGTATACCAACTTTTGACTCATTGGAGATAGTTG  1600
Schil_Gh13_RDR      ATTGGATCACGAAAAATTCATATTAGACCCTCAATGGTGAAGGTTGAGATAGACCCAACAATTTCAAGTATACCAACTTTTGACTCATTGGAGATAGTTG  1379
Schil_BTI-87_RDR    ATTGGATCACGAAAAATTCATATTAGACCCTCAATGGTGAAGGTTGAGATAGACCCAACAATTTCAAGTATACCAACTTTTGACTCATTGGAGATAGTTG  1588
Schil_LA1932_RDR    ATTGGATCACGAAAAATTCATATTAGACCCTCAATGGTGAAGGTTGAGATAGACCCAACAATTTCAAGTATACCAACTTTTGACTCATTGGAGATAGTTG  1600
Schil_LA1938_RDR    ATTGGATCACGAAAAATTCATATTAGACCCTCAATGGTGAAGGTTGAGATAGACCCAACAATTTCAAGTATACCAACTTTTGACTCATTGGAGATAGTTG  1600
Schil_LA1971_RDR    ATTGGATCACGAAAAATTCATATTAGACCCTCAATGGTGAAGGTTGAGATAGACCCAACAATTTCAAGTATACCAACTTTTGACTCATTGGAGATAGTTG  1600
Schil_G1_1556_RDR   ATTGGATCACGAAAAATTCATATTAGACCCTCAATGGTGAAGGTTGAGATAGACCCAACAATTTCAAGTATATCAACTTTTGACTCATTGGAGATAGTTG  1600
Schil_G1_1558_RDR   ATTGGATCACGAAAAATTCATATTAGACCCTCAATGGTGAAGGTTGAGATAGACCCAACAATTTCAAGTATATCAACTTTTGACTCATTGGAGATAGTTG  1600
                          → exon 12
XM_010323869        CAATCAGTCATAGACCAAATAAGGCATATCTGTCCAAGAATTTAATCTCTCTGCTGAGCTACGGAGGAGTCCATAAAGAATACTTTATGGAACTTTTGGG  1688
Slyc_MM_RDR         CAATCAGTCATAGACCAAATAAGGCATATCTGTCCAAGAATTTAATCTCTCTGCTGAGCTACGGAGGAGTCCATAAAGAATACTTTATGGAACTTTTGGG  1688
Slyc_M82_RDR        CAATCAGTCATAGACCAAATAAGGCATATCTGTCCAAGAATTTAATCTCTCTGCTGAGCTACGGAGGAGTCCATAAAGAATACTTTATGGAACTTTTGGG  1688
Spimp_LA1589_RDR    CAATCAGTCATAGACCAAATAAGGCATATCTGTCCAAGAATTTAATCTCTCTGCTGAGCTACGGAGGAGTCCATAAAGAATACTTTATGGAACTTTTGGG  1688
Sarc_LA2157_RDR     CAATCAGTCATAGACCAAATAAGGCATATCTGTCCAAGAATTTAATCTCTCTGCTGAGCTACGGAGGAGTCCATAAAGAATACTTTATGGAGCTTTTGGG  1700
Spenn_LA716_RDR     CAATCAGTCATAGACCAAATAAGGCATATCTGTCCAAGAATTTAATCTCTCTGCTGAGCTACGGAGGAGTCCATAAAGAATACTTTATGGAGCTTTTGGG  1700
Shabr_LYC4_RDR      CAATCAGTCATAGACCAAATAAGGCATATCTGTCCAAGAATTTAATCTCTCTGCTGAGCTACGGAGGAGTCCATAAAGAATACTTTATGGAGCTTTTGGG  1643
Sper_RDR            CAATCAGTCATAGACCAAATAAGGCATATCTGTCCAAGAATTTAATCTCTCTGCTGAGCTACGGAGGAGTCCATAGAGAATACTTTATGGAGCTTTTGGG  1700
Schil_LA1969_Ty1    CAATCAGTCATAGACCAAATAAGGCATATCTGTCCAAGAATTTAATCTCTCTGCTGAGCTACGGAGGAGTCCATAAAGAATACTTTCTGGAGCTTTTGGG  1700
Schil_LA2779_Ty3    CAATCAGTCATAGACCAAATAAGGCATATCTGTCCAAGAATTTAATCTCTCTGCTGAGCTACGGAGGAGTCCATAAAGAATACTTTCTGGAGCTTTTGGG  1700
Schil_Gh13_RDR      CAATCAGTCATAGACCAAATAAGGCATATCTGTCCAAGAATTTAATCTCTCTGCTGAGCTACGGAGGAGTCCATAAAGAATACTTTCTGGAGCTTTTGGG  1479
Schil_BTI-87_RDR    CAATCAGTCATAGACCAAATAAGGCATATCTGTCCAAGAATTTAATCTCTCTGCTGAGCTACGGAGGAGTCCATAAAGAATACTTTCTGGAGCTTTTGGG  1688
Schil_LA1932_RDR    CAATCAGTCATAGACCAAATAAGGCATATCTGTCCAAGAATTTAATCTCTCTGCTGAGCTACGGAGGAGTCCATAAAGAATACTTTCTGGAGCTTTTGGG  1700
Schil_LA1938_RDR    CAATCAGTCATAGACCAAATAAGGCATATCTGTCCAAGAATTTAATCTCTCTGCTGAGCTACGGAGGAGTCCATAAAGAATACTTTCTGGAGCTTTTGGG  1700
Schil_LA1971_RDR    CAATCAGTCATAGACCAAATAAGGCATATCTGTCCAAGAATTTAATCTCTCTGCTGAGCTACGGAGGAGTCCATAAAGAATACTTTCTGGAGCTTTTGGG  1700
Schil_G1_1556_RDR   CAATCAGTCATAGACCAAATAAGGCATATCTGTCCAAGAATTTAATCTCTCTGCTGAGCTACGGAGGAGTCCATAAAGAATACTTTATGGAGCTTTTGGG  1700
Schil_G1_1558_RDR   CAATCAGTCATAGACCAAATAAGGCATATCTGTCCAAGAATTTAATCTCTCTGCTGAGCTACGGAGGAGTCCATAAAGAATACTTTATGGAGCTTTTGGG  1700
                                                                                  → exon 13
XM_010323869        AAGTGCGCTGGAAGAGACGAAACAAGTATATTTGAGGAAACGTGCAGCTCTAAAAGTTGCTATCAACTATAGAGAAATGGATGATGAATGTCTAACAGCA  1788
Slyc_MM_RDR         AAGTGCGCTGGAAGAGACGAAACAAGTATATTTGAGGAAACGTGCAGCTCTAAAAGTTGCTATCAACTATAGAGAAATGGATGATGAATGTCTAACAGCA  1788
Slyc_M82_RDR        AAGTGCGCTGGAAGAGACGAAACAAGTATATTTGAGGAAACGTGCAGCTCTAAAAGTTGCTATCAACTATAGAGAAATGGATGATGAATGTCTAACAGCA  1788
Spimp_LA1589_RDR    AAGTGCGCTGGAAGAGACGAAACAAGTATATTTGAGGAAACGTGCAGCTCTAAAAGTTGCTATCAACTATAGAGAAATGGATGATGAATGTCTAACAGCA  1788
Sarc_LA2157_RDR     AAGTGCACTGGAAGAGACGAATCAAGTATATTTGAGGAAACGTGCAGCTCTAAAAGTTGCTATCAACTATAGAGAAATGGATGATGAATGTCTAACAGCA  1800
Spenn_LA716_RDR     AAGTGCACTGGAAGAGACGAAACAAGTACGTTTGAGGAAACGTGCAGCTCTAAAAGTTGCTATCAACTATAGAGAAATGGATGATGAATGTCTAACAGCA  1800
Shabr_LYC4_RDR      AAGTGCACTGGAAGAGACGAAACAAGTATATTTGAGGAAACGTGCAGCTCTAAAAGTTGCTATCAACTATAGAGAAATGGATGATGAATGTCTAACAGCA  1743
Sper_RDR            AAGTGCACTGGAAGAGACGAATCAAGTATATTTGAGGAAACGTGCAGCTCTAAAAGTTGCTATCAACTATAGAGAAATGGATGATGAATGTCTAACAGCA  1800
Schil_LA1969_Ty1    AAGTGCACTGGAAGAGACGAAACAAGTATATTTGAGGAAACGGGCAGCTCTAAAAGTTGCTATCAACTATAGAGAAATGGATGATGAATGTCTAACAGCA  1800
Schil_LA2779_Ty3    AAGTGCACTGGAAGAGACGAAACAAGTATATTTGAGGAAACGGGCAGCTCTAAAAGTTGCTATCAACTATAGAGAAATGGATGATGAATGTCTAACAGCA  1800
Schil_Gh13_RDR      AAGTGCACTGGAAGAGACGAAACAAGTATATTTGAGGAAACGGGCAGCTCTAAAAGTTGCTATCAACTATAGAGAAATGGATGATGAATGTCTAACAGCA  1579
Schil_BTI-87_RDR    AAGTGCACTGGAAGAGACGAAACAAGTATATTTGAGGAAACGGGCAGCTCTAAAAGTTGCTATCAACTATAGAGAAATGGATGATGAATGTCTAACAGCA  1788
Schil_LA1932_RDR    AAGTGCACTGGAAGAGACGAAACAAGTATATTTGAGGAAACGGGCAGCTCTAAAAGTTGCTATCAACTATAGAGAAATGGATGATGAATGTCTAACAGCA  1800
Schil_LA1938_RDR    AAGTGCACTGGAAGAGACGAAACAAGTATATTTGAGGAAACGGGCAGCTCTAAAAGTTGCTATCAACTATAGAGAAATGGATGATGAATGTCTAACAGCA  1800
Schil_LA1971_RDR    AAGTGCACTGGAAGAGACGAAACAAGTATATTTGAGGAAACGGGCAGCTCTAAAAGTTGCTATCAACTATAGAGAAATGGATGATGAATGTCTAACAGCA  1800
Schil_G1_1556_RDR   AAGTGCACTGGAAGAGACGAATCAAGTATATTTGAGGAAACGTGCAGCTCTAAAAGTTGCTATCAACTATAGAGAAATGGATGATGAATGTCTAACAGCA  1800
Schil_G1_1558_RDR   AAGTGCACTGGAAGAGACGAATCAAGTATATTTGAGGAAACGTGCAGCTCTAAAAGTTGCTATCAACTATAGAGAAATGGATGATGAATGTCTAACAGCA  1800
```

```
XM_010323869        AGGATGATATCGTCTGGGATACCTCTCAATGAACCTCATCTCCATGCTCGCTTGTCTAGGCTTGCAAAGATTGAAAGAACTAAGCTTAGAGGAGGAAAGC  1888
Slyc_MM_RDR         AGGATGATATCGTCTGGGATACCTCTCAATGAACCTCATCTCCATGCTCGCTTGTCTAGGCTTGCAAAGATTGAAAGAACTAAGCTTAGAGGAGGAAAGC  1888
Slyc_M82_RDR        AGGATGATATCGTCTGGGATACCTCTCAATGAACCTCATCTCCATGCTCGCTTGTCTAGGCTTGCAAAGATTGAAAGAACTAAGCTTAGAGGAGGAAAGC  1888
Spimp_LA1589_RDR    AGGATGATATCGTCTGGGATACCTCTCAATGAACCTCATCTCCATGCTCGCTTGTCTAGGCTTGCAAAGATTGAAAGAACTAAGCTTAGAGGAGGAAAGC  1888
Sarc_LA2157_RDR     AGGATGATATCATCTGGGATACCTCTCAATGAACCTCATCTCCATGCTCGCTTGTCTAGGCTTGCAAAGATTGAAAGAACTAAGCTTAGAGGAGGAAAGC  1900
Spenn_LA716_RDR     AGGATGATATCGTCTGGGATACCTCTCAATGAGCCTCATCTCCATGCTCGCTTGTCTAGGCTTGCAAAGATTGAAAGAACTAAGCTTAGAGGAGGAAAGC  1900
Shabr_LYC4_RDR      AGGATGATATCGTCTGGGATACCTCTCAATGAACCTCATCTCCATGCTCGCTTGTCTAGGCTTGCAAAGATTGAAAGAACTAAGCTTAGAGGAGGAAAGC  1843
Sper_RDR            AGGATGATATCGTCTGGGATACCTCTCAATGAACCTCATCTCCATGCTCGCTTGTCTAGGCTTGCAAAGATTGAAAGAACTAAGCTTAGAGGAGGAAAGC  1900
Schil_LA1969_Ty1    AGGATGATATCGTCTGGGATACCTCTCAATGAACCTCATCTCCATGTTCGCTTGTCTAGGCTTGCAAAGATTGAAAGAACTAAGCTTAGAGGAGGAAAGC  1900
Schil_LA2779_Ty3    AGGATGATATCGTCTGGGATACCTCTCAATGAACCTCATCTCCATGTTCGCTTGTCTAGGCTTGCAAAGATTGAAAGAACTAAGCTTAGAGGAGGAAAGC  1900
Schil_Gh13_RDR      AGGATGATATCGTCTGGGATACCTCTCAATGAACCTCATCTCCATGTTCGCTTGTCTAGGCTTGCAAAGATTGAAAGAACTAAGCTTAGAGGAGGAAAGC  1679
Schil_BTI-87_RDR    AGGATGATATCGTCTGGGATACCTCTCAATGAACCTCATCTCCATGTTCGCTTGTCTAGGCTTGCAAAGATTGAAAGAACTAAGCTTAGAGGAGGAAAGC  1888
Schil_LA1932_RDR    AGGATGATATCGTCTGGGATACCTCTCAATGAACCTCATCTCCATGTTCGCTTGTCTAGGCTTGCAAAGATTGAAAGAACTAAGCTTAGAGGAGGAAAGC  1900
Schil_LA1938_RDR    AGGATGATATCGTCTGGGATACCTCTCAATGAACCTCATCTCCATGTTCGCTTGTCTAGGCTTGCAAAGATTGAAAGAACTAAGCTTAGAGGAGGAAAGC  1900
Schil_LA1971_RDR    AGGATGATATCGTCTGGGATACCTCTCAATGAACCTCATCTCCATGTTCGCTTGTCTAGGCTTGCAAAGATTGAAAGAACTAAGCTTAGAGGAGGAAAGC  1900
Schil_G1_1556_RDR   AGGATGATATCGTCTGGGATACCTCTCAATGAACCTCATCTCCATGCTCGCTTGTCTAGGCTTGCAAAGATTGAAAGAACTAAGCTTAGAGGAGGAAAGC  1900
Schil_G1_1558_RDR   AGGATGATATCGTCTGGGATACCTCTCAATGAACCTCATCTCCATGCTCGCTTGTCTAGGCTTGCAAAGATTGAAAGAACTAAGCTTAGAGGAGGAAAGC  1900

                                                                                                             exon 14
XM_010323869        TTCCTATAAGTGACAGTTTTTATCTTATGGGAACAGCTGACCCCACTGGTGTACTGGAAAGCAATGAAGTCTGTGTTATTCTAGATAATGGCCAAGTATC  1988
Slyc_MM_RDR         TTCCTATAAGTGACAGTTTTTATCTTATGGGAACAGCTGACCCCACTGGTGTACTGGAAAGCAATGAAGTCTGTGTTATTCTAGATAATGGCCAAGTATC  1988
Slyc_M82_RDR        TTCCTATAAGTGACAGTTTTTATCTTATGGGAACAGCTGACCCCACTGGTGTACTGGAAAGCAATGAAGTCTGTGTTATTCTAGATAATGGCCAAGTATC  1988
Spimp_LA1589_RDR    TTCCTATAAGTGACAGTTTTTATCTTATGGGAACAGCTGACCCCACTGGTGTACTGGAAAGCAATGAAGTCTGTGTTATTCTAGATAATGGCCAAGTATC  1988
Sarc_LA2157_RDR     TTCCTATAAGTGACAGTTTTTATCTTATGGGAACAGCTGACCCCACTGGTGTACTGGAAAGCAATGAAGTCTGTGTTATTCTAGATAATGGCCAAGTATC  2000
Spenn_LA716_RDR     TTCCAATAAATGACAGTTTTTATCTTATGGGAACAGCTGACCCCACTGGTGTACTGGAAAGCAATGAAGTCTGTGTTATTCTAGATAATGGCCAAGTATC  2000
Shabr_LYC4_RDR      TTCCTATAAGTGACAGTTTTTATCTTATGGGAACAGCTGACCCCACTGGTGTACTGGAAAGCAATGAAGTCTGTGTTATTCTAGATAATGGCCAAGTATC  1943
Sper_RDR            TTCCTATAAGTGACAGTTTTTATCTTATGGGAACAGCTGACCCCACTGGTGTACTGGAAAGCAATGAAGTCTGTGTTATTCTAGATAATGGCCAAGTATC  2000
Schil_LA1969_Ty1    TTCCTATAAGTGACAGTTTTTATCTTATGGGAACAGCTGACCCCACTGGTGTACTGGAAAGCAATGAAGTCTGTGTTATTCTAGATAATGGCCAAGTATC  2000
Schil_LA2779_Ty3    TTCCTATAAGTGACAGTTTTTATCTTATGGGAACAGCTGACCCCACTGGTGTACTGGAAAGCAATGAAGTCTGTGTTATTCTAGATAATGGCCAAGTATC  2000
Schil_Gh13_RDR      TTCCTATAAGTGACAGTTTTTATCTTATGGGAACAGCTGACCCCACTGGTGTACTGGAAAGCAATGAAGTCTGTGTTATTCTAGATAATGGCCAAGTATC  1779
Schil_BTI-87_RDR    TTCCTATAAGTGACAGTTTTTATCTTATGGGAACAGCTGACCCCACTGGTGTACTGGAAAGCAATGAAGTCTGTGTTATTCTAGATAATGGCCAAGTATC  1988
Schil_LA1932_RDR    TTCCTATAAGTGACAGTTTTTATCTTATGGGAACAGCTGACCCCACTGGTGTACTGGAAAGCAATGAAGTCTGTGTTATTCTAGATAATGGCCAAGTATC  2000
Schil_LA1938_RDR    TTCCTATAAGTGACAGTTTTTATCTTATGGGAACAGCTGACCCCACTGGTGTACTGGAAAGCAATGAAGTCTGTGTTATTCTAGATAATGGCCAAGTATC  2000
Schil_LA1971_RDR    TTCCTATAAGTGACAGTTTTTATCTTATGGGAACAGCTGACCCCACTGGTGTACTGGAAAGCAATGAAGTCTGTGTTATTCTAGATAATGGCCAAGTATC  2000
Schil_G1_1556_RDR   TTCCTATAAGTGACAGTTTTTATCTTATGGGAACAGCTGACCCCACTGGTGTACTGGAAAGCAATGAAGTCTGTGTTATTCTAGATAATGGCCAAGTATC  2000
Schil_G1_1558_RDR   TTCCTATAAGTGACAGTTTTTATCTTATGGGAACAGCTGACCCCACTGGTGTACTGGAAAGCAATGAAGTCTGTGTTATTCTAGATAATGGCCAAGTATC  2000

XM_010323869        TGGGCGTGTTTTGGTCTATAGAAATCCTGGTCTTCACTTTGGAGATGTACATGTGATGAAAGCGCGATATGTGGAAGAGCTTGCAGATGTTGTTGGTGAT  2088
Slyc_MM_RDR         TGGGCGTGTTTTGGTCTATAGAAATCCTGGTCTTCACTTTGGAGATGTACATGTGATGAAAGCGCGATATGTGGAAGAGCTTGCAGATGTTGTTGGTGAT  2088
Slyc_M82_RDR        TGGGCGTGTTTTGGTCTATAGAAATCCTGGTCTTCACTTTGGAGATGTACATGTGATGAAAGCGCGATATGTGGAAGAGCTTGCAGATGTTGTTGGTGAT  2088
Spimp_LA1589_RDR    TGGGCGTGTTTTGGTCTATAGAAATCCTGGTCTTCACTTTGGAGATGTACATGTGATGAAAGCGCGATATGTGGAAGAGCTTGCAGATGTTGTTGGTGAT  2088
Sarc_LA2157_RDR     TGGGCGTGTTTTGGTCTATAGAAATCCTGGTCTTCACTTTGGAGATGTACATGTGATGAAAGCGCGATATGTGGAAGAGCTTGCAGATGTTGTTGGTGAT  2100
Spenn_LA716_RDR     TGGGCGTGTTTTGGTCTATAGAAATCCTGGTCTTCACTTTGGAGATGTACATGTGATGAAAGCGCGATATGTGGAAGAGCTTGCAGATGTTGTTGGTGAT  2100
Shabr_LYC4_RDR      TGGGCGTGTTTTGGTCTATAGAAATCCTGGTCTTCACTTTGGAGATGTACATGTGATGAAAGCGCGATATGTGGAAGAGCTTGCAGATGTTGTTGGTGAT  2043
Sper_RDR            TGGGCGTGTTTTGGTCTATAGAAATCCTGGTCTTCACTTTGGAGATGTACATGTGATGAAAGCGCGATATGTGGAAGAGCTTGCAGATGTTGTTGGTGAT  2100
Schil_LA1969_Ty1    TGGGCGTGTTTTGGTCTACAGAAATCCTGGTCTTCACTTTGGAGATGTGCATGTGATGAAAGCGCGATATGTGGAAGAGCTTGCAGATGTTGTTGGTGAT  2100
Schil_LA2779_Ty3    TGGGCGTGTTTTGGTCTACAGAAATCCTGGTCTTCACTTTGGAGACGTGCATGTGATGAAAGCGCGATATGTGGAAGAGCTTGCAGATGTTGTTGGTGAT  2100
Schil_Gh13_RDR      TGGGCGTGTTTTGGTCTACAGAAATCCTGGTCTTCACTTTGGAGACGTGCATGTGATGAAAGCGCGATATGTGGAAGAGCTTGCAGATGTTGTTGGTGAT  1879
Schil_BTI-87_RDR    TGGGCGTGTTTTGGTCTACAGAAATCCTGGTCTTCACTTTGGAGATGTGCATGTGATGAAAGCGCGATATGTGGAAGAGCTTGCAGATGTTGTTGGTGAT  2088
Schil_LA1932_RDR    TGGGCGTGTTTTGGTCTACAGAAATCCTGGTCTTCACTTTGGAGATGTGCATGTGATGAAAGCGCGATATGTGGAAGAGCTTGCAGATGTTGTTGGTGAT  2100
Schil_LA1938_RDR    TGGGCGTGTTTTGGTCTACAGAAATCCTGGTCTTCACTTTGGAGACGTGCATGTGATGAAAGCGCGATATGTGGAAGAGCTTGCAGATGTTGTTGGTGAT  2100
Schil_LA1971_RDR    TGGGCGTGTTTTGGTCTACAGAAATCCTGGTCTTCACTTTGGAGATGTGCATGTGATGAAAGCGCGATATGTGGAAGAGCTTGCAGATGTTGTTGGTGAT  2100
Schil_G1_1556_RDR   TGGGCGTGTTTTGGTCTACAGAAATCCTGGTCTTCACTTTGGAGATGTACATGTGATGAAAGCGCGATATGTGGAAGAGCTTGCAGATGTTGTTGGTGAT  2100
Schil_G1_1558_RDR   TGGGCGTGTTTTGGTCTACAGAAATCCTGGTCTTCACTTTGGAGATGTACATGTGATGAAAGCGCGATATGTGGAAGAGCTTGCAGATGTTGTTGGTGAT  2100
```

```
                                                                                   Catalytic domain
XM_010323869          GCCAAATATGGTATATTTTTTTCAACTAAAGGCCCGAGGTCAGCTGCTACTGAGATTGCAAATGGTGACTTTGATGGTGATATGTATTGGGTTTCCATAA   2188
Slyc_MM_RDR           GCCAAATATGGTATATTTTTTTCAACTAAAGGCCCGAGGTCAGCTGCTACTGAGATTGCAAATGGTGACTTTGATGGTGATATGTATTGGGTTTCCATAA   2188
Slyc_M82_RDR          GCCAAATATGGTATATTTTTTTCAACTAAAGGCCCGAGGTCAGCTGCTACTGAGATTGCAAATGGTGACTTTGATGGTGATATGTATTGGGTTTCCATAA   2188
Spimp_LA1589_RDR      GCCAAATATGGTATATTTTTTTCAACTAAAGGCCCGAGGTCAGCTGCTACTGAGATTGCAAATGGTGACTTTGATGGTGATATGTATTGGGTTTCCATAA   2188
Sarc_LA2157_RDR       GCCAAATATGGTATATTTTTTTCAACTAAAGGCCCGAGGTCAGCTGCTACGGAGATTGCAAATGGTGACTTTGATGGTGATATGTATTGGGTTTCCATAA   2200
Spenn_LA716_RDR       GCCAAATATGGTATATTTTTTTCAACTAAAGGCCCGAGGTGAGCTGCTACTGAGATTGCAAATGGTGACTTTGATGGTGATATGTATTGGGTTTCCATAA   2200
Shabr_LYC4_RDR        GCCAAATATGGTATATTTTTTTCAACTAAAGGCCCGAGGTCAGCTGCTACTGAGATTGCAAATGGTGACTTTGATGGTGATATGTATTGGGTTTCCATTA   2143
Sper_RDR              GCCAAATATGGTATATTTTTTTCAACTAAAGGCCCGAGGTCAGCTGCTACTGAGATTGCAAATGGTGACTTTGATGGTGATATGTATTGGGTTTCCATAA   2200
Schil_LA1969_Ty1      GCCAAATATGGTATATTTTTTTCAACTAAAGGCCCGAGGTCAGCTGCTACTGAGATTGCAAATGGTGACTTTGATGGTGATATGTATTGGGTTTCCATAA   2200
Schil_LA2779_Ty3      GCCAAATATGGTATATTTTTTTCAACTAAAGGCCCGAGGTCAGCTGCTACTGAGATTGCAAATGGTGACTTTGATGGTGATATGTATTGGGTTTCCATAA   2200
Schil_Gh13_RDR        GCCAAATATGGTATATTTTTTTCAACTAAAGGCCCGAGGTCAGCTGCTACTGAGATTGCAAATGGTGACTTTGATGGTGATATGTATTGGGTTTCCATAA   1979
Schil_BTI-87_RDR      GCCAAATATGGTATATTTTTTTCAACTAAAGGCCCGAGGTCAGCTGCTACTGAGATTGCAAATGGTGACTTTGATGGTGATATGTATTGGGTTTCCATAA   2188
Schil_LA1932_RDR      GCCAAATATGGTATATTTTTTTCAACTAAAGGCCCGAGGTCAGCTGCTACTGAGATTGCAAATGGTGACTTTGATGGTGATATGTATTGGGTTTCCATAA   2200
Schil_LA1938_RDR      GCCAAATATGGTATATTTTTTTCAACTAAAGGCCCGAGGTCAGCTGCTACTGAGATTGCAAATGGTGACTTTGATGGTGATATGTATTGGGTTTCCATAA   2200
Schil_LA1971_RDR      GCCAAATATGGTATATTTTTTTCAACTAAAGGCCCGAGGTCAGCTGCTACTGAGATTGCAAATGGTGACTTTGATGGTGATATGTATTGGGTTTCCATAA   2200
Schil_G1_1556_RDR     GCCAAATATGGTATATTTTTTTCAACTAAAGGCCCGAGGTCAGCTGCTACTGAGATTGCAAATGGTGACTTTGATGGTGATATGTATTGGGTTTCCATAA   2200
Schil_G1_1558_RDR     GCCAAATATGGTATATTTTTTTCAACTAAAGGCCCGAGGTCAGCTGCTACTGAGATTGCAAATGGTGACTTTGATGGTGATATGTATTGGGTTTCCATAA   2200

                              exon 15
XM_010323869          ACCGTAAGTTGGTAGATTCTTATACAACAAGTAGACCATGGATTCGCATGCATTCAACTCCTAACGCAGTTAGCAAAAAACCAAGTGAATTTTCAGCTGA   2288
Slyc_MM_RDR           ACCGTAAGTTGGTAGATTCTTATACAACAAGTAGACCATGGATTCGCATGCATTCAACTCCTAACGCAGTTAGCAAAAAACCAAGTGAATTTTCAGCTGA   2288
Slyc_M82_RDR          ACCGTAAGTTGGTAGATTCTTATACAACAAGTAGACCATGGATTCGCATGCATTCAACTCCTAAGGCAGTTAGCAAAAAACCAAGTGAATTTTCAGCTGA   2288
Spimp_LA1589_RDR      ACCGTAAGTTGGTAGATTCTTATACAACAAGTAGACCATGGATTCGCATGCATTCAACTCCTAAGGCAGTTAGCAAAAAACCAAGTGAATTTTCAGCTGA   2288
Sarc_LA2157_RDR       ACCGTAAGGTGGTAGATTCTTATACAACAAGTAGACCATGGATTCGCATGCATTCAACTCCTAAGGCAGTTAGCAAAAAACCAAGTGAATTTTCAGCTGA   2300
Spenn_LA716_RDR       ACCGTAAGGTGGTAGATTCTTATACAACAAGTAGACCATGGATTCGCATGCATTCAACTCCTAAGGCAGTTAGCAAAAAACCAAGTGAATTTTCAGCTGA   2300
Shabr_LYC4_RDR        ACCGTAAGGTGGTAGATTCTTATACAACAAGTAGACCATGGATTCGCATGCATTCAACTCCTAAGGCAGTTAGCAAAAAACCAAGTGAATTTTCAGCTGA   2243
Sper_RDR              ACCGTAAGGTGGTAGATTCTTATACAACAAGTAGACCATGGATTCGCATGCATTCAACTCCTAAGGCAGTTAGCAAAAAACCAAGTGAATTTTCAGCTGA   2300
Schil_LA1969_Ty1      ACCGTAAGTTGGTAGATTCTTATACAACAAGTAGACCATGGATTCGCATGCATTCAACTCCTAAGGCAGTTAGCAAAAAACCAAGTGAATTTTCAGCTGA   2300
Schil_LA2779_Ty3      ACCGTAAGTTGGTAGATTCTTATACAACAAGTAGACCATGGATTCGCATGCATTCAACTCCTAAGGCAGTTAGCAAAAAACCAAGTGAATTTTCAGCTGA   2300
Schil_Gh13_RDR        ACCGTAAGTTGGTAGATTCTTATACAACAAGTAGACCATGGATTCGCATGCATTCAACTCCTAAGGCAGTTAGCAAAAAACCAAGTGAATTTTCAGCTGA   2079
Schil_BTI-87_RDR      ACCGTAAGTTGGTAGATTCTTATACAACAAGTAGACCATGGATTCGCATGCATTCAACTCCTAAGGCAGTTAGCAAAAAACCAAGTGAATTTTCAGCTGA   2288
Schil_LA1932_RDR      ACCGTAAGTTGGTAGATTCTTATACAACAAGTAGACCATGGATTCGCATTCATTCAACTCCTAAGGCAGTTAGCAAAAAACCAAGTGAATTTTCAGCTGA   2300
Schil_LA1938_RDR      ACCGTAAGTTGGTAGATTCTTATACAACAAGTAGACCATGGATTCGCATGCATTCAACTCCTAAGGCAGTTAGCAAAAAGCCAAGTGAATTTTCAGCTGA   2300
Schil_LA1971_RDR      ACCGTAAGTTGGTAGATTCTTATACAACAAGTAGACCATGGATTCGCATGCATTCAACTCCTAAGGCAGTTAGCAAAAAACCAAGTGAATTTTCAGCTGA   2300
Schil_G1_1556_RDR     ACCGTAAGGTGGTAGATTCTTATACAACAAGTAGACCATGGATTCGCATGCATTCAACTCCTAAGGCAGTTAGCAAAAAACCAAGTGAATTTTCAGCTGA   2300
Schil_G1_1558_RDR     ACCGTAAGGTGGTAGATTCTTATACAACAAGTAGACCATGGATTCGCATGCATTCAACTCCTAAGGCAGTTAGCAAAAAACCAAGTGAATTTTCAGCTGA   2300

                                                                                exon 16
XM_010323869          TGAATTGGAATATGAGCTTTTTAGGCAATTTCTGGAAGCAAAGTCTAAAGGTGCCAATATGTCTCTGGCAGCTGATAGCTGGCTGGCATTTATGGATCGT   2388
Slyc_MM_RDR           TGAATTGGAATATGAGCTTTTTAGGCAATTTCTGGAAGCAAAGTCTAAAGGTGCCAATATGTCTCTGGCAGCTGATAGCTGGCTGGCATTTATGGATCGT   2388
Slyc_M82_RDR          TGAATTGGAATATGAGCTTTTTAGGCAATTTCTGGAAGCAAAGTCTAAAGGTGCCAATATGTCTCTGGCAGCTGATAGCTGGCTGGCATTTATGGATCGT   2388
Spimp_LA1589_RDR      TGAATTGGAATATGAGCTTTTTAGGCAATTTCTGGAAGCAAAGTCTAAAGGTGCCAATATGTCTCTGGCAGCTGATAGCTGGCTGGCATTTATGGATCAT   2388
Sarc_LA2157_RDR       TGAATTGGAATATGAGCTTTTTAGGCAATTTCTGGAAGCAAAGTCTAAAGGTGCCAATATGTCTCTGGCAGCTGATAGCTGGCTGGCATTTATGGATCGT   2400
Spenn_LA716_RDR       TGAATTGGAATATGAGCTTTTTAGGCAATTTCTGGAAGCAAAGTCTAAAGGTGCCAATATGTCTCTGGCAGCTGATAGCTGGCTGGCATTTATGGATCGT   2400
Shabr_LYC4_RDR        TGAATTGGAATATGAGCTTTTTAGGCAATTTCTGGAAGCAAAGTCTAAAGGTGCCAATATGTCTCTGGCAGCTGATAGCTGGCTGGCATTTATGGATCGT   2343
Sper_RDR              TGAATTGGAATATGAGCTTTTTAGGCAATTTCTGGAAGCAAAGTCTAAAGGTGCCAATATGTCTCTGGCAGCTGATAGCTGGCTGGCATTTATGGATCGT   2400
Schil_LA1969_Ty1      TGAATTGGAATATGAGCTTTTCAGGCAATTTCTGGAAGCAAAGTCTAAAGGTGCCAATATGTCTCTGGCAGCTGATAGCTGGCTGGCATTTATGGATCGT   2400
Schil_LA2779_Ty3      TGAATTGGAATATGAGCTTTTTAGGCAATTTCTGGAAGCAAAGTCTAAAGGTGCCAATATGTCTCTGGCAGCTGATAGCTGGCTGGCATTTATGGATCGT   2400
Schil_Gh13_RDR        TGAATTGGAATATGAGCTTTTTAGGCAATTTCTGGAAGCAAAGTCTAAAGGTGCCAATATGTCTCTGGCAGCTGATAGCTGGCTGGCATTTATGGATCGT   2179
Schil_BTI-87_RDR      TGAATTGGAATATGAGCTTTTTAGGCAATTTCTGGAAGCAAAGTCTAAAGGTGCCAATATGTCTCTGGCAGCTGATAGCTGGCTGGCATTTATGGATCGT   2388
Schil_LA1932_RDR      TGAATTGGAATATGAGCTTTTTAGGCAATTTCTGGAAGCAAAGTCTAAAGGTGCCAATATGTCTCTGGCAGCTGATAGCTGGCTGGCATTTATGGATCGT   2400
Schil_LA1938_RDR      TGAATTGGAATATGAGCTTTTTAGGCAATTTCTGGAAGCAAAGTCTAAAGGTGCCAATATGTCTCTGGCAGCTGATAGCTGGCTGGCATTTATGGATCGT   2400
Schil_LA1971_RDR      TGAATTGGAATATGAGCTTTTTAGGCAATTTCTGGAAGCAAAGTCTAAAGGTGCCAATATGTCTCTGGCAGCTGATAGCTGGCTGGCATTTATGGATCGT   2400
Schil_G1_1556_RDR     TGAATTGGAATATGAGCTTTTTAGGCAATTTCTGGAAGCAAAGTCTAAAGGTGCCAATATGTCTCTGGCAGCTGATAGCTGGCTGGCATTTATGGATCGT   2400
Schil_G1_1558_RDR     TGAATTGGAATATGAGCTTTTTAGGCAATTTCTGGAAGCAAAGTCTAAAGGTGCCAATATGTCTCTGGCAGCTGATAGCTGGCTGGCATTTATGGATCGT   2400
```

```
XM_010323869       CTTCTGACGCTGCGAGATGATAATGTGGATGATATGCATAGCTTGAAAGGCAAGATGCTTCACCTGATTGACATCTACTATGATGCATTAGATGCACCTA  2488
Slyc_MM_RDR        CTTCTGACGCTGCGAGATGATAATGTGGATGATATGCATAGCTTGAAAGGCAAGATGCTTCACCTGATTGACATCTACTATGATGCATTAGATGCACCTA  2488
Slyc_M82_RDR       CTTCTGACGCTGCGAGATGATAATGTGGATGATATGCATAGCTTGAAAGGCAAGATGCTTCACCTGATTGACATCTACTATGATGCATTAGATGCACCTA  2488
Spimp_LA1589_RDR   CTTCTGACGCTGCGAGATGATAATGTGGATGATATGCATAGCTTGAAAGGCAAGATGCTTCACCTGATTGACATCTACTATGATGCATTAGATGCACCTA  2488
Sarc_LA2157_RDR    CTTCTGACGCTGCGAGATGATAATGTGGATGATATGCATAGCTTGAAAGGCAAGATGCTTCACCTGATTGACATCTACTATGATGCATTAGATGCACCTA  2500
Spenn_LA716_RDR    CTTCTGATGCTGCGAGATGATAATGTGGATGATATGCATAGCTTGAAAGGCAAGATGCTTCACCTGATTGACATCTACTATGATGCATTAGATGCACCTA  2500
Shabr_LYC4_RDR     CTTCTGATGCTGCGAGATGATAATGTGGATGATATGCATAGCTTGAAAGGCAAGATGCTTCACCTGATTGACATCTACTATGATGCATTAGATGCACCTA  2443
Sper_RDR           CTTCTGATGCTGCGAGATGATAATGTGGATGATATGCATAGCTTGAAAGGCAAGATGCTTCACCTGATTGACATCTACTATGATGCATTAGATGCACCTA  2500
Schil_LA1969_Ty1   CTTCTGATGCTGCGAGATGATAATGTGGATGATATGCATAGCTTGAAAGGCAAGATGCTTCACCTGATTGACATCTACTATGATGCATTAGATGCACCTA  2500
Schil_LA2779_Ty3   CTTCTGATGCTGCGAGATGATAATGTGGATGATATGCATAGCTTGAAAGGCAAGATGCTTCACCTGATTGACATCTACTATGATGCATTAGATGCACCTA  2500
Schil_Gh13_RDR     CTTCTGATGCTGCGAGATGATAATGTGGATGATATGCATAGCTTGAAAGGCAAGATGCTTCACCTGATTGACATCTACTATGATGCATTAGATGCACCTA  2279
Schil_BTI-87_RDR   CTTCTGATGCTGCGAGATGATAATGTGGATGATATGCATAGATTGAAAGGCAAGATGCTTCACTTGATTGACATCTACTATGATGCATTAGATGCACCTA  2488
Schil_LA1932_RDR   CTTCTGATGCTGCGAGATGATAATGTGGATGATATGCATAGCTTGAAAGGCAAGATGCTTCACTTGATTGACATCTACTATGATGCATTAGATGCACCTA  2500
Schil_LA1938_RDR   CTTCTGATGCTGCGAGATGATAATGTGGATGATATGCATAGCTTGAAAGGCAAGATGCTTCACCTGATTGACATCTACTATGATGCATTAGATGCACCTA  2500
Schil_LA1971_RDR   CTTCTGATGCTGCGAGATGATAATGTGGATGATATGCATAGCTTGAAAGGCAAGATGCTTCACCTGATTGACATCTACTATGATGCATTAGATGCACCTA  2500
Schil_G1_1556_RDR  CTTCTGACGCTGCGAGATGATAATGTGGATGATATGCATAGCTTGAAAGGCAAGATGCTTCACCTGATTGACATCTACTATGATGCATTAGATGCACCTA  2500
Schil_G1_1558_RDR  CTTCTGACGCTGCGAGATGATAATGTGGATGATATGCATAGCTTGAAAGGCAAGATGCTTCACCTGATTGACATCTACTATGATGCATTAGATGCACCTA  2500

                                 exon 17
XM_010323869       AAAGCGGGAAGAAGGTTAGCATCCCTCATTATCTGAAGGCAAACAAGTTCCCCCACTATATGGAAAAAGGGAACTCCTGCAGCTATCATTCAACTTCTAT  2588
Slyc_MM_RDR        AAAGCGGGAAGAAGGTTAGCATCCCTCATTATCTGAAGGCAAACAAGTTCCCCCACTATATGGAAAAAGGGAACTCCTGCAGCTATCATTCAACTTCTAT  2588
Slyc_M82_RDR       AAAGCGGGAAGAAGGTTAGCATCCCTCATTATCTGAAGGCAAACAAGTTCCCCCACTATATGGAAAAAGGGAACTCCTGCAGCTATCATTCAACTTCTAT  2588
Spimp_LA1589_RDR   AAAGCGGGAAGAAGGTTAGCATCCCTCATTATCTGAAGGCAAACAAGTTCCCCCACTATATGGAAAAAGGGAACTCCTGCAGCTATCATTCAACTTCTAT  2588
Sarc_LA2157_RDR    AAAGCGGGAAGAAGGTTAGCATCCCTCATTATCTGAAGGCAAACAAGTTCCCCCACTATATGGAAAAAGGGAACTCCTGCAGCTATCATTCAACTTCTAT  2600
Spenn_LA716_RDR    AAAGCGGGAAGAAGGTTAGCATCCCTCATTATCTGAAGGCAAACAAGTTCCCCCACTATATGGAAAAAGGGAACTCCTGCAGCTATCATTCAACTTCTAT  2600
Shabr_LYC4_RDR     AAAGCGGGAAGAAGGTTAGCATCCCTCATTATCTGAAGGCAAACAAGTTCCCCCACTATATGGAAAAAGGGAACTCCTGCAGCTATCATTCAACTTCTAT  2543
Sper_RDR           AAAGCGGGAAGAAGGTTAGCATCCCTCATTATCTGAAGGCAAACAAGTTCCCCCACTATATGGAAAAAGGGAACTCCTGCAGCTATCATTCAACTTCTAT  2600
Schil_LA1969_Ty1   AAAGCGGGAAGAAGGTTAGCATCCCTCATTATCTGAAGGCAAACAAGTTCCCCCACTATATGGAAAAAGGGAACTCCTGCAGCTATCATTCAACTTCTAT  2600
Schil_LA2779_Ty3   AAAGCGGGAAGAAGGTTAGCATCCCTCATTATCTGAAAGCAAACAAGTTCCCCCACTATATGGAAAAAGGGAACTCCTGCAGCTATCATTCAACTTCTAT  2600
Schil_Gh13_RDR     AAAGCGGGAAGAAGGTTAGCATCCCTCATTATCTGAAAGCAAACAAGTTCCCCCACTATATGGAAAAAGGGAACTCCTGCAGCTATCATTCAACTTCTAT  2379
Schil_BTI-87_RDR   AAAGCGGGAAGAAGGTTAGCATCCCTCATTATCTGAAGGCAAACAAGTTCCCCCACTATATGGAAAAAGGGAACTCCTGCAGCTATCATTCAACTTCTAT  2588
Schil_LA1932_RDR   AAAGCGGGAAGAAGGTTAGCATCCCTCATTATCTGAAGGCAAACAAGTTCCCCCACTATATGGAAAAAGGGAACTCCTGCAGCTATCATTCAACTTCTAT  2600
Schil_LA1938_RDR   AAAGCGGGAAGAAGGTTAGCATCCCTCATTATCTGAAAGCAAACAAGTTCCCCCACTATATGGAAAAAGGGAACTCCTGCAGCTATCATTCAACTTCTAT  2600
Schil_LA1971_RDR   AAAGCGGGAAGAAGGTTAGCATCCCTCATTATCTGAAGGCAAACAAGTTCCCCCACTATATGGAAAAAGGGAACTCCTGCAGCTATCATTCAACTTCTAT  2600
Schil_G1_1556_RDR  AAAGCGGGAAGAAGGTTAGCATCCCTCATTATCTGAAGGCAAACAAGTTCCCCCACTATATGGAAAAAGGGAACTCCTGCAGCTATCATTCAACTTCTAT  2600
Schil_G1_1558_RDR  AAAGCGGGAAGAAGGTTAGCATCCCTCATTATCTGAAGGCAAACAAGTTCCCCCACTATATGGAAAAAGGGAACTCCTGCAGCTATCATTCAACTTCTAT  2600

                                                                                 intron 17
XM_010323869       TCTGGGTCAGATTTATGATCATGTCGACTCATATCCAGATGAAGATTTGTGTATAACAG-----------------------------------------  2647
Slyc_MM_RDR        TCTGGGTCAGATTTATGATCATGTCGACTCATATCCAGATGAAGATTTGTGTATAACAG-----------------------------------------  2647
Slyc_M82_RDR       TCTGGGTCAGATTTATGATCATGTCGACTCATATCCAGATGAAGATTTGTGTATAACAG-----------------------------------------  2647
Spimp_LA1589_RDR   TCTGGGTCAGATTTATGATCATGTCGACTCATATCCAGATGAAGATTTGTGTATAACAG-----------------------------------------  2647
Sarc_LA2157_RDR    TCTGGGTCAGATTTATGATCATGTCGACTCATATCCAGATGAAGATTTGTGTATAACAG-----------------------------------------  2659
Spenn_LA716_RDR    TCTGGGTCAGATTTATGATCATGTCGACTCATATCCAGATGAAGATTTGTGTATAACAG-----------------------------------------  2659
Shabr_LYC4_RDR     TCTGGGTCAGATTTATGATCATGTCGACTCATATCCAGATGAAGATTTGTGTATAACAG-----------------------------------------  2602
Sper_RDR           TCTGGGTCAGATTTATGATCATGTCGACTCATATCCAGATGAAGATTTGTGTATAACAG-----------------------------------------  2659
Schil_LA1969_Ty1   TCTGGGTCAGATTTATGATCATGTCGACTCATATCCAGATGAAGATTTGTGTATAACAG-----------------------------------------  2659
Schil_LA2779_Ty3   TCTGGGTCAGATTTATGATCATGTCGACTCATATCCAGATGAAGATTTGTGTATAACAG-----------------------------------------  2659
Schil_Gh13_RDR     TCTGGGTCAGATTTATGATCATGTCGACTCATATCCAGATGAAGATTTGTGTATAACAG-----------------------------------------  2438
Schil_BTI-87_RDR   TCTGGGTCAGATTTATGATCATGTCGACTCATATCCAGATGAAGATTTGTGCATAACAG-----------------------------------------  2647
Schil_LA1932_RDR   TCTGGGTCAGATTTATGATCATGTCGACTCATATCCAGATGAAGATTTGTGCATAACAG-----------------------------------------  2659
Schil_LA1938_RDR   TCTGGGTCAGATTTATGATCATGTCGACTCATATCCAGATGAAGATTTGTGTATAACAG-----------------------------------------  2659
Schil_LA1971_RDR   TCTGGGTCAGATTTATGATCATGTCGACTCATATCCAGATGAAGATTTGTGTATAACAG-----------------------------------------  2659
Schil_G1_1556_RDR  TCTGGGTCAGATTTATGATCATGTCGACTCATATCCAGATGAAGATTTGTGTATAACAGGTTAAAGCACCCCTCTTGCATGCATAAGATCTGGGCTGGTG  2700
Schil_G1_1558_RDR  TCTGGGTCAGATTTATGATCATGTCGACTCATATCCAGATGAAGATTTGTGTATAACAG-----------------------------------------  2659
```

```
XM_010323869          ---------------------------------------------------------------------------------------------------- 2647
Slyc_MM_RDR           ---------------------------------------------------------------------------------------------------- 2647
Slyc_M82_RDR          ---------------------------------------------------------------------------------------------------- 2647
Spimp_LA1589_RDR      ---------------------------------------------------------------------------------------------------- 2647
Sarc_LA2157_RDR       ---------------------------------------------------------------------------------------------------- 2659
Spenn_LA716_RDR       ---------------------------------------------------------------------------------------------------- 2659
Shabr_LYC4_RDR        ---------------------------------------------------------------------------------------------------- 2602
Sper_RDR              ---------------------------------------------------------------------------------------------------- 2659
Schil_LA1969_Ty1      ---------------------------------------------------------------------------------------------------- 2659
Schil_LA2779_Ty3      ---------------------------------------------------------------------------------------------------- 2659
Schil_Gh13_RDR        ---------------------------------------------------------------------------------------------------- 2438
Schil_BTI-87_RDR      ---------------------------------------------------------------------------------------------------- 2647
Schil_LA1932_RDR      ---------------------------------------------------------------------------------------------------- 2659
Schil_LA1938_RDR      ---------------------------------------------------------------------------------------------------- 2659
Schil_LA1971_RDR      ---------------------------------------------------------------------------------------------------- 2659
Schil_G1_1556_RDR     TTTCCAACGAAGCCCCATTGCTGCATAGTGTTTGAGCTTATGTTTTCCTATTGCATATATGAAGATTTGATGATTCGATTGAAAATTATAGCAGTTGAGT 2800
Schil_G1_1558_RDR     ---------------------------------------------------------------------------------------------------- 2659

                                                     exon 18
XM_010323869          -------------------------------AAATCTCTAAACTGCCTTGCTTTGAAGTTGAAATCCCTCAAAGATGCATGACATTGTGGAGAGGAAGAT 2716
Slyc_MM_RDR           -------------------------------AAATCTCTAAACTGCCTTGCTTTGAAGTTGAAATCCCTCAAAGATGCATGACATTGTGGAGAGGAAGAT 2716
Slyc_M82_RDR          -------------------------------AAATCTCTAAACTGCCTTGCTTTGAAGTTGAAATCCCTCAAAGATGCATGACATTGTGGAGAGGAAGAT 2716
Spimp_LA1589_RDR      -------------------------------AAATCTCTAAACTGCCTTGCTTTGAAGTTGAAATCCCTCAAAGATGCATGACATTGTGGAGAGGAAGAT 2716
Sarc_LA2157_RDR       -------------------------------AGATCTCTAAACTTCCTTGCTTTGAAGTTGAAATCCCTCAAAGATGCATGACATTGTGGAGAGGAAGAT 2728
Spenn_LA716_RDR       -------------------------------AGATCTCTAAACTGCCTTGCTTTGAAGTTGAAATCCCTCAAAGATGCATGACATTGTGGAGAGGAAGAT 2728
Shabr_LYC4_RDR        -------------------------------AGATCTCTAAACTCCCTTGCTTTGAAGTTGAAATCCCTCAAAGATGCATGACATTGTGGAGAGGAAGAT 2671
Sper_RDR              -------------------------------AGATCTCTAAACTGCCTTGCTTTGAAGTTGAAATCCCTCAAAGATGCATGACATTGTGGAGAGGAAGAT 2728
Schil_LA1969_Ty1      -------------------------------AGATCTCTAAACTGCCTTGCTTTGAAGTTGAAATCCCTCAAAGATGCATGACATTGTGGAGAGGAAGAT 2728
Schil_LA2779_Ty3      -------------------------------AGATCTCTAAACTGCCTTGCTTTGAAGTTGAAATCCCTCAAAGATGCATGACATTGTGGAGAGGAAGAT 2728
Schil_Gh13_RDR        -------------------------------AGATCTCTAAACTGCCTTGCTTTGAAGTTGAAATCCCTCAAAGATGCATGACATTGTGGAGAGGAAGAT 2507
Schil_BTI-87_RDR      -------------------------------AGATCTCTAAACTGCCTTGCTTTGAAGTTGAAATCCCTCAAAGATGCATGACATTGTGGAGAGGAAGAT 2716
Schil_LA1932_RDR      -------------------------------AGATCTCTAAACTGCCTTGCTTTGAAGTTGAAATCCCTCAAAGATGCATGACATTGTGGAGAGGAAGAT 2728
Schil_LA1938_RDR      -------------------------------AGATCTCTAAACTGCCTTGCTTTGAAGTTGAAATCCCTCAAAGATGCATGACATTGTGGAGAGGAAGAT 2728
Schil_LA1971_RDR      -------------------------------AGATCTCTAAACTGCCTTGCTTTGAAGTTGAAATCCCTCAAAGATGCATGACATTGTGGAGAGGAAGAT 2728
Schil_G1_1556_RDR     GATGATTGTGTTGCTTCTTTGTTGTGTGTAGAGATCTCTAAACTGCCTTGCTTTGAAGTTGAAATCCCTCAAAGATGCATGACATTGTGGAGAGGAAGAT 2900
Schil_G1_1558_RDR     -------------------------------AGATCTCTAAACTGCCTTGCTTTGAAGTTGAAATCCCTCAAAGATGCATGACATTGTGGAGAGGAAGAT 2728

                                                                                                                       exon 19
XM_010323869          ATGAAGAGTACAAAAAGGATATGACACGGGCCATGAACTTTGATTGTGAACTAAGAATCACCTCTTGCAATGAAGTTATAAAGAAGTACAAGATGTTGCT 2816
Slyc_MM_RDR           ATGAAGAGTACAAAAAGGATATGACACGGGCCATGAACTTTGATTGTGAACTAAGAATCACCTCTTGCAATGAAGTTATAAAGAAGTACAAGATGTTGCT 2816
Slyc_M82_RDR          ATGAAGAGTACAAAAAGGATATGACACGGGCCATGAACTTTGATTGTGAACTAAGAATCACCTCTTGCAATGAAGTTATAAAGAAGTACAAGATGTTGCT 2816
Spimp_LA1589_RDR      ATGAAGAGTACAAAAAGGATATGACACGGGCCATGAACTTTGATTGTGAACTAAGAATCACCTCTTGCAATGAAGTTATAAAGAAGTACAAGATGTTGCT 2816
Sarc_LA2157_RDR       ATGAAGAGTACAAAAAGGATATGACACGGGCCATGAACTTAGATTGTGAACTTAGAATCACCTCTTGCAATGAAGTTATAAAGAAGTACAAGATGTTGCT 2828
Spenn_LA716_RDR       ATGAAGAGTACAAAAAGGATATGACACGGGCCATGAACTTAGATTGTGAACTTAGAATCACCTCTTGCAATGAAGTTATAAAGAAGTACAAGATGTTGCT 2828
Shabr_LYC4_RDR        ATGAAGAGTACAAAAAGGATATGACACGGGCCATGAACTTAGATTGTGAACTTAGAATCACCTCTTGCAATGAAGTTATAAAGAAGTACAAGATGTTGCT 2771
Sper_RDR              ATGAAGAGTACAAAAAGGATATGACACGGGCCATGAACTTAGATTGTGAACTTAGAATCACCTCTTGCAATGAAGTTATAAAGAAGTACAAGATGTTGCT 2828
Schil_LA1969_Ty1      ATGAAGAGTACAAAAAGGATATGACACAGGCCATGAACTTAGATTGTGAACTTAGAATCACCTCTTGCAATGAAGTTATAAAGAAGTACAAGATGTTGCT 2828
Schil_LA2779_Ty3      ATGAAGAGTACAAAAAGGATATGACACAGGCCATGAACTTAGATTGTGAACTTAGAATCACCTCTTGCAATGAAGTTATAAAGAAGTACAAGATGTTGCT 2828
Schil_Gh13_RDR        ATGAAGAGTACAAAAAGGATATGACACAGGCCATGAACTTAGATTGTGAACTTAGAATCACCTCTTGCAATGAAGTTATAAAGAAGTACAAGATGTTGCT 2607
Schil_BTI-87_RDR      ATGAAGAGTACAAAAAGGATATGACACAGGCCATGAACTTAGATTGTGAACTTAGAATCACCTCTTGCAATGAAGTTATAAAGAAGTACAAGATGTTGCT 2816
Schil_LA1932_RDR      ATGAAGAGTACAAAAAGGATATGACACAGGCCATGAACTTAGATTGTGAACTTAGAATCACCTCTTGCAATGAAGTTATAAAGAAGTACAAGATGTTGCT 2828
Schil_LA1938_RDR      ATGAAGAGTACAAAAAGGATATGACACAGGCCATGAACTTAGATTGTGAACTTAGAATCACCTCTTGCAATGAAGTTATAAAGAAGTACAAGATGTTGCT 2828
Schil_LA1971_RDR      ATGAAGAGTACAAAAAGGATATGACACAGGCCATGAACTTAGATTGTGAACTTAGAATCACCTCCTGCAATGAAGTTATAAAGAAGTACAAGATGTTGCT 2828
Schil_G1_1556_RDR     ATGAAGAGTACAAAAAGGATATGACACGGGCCATGAACTTAGATTGTGAACTTAGAATCACCTCTTGCAATGAAGTTATAAAGAAGTACAAGATGTTGCT 3000
Schil_G1_1558_RDR     ATGAAGAGTACAAAAAGGATATGACACGGGCCATGAACTTAGATTGTGAACTTAGAATCACCTCTTGCAATGAAGTTATAAAGAAGTACAAGATGTTGCT 2828
```

XM_010323869	ATATGGTGCTGTGGAGTTTGAACAACAGTAAGAAAGACTGAAGACATTTTCGACGAGGCCCTTGCAATATATCATGTAACATATGATAATGCAAGGATC	2916
Slyc_MM_RDR	ATATGGTGCTGTGGAGTTTGAACAACAGTAAGAAAGACTGAAGACATTTTCGACGAGGCCCTTGCAATATATCATGTAACATATGATAATGCAAGGATC	2916
Slyc_M82_RDR	ATATGGTGCTGTGGAGTTTGAACAACAGTAAGAAAGACTGAAGACATTTTCGACGAGGCCCTTGCAATATATCATGTAACATATGATAATGCAAGGATC	2916
Spimp_LA1589_RDR	ATATGGTGCTGTGGAGTTTGAACAACAGTAAGAAAGACTGAAGACATTTTCGACGAGGCCCTTGCAATATATCATGTAACATATGATAATGCAAGGATC	2916
Sarc_LA2157_RDR	ATATGGTGCTGTGGAGTTTGAACAACAGTAAGAAAGACTGAAGACATTTTCGACGAGGCCCTTGCAATATATCATGTAACATATGATAATGCAAGGATC	2928
Spenn_LA716_RDR	ATATGGTGCTGTGGAGTTTGAACAACAGTAAGAAAGACTGAAGACATTTTCGATGAGGCCCTTGCAATATATCATGTAACATATGATAATGCAAGGATC	2928
Shabr_LYC4_RDR	ATATGGTGCTGTGGAGTTTGAACAACAGTAAGAAAGACTGAAGACATTTTCGATGTGGCCCTTGCAATATATCATGTAACATATGATAATGCAAGGATC	2871
Sper_RDR	ATATGGTGCTGTGGAGTTTGAACAACAGTAAGAAAGACTGAAGACATTTTCGATGAGGCCCTTGCAATATATCATGTAACATATGATAATGCAAGGATC	2928
Schil_LA1969_Ty1	ATATGGTGCTGTGGAGTTTGAACAACAGTAAGAAAGACTGAAGACATTTTCGATGAAGCCCTTGCAATATATCATGTAACATATGATAATGCAAGGATC	2928
Schil_LA2779_Ty3	ATATGGTGCTGTGGAGTTTGAACAACAGTAAGAAAGACTGAAGACATTTTCGATGAAGCCCTTGCAATATATCATGTAACATATGATAATGCAAGGATC	2928
Schil_Gh13_RDR	ATATGGTGCTGTGGAGTTTGAACAACAGTAAGAAAGACTGAAGACATTTTCGATGAAGCCCTTGCAATATATCATGTAACATATGATAATGCAAGGATC	2707
Schil_BTI-87_RDR	ATATGGTGCTGTGGAGTTTGAACAACAGTAAGAAAGACTGAAGACATTTTCGATGAGGCCCTTGCAATATATCATGTAACATATGATAATGCAAGGATC	2916
Schil_LA1932_RDR	ATATGGTGCTGTGGAGTTTGAACAACAGTAAGAAAGACTGAAGACATTTTCGATGAGGCCCTTGCAATATATCATGTAACATATGATAATGCAAGGATC	2928
Schil_LA1938_RDR	ATATGGTGCTGTGGAGTTTGAACAACAGTAAGAAAGACTGAAGACATTTTCGATGAGGCCCTTGCAATATATCATGTAACATATGATAATGCAAGGATC	2928
Schil_LA1971_RDR	ATATGGTGCTGTAGAGTTTGAACAACAGTAAGAAAGACTGAAGACATTTTTGATGAGGCCCTTGCAATATATCATGTAACATATGATAATGCAAGGATC	2928
Schil_G1_1556_RDR	ATATGGTGCTGTGGAGTTTGAACAACAGTAAGAAAGACTGAAGACATTTTCGATGAGGCCCTTGCAATATATCATGTAACATATGATAATGCAAGGATC	3100
Schil_G1_1558_RDR	ATATGGTGCTGTGGAGTTTGAACAACAGTAAGAAAGACTGAAGACATTTTCGATGAGGCCCTTGCAATATATCATGTAACATATGATAATGCAAGGATC	2928

XM_010323869	ACATACAGCATAGAGAAATGTGGTTTTGCTTGGAAAGTAGCTGGTTCTGCGCTTTGCAGGATCCACGCCATGTATCGCAAGGAAAAAGACTTGCCCATTT	3016
Slyc_MM_RDR	ACATACAGCATAGAGAAATGTGGTTTTGCTTGGAAAGTAGCTGGTTCTGCGCTTTGCAGGATCCACGCCATGTATCGCAAGGAAAAAGACTTGCCCATTT	3016
Slyc_M82_RDR	ACATACAGCATAGAGAAATGTGGTTTTGCTTGGAAAGTAGCTGGCTCTGCGCTTTGCAGGATCCACGCCATGTATCGCAAGGAAAAAGACTTGCCCATTT	3016
Spimp_LA1589_RDR	ACATACAGCATAGAGAAATGTGGTTTTGCTTGGAAAGTAGCTGGCTCTGCGCTTTGCAGGATCCACGCCATGTATCGCAAGGAAAAAGACTTGCCCATTT	3016
Sarc_LA2157_RDR	ACATACAGCATAGAGAAATGTGGTTTTGCTTGGAAAGTAGCTGGTTCTGCGCTTTGCAGGATCCACGCCATGTATCGCAAGGAAAAAGACTTGCCCATTT	3028
Spenn_LA716_RDR	GCGTACAGCATAGAGAAATGTGGTTTTGCTTGGAAAGTAGCTGGTTCTGCGCTTTGCAGGATCCACGCCATGTATCGCAAGGAAAAAGACTTGCCCATTT	3028
Shabr_LYC4_RDR	GCATACAGCATAGAGAAATGTGGTTTTGCTTGGAAAGTAGCTGGTTCTGCGCTTTGCAGGATCCACGCCATGTATCGCAAGGAAAAAGACTTGCCCATTT	2971
Sper_RDR	TCATACAGCATAGAGAAATGTGGTTTTGCTTGGAAAGTAGCTGGTTCTGCGCTTTGCAGGATCCACGCCATGTATCGCAAGGAAAAAGACTTGCCCATTT	3028
Schil_LA1969_Ty1	ACATACAGCATAGAGAAATGTGGTTTTGCTTGGAAAGTAGCTGGTTCTGCGCTTTGCAGGATCCACGCCATGTATCGCAAGGAAAAAGACTTGCCCATTT	3028
Schil_LA2779_Ty3	ACATACAGCATAGAGAAATGTGGTTTTGCTTGGAAAGTAGCTGGTTCTGCGCTTTGCAGGATCCACGCCATGTATCGCAAGGAAAAAGACTTGCCCATTT	3028
Schil_Gh13_RDR	ACATACAGCATAGAGAAATGTGGTTTTGCTTGGAAAGTAGCTGGTTCTGCGCTTTGCAGGATCCACGCCATGTATCGCAAGGAAAAAGACTTGCCCATTT	2807
Schil_BTI-87_RDR	ACATACAGCATAGAGAAATGTGGTTTTGCTTGGAAAGTAGCTGGTTCTGCGCTTTGCAGGATCCACGCCATGTATCACAAGGAAAAAGACTTGCCCATTT	3016
Schil_LA1932_RDR	ACATACAGCATAGAGAAATGTGGTTTTGCTTGGAAAGTAGCTGGTTCTGCGCTTTGCAGGATCCACGCCATGTATCACAAGGAAAAAGACTTGCCCATTT	3028
Schil_LA1938_RDR	ACATACAGCATAGAGAAATGTGGTTTTGCTTGGAAAGTAGCTGGTTCTGCGCTTTGCAGGATCCACGCCATGTATCGCAAGGAAAAAGACTTGCCCATTT	3028
Schil_LA1971_RDR	GCATACAGCATAGAGAAATGTGGTTTTGCTTGGAAAGTAGCTGGTTCTGCGCTTTGCAGGATCCACGCCATGTATCGCAAGGAAAAAGACTTGCCCATTT	3028
Schil_G1_1556_RDR	TCATACAGCATAGAGAAATGTGGTTTTGCTTGGAAAGTAGCTGGTTCTGCGCTTTGCAGGATCCACGCCATGTATCGCAAGGAAAAAGACTTGCCCATTT	3200
Schil_G1_1558_RDR	TCATACAGCATAGAGAAATGTGGTTTTGCTTGGAAAGTAGCTGGTTCTGCGCTTTGCAGGATCCACGCCATGTATCGCAAGGAAAAAGACTTGCCCATTT	3028

XM_010323869	TGCCATCGGTTTTGCAGGAAATACTCTAG	3045
Slyc_MM_RDR	TGCCATCGGTTTTGCAGGAAATACTCTAG	3045
Slyc_M82_RDR	TGCCATCGGTTTTGCAGGAAATACTCTAG	3045
Spimp_LA1589_RDR	TGCCATCGGTTTTGCAGGAAATACTCTAG	3045
Sarc_LA2157_RDR	TGCCATCGGTTTTGCAGGAAATACTCTAG	3057
Spenn_LA716_RDR	TGCCATCGCTTTTGCAGGAAATACTCTAG	3057
Shabr_LYC4_RDR	TGCCATCGGTTTTGCAGGAAATACTCTAG	3000
Sper_RDR	TGCCATCGGTTTTGCAGGAAATACTCTAG	3057
Schil_LA1969_Ty1	TGCCATCGGTTTTGCAGGAAATACTCTAG	3057
Schil_LA2779_Ty3	TGCCATCGGTTTTGCAGGAAATACTCTAG	3057
Schil_Gh13_RDR	TACCATCGGTTTTGCAGGAAATACTCTAG	2836
Schil_BTI-87_RDR	TGCCATCGGTTTTGCAGGAAATACTCTAG	3045
Schil_LA1932_RDR	TGCCATCGGTTTTGCAGGAAATACTCTAG	3057
Schil_LA1938_RDR	TGCCATCGGTTTTGCAGGAAATACTCTAG	3057
Schil_LA1971_RDR	TGCCATCGGTTTTGCAGGAAATACTCTAG	3057
Schil_G1_1556_RDR	TGCCATCGGTTTTGCAGGAAATACTCTAG	3229
Schil_G1_1558_RDR	TGCCATCGGTTTTGCAGGAAATACTCTAG	3057
