# Supplementary material for: Assessing the genetic variation of Ty-1 and Ty-3 alleles conferring resistance to tomato yellow leaf curl virus in a broad tomato germplasm
Source: Mol Breed. 2015 May 26;35(6):132. doi: 10.1007/s11032-015-0329-y (PMC4442973; doi:10.1007/s11032-015-0329-y)
Supplement: Supplementary file 7 — Alignment of protein sequences of the Ty-1/Ty-3 RDR. Protein sequences are derived from cDNA sequences of accessions and derived lines as in Figure S6. The 5’ indel and the catalytic domain are highlighted in red. A premature stop codon in S. pennellii LA716 is highlighted in red. Three Ty-1/Ty-3-specific amino acids are highlighted in yellow; two Ty-3-specific amino acids are highlighted in green (PDF 111 kb) [file 11032_2015_329_MOESM7_ESM.pdf]

**Figure S7 RDR protein alignment**

```
Slyc_MM_RDR        MGDPLIEEIDV----LDAPLPYSVETMLDRICKEQGQKPPCTGIRRRLSSIGEKGSLEMLKIISRRPIKKSLSAFLVYMIDRYPDCLSSSSSPFNCLLKR   96
Slyc_M82_RDR       MGDPLIEEIDV----LDAPLPYSVETMLDRICKEQGQKPPCTGIRRRLSSIGEKGSLEMLKIISRRPIKKSLSAFLVYMIDRYPDCLSSSSSPFNCLLKR   96
Spimp_LA1589_RDR   MGDPLIEEIDV----LDAPLPYSVETMLDRICKEQGQKPPCTGIRRRLSSIGEKGSLEMLKIISRRPIKKSLSAFLVYMIDRYPDCLSSSSSPFNSLLKR   96
Sarc_LA2157_RDR    MGDPLIEEIDVPSCIVDAPLPYSVETMLDRICKEQGQKPPCTGIRRRLSSIGEKGSLEMLKIISRRPIKKSLSAFLVYMIDRYPDCLSSSSSPFNSLLKR  100
Spenn_LA716_RDR    MGDPFIEEIDVPSCILDAPLPYSVETMLERICKEQGQKPPCTGIRRRLSSIGEKGSLEMLKIISRRPIKKSLSAFLVYMIDRYPDCLSSSSSPFNSLLKR  100
Shabr_LYC4_RDR     MGDPFVEEIDVSSCILDAPLPYSVETMLERICKEQGQKPPCTGIRRRLSSIGEKGSLEMLKIISRRPIKKSLSAFLVYMIDRYPDCLSSSSSPFNSLLKR  100
Sper_RDR           MGDPLIEEMDVPSCILDAPLPYSVETMLDRICKEQGQKPPCTGIRRRLSSIGEKGSLEMLKIISRRPIKKTLSAFLVYMIDRYPDCLSSSSSPSNTLLKR  100
Schil_Ty1_MV       MGDPLIEEIDVPSCILDAPLPYSVETMLDRICKEQGQKPPCTGIRRRLSSIGEKGSLEMLKIISRRPIKKSLSAFLVYMIDRYPDCLSSSSSPFNSLLKR  100
Schil_Ty3_MV       MGDPLIEEIDVPSCILDAPLPYSVETMLDRICKEQGQKPPCTGIRRRLSSIGEKGSLEMLKIISRRPIKKSLSAFLVYMIDRYPDCLSSSSSPFNSLLKR  100
Schil_Gh13_RDR     --------------------------------------PPCTGIRRRLSSIGEKGSLEMLKIISRRPIKKSXXXX-------------------------   37
Schil_BTI-87_RDR   MGDPLIEEIDV----LDAPLPYSVETMLDRICKEQGQKPPCTRIRRRLSSIGEKGSLEMLKIISRRPIKKSLSAFLVYMIDRYPDCLSSSSSPFNSLLKR   96
Schil_LA1932_RDR   MGDPLIEEIDVPSCILDAPLPYSVETMLDRICKEQGQKPPCTGIRRRLSSIGEKGSLEMLKIISRRPIKKSLSAFLVYMIDRYPDCLSSSSSPSNSLLKR  100
Schil_LA1938_RDR   MGDPLIEEIDVPSCILDAPLPYSVETMLDRICKEQGQKPPCTGIRRRLSSIGEKGSLEMLKIISRRPIKKSLSAFLVYMIDRYPDCLSSSSSPFNSLLKR  100
Schil_LA1971_RDR   MGDPLIEEIDVPSCILDAPLPYSVETMLDRICKEQGQKPPCTGIRRRLSSIGEKGSLEMLKIISRRPIKKSLSAFLVYMIDRYPDCLSSSSSPSNSLLKR  100
Schil_G1_1556_RDR  MGDPLIEEXDVPSCILDAPLPYSVETMLDRICKEQGQKPPCTGIRRRLSSIGEKGSLEMLKIISRRPIKKXLSAFLVYMIDRYPDCLSSSSSPSNXLLKR  100
Schil_G1_1558_RDR  MGDPLIEEIDVPSCILDAPLPYSVETMLDRICKEQGQKPPCTGIRRRLSSIGEKGSLEMLKIISRRPIKKSLSAFLVYMIDRYPDCLSSSSSPSNSLLKR  100
                              indel

Slyc_MM_RDR        SSSPRLFPSPEGKRLQGESSSKSKLEMGLLACASPQKVARQLSFCEEPESNCRRTSPYVSQQLMILNELEFRKLFLVLSYIGCNKLEDVISPQIADDIVR  196
Slyc_M82_RDR       SSSPRLFPSPEGKRLQGESSSKSKLEMGLLACASPQKVARQLSFCEEPESNCRRTSPYVSQQLMILNELEFRKLFLVLSYIGCNKLEDVISPQIADDIVR  196
Spimp_LA1589_RDR   SSSPRLFPSPEGKRLQGESSSKSKLEMGLLACASPQKVARQLSFCEEPESNCRRTSPYVSQQLMILNELEFRKLFLVLSYIGCNKLEDVISPQIADDIVR  196
Sarc_LA2157_RDR    SSSPRLFPSPEGKRLQGESSSKSKLEMGLLACASPQKVACQLSFCEEPESNCRRTSPYVSQQLMILNELEFRKLFLVLSYIGCNKLEDVISPQIADDIVR  200
Spenn_LA716_RDR    SSSPLLFPSPEGKRVQGESSSKSKLEMGFLACASPQKVARQLSFCEEPESNSRRTSPYVSQQLMILNELEFRKLFLVLSYIGCNKLEDVISPQIADDIVR  200
Shabr_LYC4_RDR     SSSPLLFPSPEGKRVQGESSSKSKHEMGFLACASPQKVARQLSFCEEPESNCRRTSPYVSQQLMILNELEFRKLFLVLSYIGCNKLEDVISPQIADDIVR  200
Sper_RDR           SSSPLLFPSPEGKRLQGESSSKSKLEMGLLACASPQKVARQLSFCEEPESNCRRTSPYVSQQLMILNELEFRKLFLVLSYIGCNKLEDVISPQIADDIVR  200
Schil_Ty1_MV       SSSPVLFPSPEGKRLQGESSSKSKLEMGLLACASPQKVARQLSFCEEPESNCRRTSPYVSQQLMILNELEFRKLFLVLSYIGCNKLEDVISPQIADDIVR  200
Schil_Ty3_MV       SSSPLLFPSPEGKRLLGESSSKSKLEMGLLACASPQKVARQLSFCEEPESNCRRTSPYVSQQLMILNELEFRKLFLVLSYIGCNKLEDVISPQIADDIVR  200
Schil_Gh13_RDR     -----------GKRLLGESSSKSKLEMGLLACASPQKVARQLSFCEEPESNCRRTSPYVSQQLMILNELEFRKLFLVLSYIGCNKLEDVISPQIADDIVR  126
Schil_BTI-87_RDR   SSSPRLFPSPEGKRLQGESSSKSKLEMGLLACASPQKVARQLSFCEEPESNCRRTSPYVSQQLMILNELEFRKLFLVLSYIGCNKLEDVISPQIADDIVR  196
Schil_LA1932_RDR   SSSPLLFPSPEGKRLQGESSSKSKLEMGLLACASPQKVARQLSFCEEPESNCRRTSPYVSQQLMILNELEFRKLFLVLSYIGCNKLEEVISPQIADDIVR  200
Schil_LA1938_RDR   SSSPVLFPSPEGKRLQGESSSKSKLEMGLLACASPQKVARQLSFCEEPESNCRRTSPYVSQQLMILNELEFRKLFLVLSYIGCNKLEDVISPQIADDIVR  200
Schil_LA1971_RDR   SSSPVLFPSPEGKRLQGESSSKSKLEMGLLACASPQKVARQLSFCEDPEANCRRTSPYVSQQLMILNELEFRKLFLVLSYIGCNKLEDVISPQIADDIVR  200
Schil_G1_1556_RDR  SSSPLLFPSPEGKRLQGESSSKSKXEMGXLACASPQKVARQLSFCEEPESNCRRTSPYVSQQLMILNELEFRKLXLVLSYXGCNKLEDVISPQIADXIVR  200
Schil_G1_1558_RDR  SSSPVLFPSPEGKRLQGESSSKSKLEMGLLACASPQKVARQLSFCEEPESNCRRTSPYVSQQLMILNELEFRKLFLVLSYIGCNKLEDVISPQIADEIVR  200
                                 Q116L

Slyc_MM_RDR        KKNLSMTDFESEIWNAFGKACYAVSDRSKYLDWNCRKTHIYYCHIKQNGYCSFKGPYLNTLRTHLQRALGDDNVLIVKFVEDTSCANIILEEGILVGLRR  296
Slyc_M82_RDR       KKNLSMTDFESEIWNAFGKACYAVSDRSKYLDWNCRKTHIYYCHIKQNGYCSFKGPYLNTLRTHLQRALGDDNVLIVKFVEDTSCANIILEEGILVGLRR  296
Spimp_LA1589_RDR   KKNLSMTDFESEIWNAFGKACYAVSDRSKYLDWNCRKTHIYYCHIKQNGYCTFKGPYLNTLRTHLQRALGDDNVLIVKFVEDTSCANIILEEGILVGLRR  296
Sarc_LA2157_RDR    KKNLSMTDFESEIWNAFGKACYAVSDRSKYLDWNCRKTHIYYCHIKQNGYCTFKGPYLNTVRTHLQRALGDDNVLIVKFVEDTSCANVILEEGILVGLRR  300
Spenn_LA716_RDR    KNNLSMTDFESEIWNAFGKACYAVSDRSKYLDWNCRKTHIYYCHIKQNGYCTFKGPYLNTVRTHLQRALGDDNVLIVKFVEDTSCANIILEEGILVGLRR  300
Shabr_LYC4_RDR     KKNLSMTDFESEIWNAFGKACYAVSDRSKYLDWNCRKTHIYYCHIKQNGYCTFKGPYLNTARTHLQRALGDDNVLIVKFVEDTSCANIILEEGILVGLRR  300
Sper_RDR           KKNLSMTDFESEIWNAFGKACYAVSDRSKYLDWNCRKTHIYYCHIKQNGYCTFKGPYLNTARTHLQRALGDDNVLIVKFVEDTSCANIILEEGILVGLRR  300
Schil_Ty1_MV       KKDLSMTDFESEIWNAFGKACYAVSDRSKYLDWNCRKTHIYYCHIKQNGCCTFKGPYLNTARTHLQRALGDDNVLIVKFVEDTSCANIILEEGILVGLRR  300
Schil_Ty3_MV       KKDLSMTDFESEIWNAFGKACYAVSDRSKYLDWNCRKTHIYYCHIKQNGCCTFKGPYLNTARTHLQRALGDDNVLIVKFVEDTSCANIILEEGILVGLRR  300
Schil_Gh13_RDR     KKDLSMTDFESEIWNAFGKACYAVSDRSKYLDWNCRKTHIYYCHIKQNGCCTFKGPYLNTARTHLQRALGDDNVLIVKFVEDTSCANIILEEGILVGLRR  226
Schil_BTI-87_RDR   KKDLSMTDFESEIWNAFGKACYAVSDRSKYLDWNCRKTHIYYCHIKQNGCCTFKGPYLNTARTHLQRALGDDNVLIVKFVEDTSCANIILEEGILVGLRR  296
Schil_LA1932_RDR   KKNLSMTDFESEIWNAFGKACYAVSDRSKYLDWNCRKTHIYYCHIKQNGCCTFKGPYLNTARTHLQRALGDDNVLIVKFVEDTSCANIILEEGILVGLRR  300
Schil_LA1938_RDR   KKDLSMTDFESEIWNAFGKACYAVSDRSKYLDWNCRKTHIYYCHIKQNGCCTFKGPYLNTARTHLQRALGDDNVLIVKFVEDTSCANIILEEGILVGLRR  300
Schil_LA1971_RDR   KKDLSMTDFESEIWNAFGKACYAVSDRSKYLDWNCRKTHIYYCHIKQNGCCTFKGPYLNTARTHLQRALGDDNVLIVKFVEDTSCANIILEEGILVGLRR  300
Schil_G1_1556_RDR  KKNLSMTDFESEIWNAFGKACYAVSDRSKYLDWNCRKTHIYYCHIKQNGCCTFKGPYLNTARTHLQRALGDDNVLIVKFVEDTSCANIILEEGILVGLRR  300
Schil_G1_1558_RDR  KKNLSMTDFESEIWNAFGKACYAVSDRSKYLDWNCRKTHIYYCHIKQNGCCTFKGPYLNTARTHLQRALGDDNVLIVKFVEDTSCANIILEEGILVGLRR  300
```

```
Slyc_MM_RDR          YRFFVYKDDKERKKSPAMMKTKTASLKCYFVRFESIGTCNDGESYVFSTKTISQARCKFMHVHMVSNMAKYAARLSLILSKTIKLQTDLDSVTIERIEDI   396
Slyc_M82_RDR         YRFFVYKDDKERKKSPAMMKTKTASLKCYFVRFESIGTCNDGESYVFSTKTISQARCKFMHVHMVSNMAKYAARLSLILSKTIKLQTDLDSVTIERIEDI   396
Spimp_LA1589_RDR     YRFFVYKDDKERKKSPAMMKTKTASLKCYFVRFESIGTCNDGESYVFSTKTISQARCKFMHVHMVSNMAKYAARLSLILSKTIKLQTDLDSVTIERIEDI   396
Sarc_LA2157_RDR      YRFFVYKDDKERKKSPAMMKTKTASLKCYFVRFESIGTCNDGESYVFSTKTISQARCKFMHVHMVSNMAKYAARLSLILSKTIKLQTDLDSVTIERIEDI   400
Spenn_LA716_RDR      YRFFVYKDDKERKKSPAMMKTKTASLKCYFVRFESIGTCNDGESYVFSTKTISQARCKFMHVHMVSNMAKYAARLSLILSKTIKLQTDLDSVTIERIEDI   400
Shabr_LYC4_RDR       YRFFVYKDDKERKKSPAMMKTKTASLKCYFVRFESIGTCNDGESYVFSTKTISQARCKFMHVHMVSNMAKYAARLSLILSKTIKLQADLDSVTIERIEDI   400
Sper_RDR             YRFFVYKDDKERKKSPAMMKTKTASLKCYFVRFESIGTCDDGESYVFSTKTISQARCKFMHVHMVSNMAKYAARLSLILSKTIKLQADLDSVTIERIEDI   400
Schil_Ty1_MV         YRFFVYKDDKERKKSPAMMKTKTASLKCYFVRFESIGTCDDGESYVFSTKTISQARCKFMHVHMVSNMAKYAARLSLILSKTIKLQVDLDSVTIERIEDI   400
Schil_Ty3_MV         YRFFVYKDDKERKKSPAMMKTKTASLKCYFVRFESIGTCDDGESYVFSTITISQARCKFMHVHMVSNMAKYAARLSLILSKTIKLQVDLDSVTIERIEDI   400
Schil_Gh13_RDR       YRFFVYKDDKERKKSPAMMKTKTASLKCYFVRFESIGTCDDGESYVFSTITISQARCKFMHVHMVSNMAKYAARLSLILSKTIKLQTDLDSVTIERIEDI   326
Schil_BTI-87_RDR     YRFFVYKDDKERKKSPAMMKTKTASLKCYFVRFESIGTCDDGESYVFSTKTISQARCKFMHVHMVSNMAKYAARLSLILSKTIKLQADLDSVTIERIEDI   396
Schil_LA1932_RDR     YRFFVYKDDKERKKSPAMMKTKTASLKCYFVRFESIGTCDDGESYVFSTKTISQARCKFMHVHMVSNMAKYAARLSLILSKTIKLQADLDSVTIERIEDI   400
Schil_LA1938_RDR     YRFFVYKDDKERKKSPAMMKTKTASLKCYFVRFESIGTCDDGESYVFSTKTISQARCKFMHVHMVSNMAKYAARLSLILSKTIKLQVDLDSVTIERIEDI   400
Schil_LA1971_RDR     YRFFVYKDDKERKKSPAMMKTKTASLKCYFVRFESIGTCDDGESYVFSTKTVSQARCKFMHVHMVSNMAKYAARLSLILSKTIKLQVDLDSVTIERIEDI   400
Schil_G1_1556_RDR    YRFFVYKDDKERKKSPAMMKTKTASLKCYFVRFESIGTCDDGESYVFSTKTISQARCKFMHVHMVSNMAKYAARLSLILSKTIKLQVDLDSVTIERIEDI   400
Schil_G1_1558_RDR    YRFFVYKDDKERKKSPAMMKTKTASLKCYFVRFESIGTCDDGESYVFSTKTISQARCKFMHVHMVSNMAKYAARLSLILSKTIKLQVDLDSVTIERIEDI   400
                                                                       K350I

Slyc_MM_RDR          LCRDENGCIIQDEDGEPRIHTDGTGFISEDLAMHCPKDFSKAEYIKDENYENFVDIVDLDDVNVERRVSVSRNRKPPLLMQCRLFFNGCAVKGTFLVNRK   496
Slyc_M82_RDR         LCRDENGCIIQDEDGEPRIHTDGTGFISEDLAMHCPKDFSKAEYIKDENYENFVDIVDLDDVNVERRVSVSRNRKPPLLMQCRLFFNGCAVKGTFLVNRK   496
Spimp_LA1589_RDR     LCRDENGCIIQDEDGEPRIHTDGTGFISEDLAMHCPKDFSKAEYIKDENYENFVDIVDLDDVNVERRVSVSRNREPPLLMQCRLFFNGCAVKGTFLVNRK   496
Sarc_LA2157_RDR      LCRDENGCIIQDEDGEPRIHSDGTGFISEDLAMHCPKDFSKAEYIKDENYENFVDIVDLDDVNVERRVSVSRNREPPLLMQCRLFFNGCAVKGTFLVNRK   500
Spenn_LA716_RDR      LCRDENGCIIQDEDGEPRIHTDGTGFISEDLAMHCPKDFSKAEYIKDENYENFVDIVDLDDVNVERRASVSRNREPPLLMQCRLFFNGCAVKGTFLVNRK   500
Shabr_LYC4_RDR       LCRDENGCIIQDEDGEPRIHTDGTGFISEDLAMHCPKDFSKAEYIKDENYEXXXX-------------------EPPLLMQCRLFFNGCAVKGTFLVNRK   481
Sper_RDR             LCRDENGCIIQDEDGEPRIHTDGTGFISEDLAMHCPKDFSKAEYIKDENYENFVDIVDLDDVNVERRASVSGNREPPLLMQCRLFFNGCAVKGTFLVNRK   500
Schil_Ty1_MV         LCRDENGCIIQDEDGEPRIHTDGTGFISEDLAMHCPKDFSKAEYIKDENYENFVDIVDLDDVNVERRASVSGNREPPLLMQCRLFFNGCAVKGTFLVNRK   500
Schil_Ty3_MV         LCRDENGCIIQDEDGEPRIHTDGTGFISEDLAMHCPKDFSKAEYIKDENYENFVDIVDLDDVNVERRASVSGNREPPLLMQCRLFFNGCAVKGTFLVNRK   500
Schil_Gh13_RDR       LCRDENGCIIQDEDGEPRIHTDGTGFISEDLAMHCPKDFSKAEYIKDENYENFVDIVDLDDVNVERRASVSGNREPPLLMQCRLFFNGCAVKGTFLVNRK   426
Schil_BTI-87_RDR     LCRDENGCIIQDEDGEPRIHTDGTGFISEDLAMHCPKDFSKAEYIKDENYENFVDIVDLDDVNVERRXSVSRNRKPPLLMQCRLFFKGCAVKGTFLLNRK   496
Schil_LA1932_RDR     LCRDENGCIIQDEDGEPRIHTDGTGFISEDLAMHCPKDFSKAEYIKDENYENFVDIVDLDDVNVERRASVSGKREPPLLMQCRLFFKGCAVKGTFLLNRK   500
Schil_LA1938_RDR     LCRDENGCIIQDEDGEPRIHTDGTGFISEDLAMHCPKDFSKAEYIKDENYENFVDIVDLDDVNVERRASVSGNREPPLLMQCRLFFNGCAVKGTFLVNRK   500
Schil_LA1971_RDR     LCQDENGCIIQDEDGEPRIHTDGTGFISEDLAMHCPKDFSKAEYIKDENYENFVDIVDLDDVNVERRASVSGNREPPLLMQCRLFFNGCAVKGTFLVNRK   500
Schil_G1_1556_RDR    LCRDENGCIIQDEDGEPRIHTDGTGFISEDLAMHCPKDFSKAEYIKDENYENFVDIVDLDDVNVERRVSVSRNREPPLLMQCRLFFNGCAVKGTFLVNRK   500
Schil_G1_1558_RDR    LCRDKNGCIIQDEDGEPRIHTDGTGFISEDLAMHCPKDFSKAEYIKDENYENFVDIVDLDDVNVERRASVSRNREPPLLMQCRLFFNGCAVKGTFLVNRK   500

Slyc_MM_RDR          IGSRKIHIRPSMVKVEIDPTISSIPTFDSLEIVAISHRPNKAYLSKNLISLLSYGGVHKEYFMELLGSALEETKQVYLRKRAALKVAINYREMDDECLTA   596
Slyc_M82_RDR         IGSRKIHIRPSMVKVEIDPTISSIPTFDSLEIVAISHRPNKAYLSKNLISLLSYGGVHKEYFMELLGSALEETKQVYLRKRAALKVAINYREMDDECLTA   596
Spimp_LA1589_RDR     IGSRKIHIRPSMVKVEIDPTISSIPTFDSLEIVAISHRPNKAYLSKNLISLLSYGGVHKEYFMELLGSALEETKQVYLRKRAALKVAINYREMDDECLTA   596
Sarc_LA2157_RDR      IGSRKIHIRPSMVKVEIDPTISSIPTFDSLEIVAISHRPNKAYLSKNLISLLSYGGVHKEYFMELLGSALEETNQVYLRKRAALKVAINYREMDDECLTA   600
Spenn_LA716_RDR      IGSRKIHIRPSMVKVEIDPTISSIPTFDSLEIVAISHRPNKAYLSKNLISLLSYGGVHKEYFMELLGSALEETKQVRLRKRAALKVAINYREMDDECLTA   600
Shabr_LYC4_RDR       IGSRKIHIRPSMVKVEIDPTISSIPTFDSLEIVAISHRPNKAYLSKNLISLLSYGGVHKEYFMELLGSALEETKQVYLRKRAALKVAINYREMDDECLTA   581
Sper_RDR             IGSRKIHIRPSMVKVEIDPTISSIPTFDSLEIVAISHRPNKAYLSKNLISLLSYGGVHREYFMELLGSALEETNQVYLRKRAALKVAINYREMDDECLTA   600
Schil_Ty1_MV         IGSRKIHIRPSMVKVEIDPTISSIPTFDSLEIVAISHRPNKAYLSKNLISLLSYGGVHKEYFLELLGSALEETKQVYLRKRAALKVAINYREMDDECLTA   600
Schil_Ty3_MV         IGSRKIHIRPSMVKVEIDPTISSIPTFDSLEIVAISHRPNKAYLSKNLISLLSYGGVHKEYFLELLGSALEETKQVYLRKRAALKVAINYREMDDECLTA   600
Schil_Gh13_RDR       IGSRKIHIRPSMVKVEIDPTISSIPTFDSLEIVAISHRPNKAYLSKNLISLLSYGGVHKEYFLELLGSALEETKQVYLRKRAALKVAINYREMDDECLTA   526
Schil_BTI-87_RDR     IGSRKIHIRPSMVKVEIDPTISSIPTFDSLEIVAISHRPNKAYLSKNLISLLSYGGVHKEYFLELLGSALEETKQVYLRKRAALKVAINYREMDDECLTA   596
Schil_LA1932_RDR     IGSRKIHIRPSMVKVEIDPTISSIPTFDSLEIVAISHRPNKAYLSKNLISLLSYGGVHKEYFLELLGSALEETKQVYLRKRAALKVAINYREMDDECLTA   600
Schil_LA1938_RDR     IGSRKIHIRPSMVKVEIDPTISSIPTFDSLEIVAISHRPNKAYLSKNLISLLSYGGVHKEYFLELLGSALEETKQVYLRKRAALKVAINYREMDDECLTA   600
Schil_LA1971_RDR     IGSRKIHIRPSMVKVEIDPTISSIPTFDSLEIVAISHRPNKAYLSKNLISLLSYGGVHKEYFLELLGSALEETKQVYLRKRAALKVAINYREMDDECLTA   600
Schil_G1_1556_RDR    IGSRKIHIRPSMVKVEIDPTISSISTFDSLEIVAISHRPNKAYLSKNLISLLSYGGVHKEYFMELLGSALEETNQVYLRKRAALKVAINYREMDDECLTA   600
Schil_G1_1558_RDR    IGSRKIHIRPSMVKVEIDPTISSISTFDSLEIVAISHRPNKAYLSKNLISLLSYGGVHKEYFMELLGSALEETNQVYLRKRAALKVAINYREMDDECLTA   600
                                                                                         M563L
```

```
Slyc_MM_RDR          RMISSGIPLNEPHLHARLSRLAKIERTKLRGGKLPISDSFYLMGTADPTGVLESNEVCVILDNGQVSGRVLVYRNPGLHFGDVHVMKARYVEELADVVGD   696
Slyc_M82_RDR         RMISSGIPLNEPHLHARLSRLAKIERTKLRGGKLPISDSFYLMGTADPTGVLESNEVCVILDNGQVSGRVLVYRNPGLHFGDVHVMKARYVEELADVVGD   696
Spimp_LA1589_RDR     RMISSGIPLNEPHLHARLSRLAKIERTKLRGGKLPISDSFYLMGTADPTGVLESNEVCVILDNGQVSGRVLVYRNPGLHFGDVHVMKARYVEELADVVGD   696
Sarc_LA2157_RDR      RMISSGIPLNEPHLHARLSRLAKIERTKLRGGKLPISDSFYLMGTADPTGVLESNEVCVILDNGQVSGRVLVYRNPGLHFGDVHVMKARYVEELADVVGD   700
Spenn_LA716_RDR      RMISSGIPLNEPHLHARLSRLAKIERTKLRGGKLPINDSFYLMGTADPTGVLESNEVCVILDNGQVSGRVLVYRNPGLHFGDVHVMKARYVEELADVVGD   700
Shabr_LYC4_RDR       RMISSGIPLNEPHLHARLSRLAKIERTKLRGGKLPISDSFYLMGTADPTGVLESNEVCVILDNGQVSGRVLVYRNPGLHFGDVHVMKARYVEELADVVGD   681
Sper_RDR             RMISSGIPLNEPHLHARLSRLAKIERTKLRGGKLPISDSFYLMGTADPTGVLESNEVCVILDNGQVSGRVLVYRNPGLHFGDVHVMKARYVEELADVVGD   700
Schil_Ty1_MV         RMISSGIPLNEPHLHVRLSRLAKIERTKLRGGKLPISDSFYLMGTADPTGVLESNEVCVILDNGQVSGRVLVYRNPGLHFGDVHVMKARYVEELADVVGD   700
Schil_Ty3_MV         RMISSGIPLNEPHLHVRLSRLAKIERTKLRGGKLPISDSFYLMGTADPTGVLESNEVCVILDNGQVSGRVLVYRNPGLHFGDVHVMKARYVEELADVVGD   700
Schil_Gh13_RDR       RMISSGIPLNEPHLHVRLSRLAKIERTKLRGGKLPISDSFYLMGTADPTGVLESNEVCVILDNGQVSGRVLVYRNPGLHFGDVHVMKARYVEELADVVGD   626
Schil_BTI-87_RDR     RMISSGIPLNEPHLHVRLSRLAKIERTKLRGGKLPISDSFYLMGTADPTGVLESNEVCVILDNGQVSGRVLVYRNPGLHFGDVHVMKARYVEELADVVGD   696
Schil_LA1932_RDR     RMISSGIPLNEPHLHVRLSRLAKIERTKLRGGKLPISDSFYLMGTADPTGVLESNEVCVILDNGQVSGRVLVYRNPGLHFGDVHVMKARYVEELADVVGD   700
Schil_LA1938_RDR     RMISSGIPLNEPHLHVRLSRLAKIERTKLRGGKLPISDSFYLMGTADPTGVLESNEVCVILDNGQVSGRVLVYRNPGLHFGDVHVMKARYVEELADVVGD   700
Schil_LA1971_RDR     RMISSGIPLNEPHLHVRLSRLAKIERTKLRGGKLPISDSFYLMGTADPTGVLESNEVCVILDNGQVSGRVLVYRNPGLHFGDVHVMKARYVEELADVVGD   700
Schil_G1_1556_RDR    RMISSGIPLNEPHLHARLSRLAKIERTKLRGGKLPISDSFYLMGTADPTGVLESNEVCVILDNGQVSGRVLVYRNPGLHFGDVHVMKARYVEELADVVGD   700
Schil_G1_1558_RDR    RMISSGIPLNEPHLHARLSRLAKIERTKLRGGKLPISDSFYLMGTADPTGVLESNEVCVILDNGQVSGRVLVYRNPGLHFGDVHVMKARYVEELADVVGD   700
                                    A616V

Slyc_MM_RDR          AKYGIFFSTKGPRSAATEIANGDFDGDMYWVSINRKLVDSYTTSRPWIRMHSTPNAVSKKPSEFSADELEYELFRQFLEAKSKGANMSLAADSWLAFMDR   796
Slyc_M82_RDR         AKYGIFFSTKGPRSAATEIANGDFDGDMYWVSINRKLVDSYTTSRPWIRMHSTPKAVSKKPSEFSADELEYELFRQFLEAKSKGANMSLAADSWLAFMDR   796
Spimp_LA1589_RDR     AKYGIFFSTKGPRSAATEIANGDFDGDMYWVSINRKLVDSYTTSRPWIRMHSTPKAVSKKPSEFSADELEYELFRQFLEAKSKGANMSLAADSWLAFMDH   796
Sarc_LA2157_RDR      AKYGIFFSTKGPRSAATEIANGDFDGDMYWVSINRKVVDSYTTSRPWIRMHSTPKAVSKKPSEFSADELEYELFRQFLEAKSKGANMSLAADSWLAFMDR   800
Spenn_LA716_RDR      AKYGIFFSTKGPR*AATEIANGDFDGDMYWVSINRKVVDSYTTSRPWIRMHSTPKAVSKKPSEFSADELEYELFRQFLEAKSKGANMSLAADSWLAFMDR   800
Shabr_LYC4_RDR       AKYGIFFSTKGPRSAATEIANGDFDGDMYWVSINRKVVDSYTTSRPWIRMHSTPKAVSKKPSEFSADELEYELFRQFLEAKSKGANMSLAADSWLAFMDR   781
Sper_RDR             AKYGIFFSTKGPRSAATEIANGDFDGDMYWVSINRKVVDSYTTSRPWIRMHSTPKAVSKKPSEFSADELEYELFRQFLEAKSKGANMSLAADSWLAFMDR   800
Schil_Ty1_MV         AKYGIFFSTKGPRSAATEIANGDFDGDMYWVSINRKLVDSYTTSRPWIRMHSTPKAVSKKPSEFSADELEYELFRQFLEAKSKGANMSLAADSWLAFMDR   800
Schil_Ty3_MV         AKYGIFFSTKGPRSAATEIANGDFDGDMYWVSINRKLVDSYTTSRPWIRMHSTPKAVSKKPSEFSADELEYELFRQFLEAKSKGANMSLAADSWLAFMDR   800
Schil_Gh13_RDR       AKYGIFFSTKGPRSAATEIANGDFDGDMYWVSINRKLVDSYTTSRPWIRMHSTPKAVSKKPSEFSADELEYELFRQFLEAKSKGANMSLAADSWLAFMDR   726
Schil_BTI-87_RDR     AKYGIFFSTKGPRSAATEIANGDFDGDMYWVSINRKLVDSYTTSRPWIRMHSTPKAVSKKPSEFSADELEYELFRQFLEAKSKGANMSLAADSWLAFMDR   796
Schil_LA1932_RDR     AKYGIFFSTKGPRSAATEIANGDFDGDMYWVSINRKLVDSYTTSRPWIRIHSTPKAVSKKPSEFSADELEYELFRQFLEAKSKGANMSLAADSWLAFMDR   800
Schil_LA1938_RDR     AKYGIFFSTKGPRSAATEIANGDFDGDMYWVSINRKLVDSYTTSRPWIRMHSTPKAVSKKPSEFSADELEYELFRQFLEAKSKGANMSLAADSWLAFMDR   800
Schil_LA1971_RDR     AKYGIFFSTKGPRSAATEIANGDFDGDMYWVSINRKLVDSYTTSRPWIRMHSTPKAVSKKPSEFSADELEYELFRQFLEAKSKGANMSLAADSWLAFMDR   800
Schil_G1_1556_RDR    AKYGIFFSTKGPRSAATEIANGDFDGDMYWVSINRKVVDSYTTSRPWIRMHSTPKAVSKKPSEFSADELEYELFRQFLEAKSKGANMSLAADSWLAFMDR   800
Schil_G1_1558_RDR    AKYGIFFSTKGPRSAATEIANGDFDGDMYWVSINRKVVDSYTTSRPWIRMHSTPKAVSKKPSEFSADELEYELFRQFLEAKSKGANMSLAADSWLAFMDR   800
                                  S714*  Catalytic domain

Slyc_MM_RDR          LLTLRDDNVDDMHSLKGKMLHLIDIYYDALDAPKSGKKVSIPHYLKANKFPHYMEKGNSCSYHSTSILGQIYDHVDSYPDEDLCITEISKLPCFEVEIPQ   896
Slyc_M82_RDR         LLTLRDDNVDDMHSLKGKMLHLIDIYYDALDAPKSGKKVSIPHYLKANKFPHYMEKGNSCSYHSTSILGQIYDHVDSYPDEDLCITEISKLPCFEVEIPQ   896
Spimp_LA1589_RDR     LLTLRDDNVDDMHSLKGKMLHLIDIYYDALDAPKSGKKVSIPHYLKANKFPHYMEKGNSCSYHSTSILGQIYDHVDSYPDEDLCITEISKLPCFEVEIPQ   896
Sarc_LA2157_RDR      LLTLRDDNVDDMHSLKGKMLHLIDIYYDALDAPKSGKKVSIPHYLKANKFPHYMEKGNSCSYHSTSILGQIYDHVDSYPDEDLCITEISKLPCFEVEIPQ   900
Spenn_LA716_RDR      LLMLRDDNVDDMHSLKGKMLHLIDIYYDALDAPKSGKKVSIPHYLKANKFPHYMEKGNSCSYHSTSILGQIYDHVDSYPDEDLCITEISKLPCFEVEIPQ   900
Shabr_LYC4_RDR       LLMLRDDNVDDMHSLKGKMLHLIDIYYDALDAPKSGKKVSIPHYLKANKFPHYMEKGNSCSYHSTSILGQIYDHVDSYPDEDLCITEISKLPCFEVEIPQ   881
Sper_RDR             LLMLRDDNVDDMHSLKGKMLHLIDIYYDALDAPKSGKKVSIPHYLKANKFPHYMEKGNSCSYHSTSILGQIYDHVDSYPDEDLCITEISKLPCFEVEIPQ   900
Schil_Ty1_MV         LLMLRDDNVDDMHSLKGKMLHLIDIYYDALDAPKSGKKVSIPHYLKANKFPHYMEKGNSCSYHSTSILGQIYDHVDSYPDEDLCITEISKLPCFEVEIPQ   900
Schil_Ty3_MV         LLMLRDDNVDDMHSLKGKMLHLIDIYYDALDAPKSGKKVSIPHYLKANKFPHYMEKGNSCSYHSTSILGQIYDHVDSYPDEDLCITEISKLPCFEVEIPQ   900
Schil_Gh13_RDR       LLMLRDDNVDDMHSLKGKMLHLIDIYYDALDAPKSGKKVSIPHYLKANKFPHYMEKGNSCSYHSTSILGQIYDHVDSYPDEDLCITEISKLPCFEVEIPQ   826
Schil_BTI-87_RDR     LLMLRDDNVDDMHRLKGKMLHLIDIYYDALDAPKSGKKVSIPHYLKANKFPHYMEKGNSCSYHSTSILGQIYDHVDSYPDEDLCITEISKLPCFEVEIPQ   896
Schil_LA1932_RDR     LLMLRDDNVDDMHSLKGKMLHLIDIYYDALDAPKSGKKVSIPHYLKANKFPHYMEKGNSCSYHSTSILGQIYDHVDSYPDEDLCITEISKLPCFEVEIPQ   900
Schil_LA1938_RDR     LLMLRDDNVDDMHSLKGKMLHLIDIYYDALDAPKSGKKVSIPHYLKANKFPHYMEKGNSCSYHSTSILGQIYDHVDSYPDEDLCITEISKLPCFEVEIPQ   900
Schil_LA1971_RDR     LLMLRDDNVDDMHSLKGKMLHLIDIYYDALDAPKSGKKVSIPHYLKANKFPHYMEKGNSCSYHSTSILGQIYDHVDSYPDEDLCITEISKLPCFEVEIPQ   900
Schil_G1_1556_RDR    LLTLRDDNVDDMHSLKGKMLHLIDIYYDALDAPKSGKKVSIPHYLKANKFPHYMEKGNSCSYHSTSILGQIYDHVDSYPDEDLCITG-------------   887
Schil_G1_1558_RDR    LLTLRDDNVDDMHSLKGKMLHLIDIYYDALDAPKSGKKVSIPHYLKANKFPHYMEKGNSCSYHSTSILGQIYDHVDSYPDEDLCITEISKLPCFEVEIPQ   900
```

```
Slyc_MM_RDR          RCMTLWRGRYEEYKKDMTRAMNFDCELRITSCNEVIKKYKMLLYGAVEFEQTVRKTEDIFDEALAIYHVTYDNARITYSIEKCGFAWKVAGSALCRIHAM   996
Slyc_M82_RDR         RCMTLWRGRYEEYKKDMTRAMNFDCELRITSCNEVIKKYKMLLYGAVEFEQTVRKTEDIFDEALAIYHVTYDNARITYSIEKCGFAWKVAGSALCRIHAM   996
Spimp_LA1589_RDR     RCMTLWRGRYEEYKKDMTRAMNFDCELRITSCNEVIKKYKMLLYGAVEFEQTVRKTEDIFDEALAIYHVTYDNARITYSIEKCGFAWKVAGSALCRIHAM   996
Sarc_LA2157_RDR      RCMTLWRGRYEEYKKDMTRAMNLDCELRITSCNEVIKKYKMLLYGAVEFEQTVRKTEDIFDEALAIYHVTYDNARITYSIEKCGFAWKVAGSALCRIHAM  1000
Spenn_LA716_RDR      RCMTLWRGRYEEYKKDMTRAMNLDCELRITSCNEVIKKYKMLLYGAVEFEQTVRKTEDIFDEALAIYHVTYDNARIAYSIEKCGFAWKVAGSALCRIHAM  1000
Shabr_LYC4_RDR       RCMTLWRGRYEEYKKDMTRAMNLDCELRITSCNEVIKKYKMLLYGAVEFEQTVRKTEDIFDVALAIYHVTYDNARIAYSIEKCGFAWKVAGSALCRIHAM   981
Sper_RDR             RCMTLWRGRYEEYKKDMTRAMNLDCELRITSCNEVIKKYKMLLYGAVEFEQTVRKTEDIFDEALAIYHVTYDNARISYSIEKCGFAWKVAGSALCRIHAM  1000
Schil_Ty1_MV         RCMTLWRGRYEEYKKDMTQAMNLDCELRITSCNEVIKKYKMLLYGAVEFEQTVRKTEDIFDEALAIYHVTYDNARITYSIEKCGFAWKVAGSALCRIHAM  1000
Schil_Ty3_MV         RCMTLWRGRYEEYKKDMTQAMNLDCELRITSCNEVIKKYKMLLYGAVEFEQTVRKTEDIFDEALAIYHVTYDNARITYSIEKCGFAWKVAGSALCRIHAM  1000
Schil_Gh13_RDR       RCMTLWRGRYEEYKKDMTQAMNLDCELRITSCNEVIKKYKMLLYGAVEFEQTVRKTEDIFDEALAIYHVTYDNARITYSIEKCGFAWKVAGSALCRIHAM   926
Schil_BTI-87_RDR     RCMTLWRGRYEEYKKDMTQAMNLDCELRITSCNEVIKKYKMLLYGAVEFEQTVRKTEDIFDEALAIYHVTYDNARITYSIEKCGFAWKVAGSALCRIHAM   996
Schil_LA1932_RDR     RCMTLWRGRYEEYKKDMTQAMNLDCELRITSCNEVIKKYKMLLYGAVEFEQTVRKTEDIFDEALAIYHVTYDNARITYSIEKCGFAWKVAGSALCRIHAM  1000
Schil_LA1938_RDR     RCMTLWRGRYEEYKKDMTQAMNLDCELRITSCNEVIKKYKMLLYGAVEFEQTVRKTEDIFDEALAIYHVTYDNARITYSIEKCGFAWKVAGSALCRIHAM  1000
Schil_LA1971_RDR     RCMTLWRGRYEEYKKDMTQAMNLDCELRITSCNEVIKKYKMLLYGAVEFEQTVRKTEDIFDEALAIYHVTYDNARIAYSIEKCGFAWKVAGSALCRIHAM  1000
Schil_G1_1556_RDR    ----------------------------------------------------------------------------------------------------   887
Schil_G1_1558_RDR    RCMTLWRGRYEEYKKDMTRAMNLDCELRITSCNEVIKKYKMLLYGAVEFEQTVRKTEDIFDEALAIYHVTYDNARISYSIEKCGFAWKVAGSALCRIHAM  1000
                                       R919Q

Slyc_MM_RDR          YRKEKDLPILPSVLQEIL   1014
Slyc_M82_RDR         YRKEKDLPILPSVLQEIL   1014
Spimp_LA1589_RDR     YRKEKDLPILPSVLQEIL   1014
Sarc_LA2157_RDR      YRKEKDLPILPSVLQEIL   1018
Spenn_LA716_RDR      YRKEKDLPILPSLLQEIL   1018
Shabr_LYC4_RDR       YRKEKDLPILPSVLQEIL    999
Sper_RDR             YRKEKDLPILPSVLQEIL   1018
Schil_Ty1_MV         YRKEKDLPILPSVLQEIL   1018
Schil_Ty3_MV         YRKEKDLPILPSVLQEIL   1018
Schil_Gh13_RDR       YRKEKDLPILPSVLQEIL    944
Schil_BTI-87_RDR     YHKEKDLPILPSVLQEIL   1014
Schil_LA1932_RDR     YHKEKDLPILPSVLQEIL   1018
Schil_LA1938_RDR     YRKEKDLPILPSVLQEIL   1018
Schil_LA1971_RDR     YRKEKDLPILPSVLQEIL   1018
Schil_G1_1556_RDR    ------------------    887
Schil_G1_1558_RDR    YRKEKDLPILPSVLQEIL   1018
```
